# Causal associations between female reproductive behaviors and psychiatric disorders: a lifecourse Mendelian randomization study

**DOI:** 10.1186/s12888-023-05203-y

**Published:** 2023-11-02

**Authors:** Yifan Yu, Lei Hou, Yutong Wu, Yuanyuan Yu, Xinhui Liu, Sijia Wu, Yina He, Yilei Ge, Yun Wei, Fengtong Qian, Qingxin Luo, Yue Feng, Xiaojing Cheng, Tiangui Yu, Hongkai Li, Fuzhong Xue

**Affiliations:** 1 Department of Epidemiology and Health Statistics, School of Public Health, , Cheeloo College of Medicine, Shandong University, 44 Wenhua West Road, Jinan, Shandong Province China; 2 Institute for Medical Dataology, Cheeloo College of Medicine, Shandong University, Jinan, People’s Republic of China; 3 Beijing International Center for Mathematical Research, Peking University, Beijing, People’s Republic of China; 4 Shandong Mental Health Center, Shandong Province, Jinan, China; 5 Qilu Hospital, Cheeloo College of Medicine, Shandong University, Jinan, People’s Republic of China

**Keywords:** Reproductive behaviors, Psychiatric disorders, Genetic correlation, Mendelian randomization

## Abstract

**Background:**

The timings of reproductive life events have been examined to be associated with various psychiatric disorders. However, studies have not considered the causal pathways from reproductive behaviors to different psychiatric disorders. This study aimed to investigate the nature of the relationships between five reproductive behaviors and twelve psychiatric disorders.

**Methods:**

Firstly, we calculated genetic correlations between reproductive factors and psychiatric disorders. Then two-sample Mendelian randomization (MR) was conducted to estimate the causal associations among five reproductive behaviors, and these reproductive behaviors on twelve psychiatric disorders, using genome-wide association study (GWAS) summary data from genetic consortia. Multivariable MR was then applied to evaluate the direct effect of reproductive behaviors on these psychiatric disorders whilst accounting for other reproductive factors at different life periods.

**Results:**

Univariable MR analyses provide evidence that age at menarche, age at first sexual intercourse and age at first birth have effects on one (depression), seven (anxiety disorder, ADHD, bipolar disorder, bipolar disorder II, depression, PTSD and schizophrenia) and three psychiatric disorders (ADHD, depression and PTSD) (based on *p*<7.14×10^-4^), respectively. However, after performing multivariable MR, only age at first sexual intercourse has direct effects on five psychiatric disorders (Depression, Attention deficit or hyperactivity disorder, Bipolar disorder, Posttraumatic stress disorder and schizophrenia) when accounting for other reproductive behaviors with significant effects in univariable analyses.

**Conclusion:**

Our findings suggest that reproductive behaviors predominantly exert their detrimental effects on psychiatric disorders and age at first sexual intercourse has direct effects on psychiatric disorders.

**Supplementary Information:**

The online version contains supplementary material available at 10.1186/s12888-023-05203-y.

## Background

Psychiatric disorders have become the major contributors to overall morbidity and disability across the globe [1]. Approximately 30% of individuals suffer from psychiatric disorders across the lifespan [2] and the causes of psychiatric disorders may be different at different stages of life. Since psychiatric disorder is one of the leading cause of disease burdens, it is necessary to identify potential risk factors for further prevention [3].

Female reproductive behaviors, including age at menarche, age at first sexual intercourse, age at first birth, age at last birth and age at menopause, have important implications in reproductive health and evolutionary fitness. Some of these reproductive behaviors have been identified as risk factors for various psychiatric disorders [4–6], such as depression, schizophrenia [7] and bipolar disorders [8]. Taking depression as an example, multiple studies in the past decade have examined the association between age of menarche and risk of depression in adolescent girls and adulthood [9–13]. For instance, a mendelian randomization analysis showed early menarche is associated with higher levels of depressive symptoms at 14 years old [11]. Other studies have implicated early age at first sexual intercourse and early age at first birth were risk factors of depression [14–17]. A recent study used the National Longitudinal Study of Adolescent Health dataset to show earlier age of first coitus was associated with depressive symptoms [14].

However, the potential role of a woman’s reproductive behaviors in the causal pathways between reproductive behaviors and psychiatric disorders from the perspective of the whole life-course remains unclear. Whether an individual can reverse the impact of age of early reproductive behaviors (such as menarche) on psychiatric disorders through lifestyle modifications is unclear, particularly as those who had earlier age at menarche tend to have earlier ages at other reproductive behaviors than those with a later age of menarche [18–22]. For example, a meta-analysis study showed that early menarche increases the risk of premature and early menopause by 80% [20]. This makes it challenging to distinguish whether reproductive behaviors in early life have independent and lasting effects on psychiatric disorders, or whether its effects are partially mediated by other reproductive behaviors in later years. If the latter is the case, then it is possible to avoid the potential adverse consequences of earlier reproductive behaviors by attaining a different reproductive lifestyle in the later years.

Mendelian randomization (MR) is an approach that can deal with these challenges by using genetic variants as instrumental variables (IV) to infer causality among correlated traits. A valid IV must satisfy the following three assumptions: 1) Relevance: IV is robustly related to the exposure; 2) Exchangeability: IV is independent of unmeasured confounders of the exposure and outcome relationship; and 3) Exclusion restriction: IV affects the outcome only through the exposure [23]. The advantage of MR is that it could eliminate the impact of confounding and reverse causality compared with traditional regression analysis [24, 25]. In addition, multivariable MR is recently developed to determine whether an exposure affects the outcome under the condition of other exposures [26] which makes it possible to explore the causal pathways between the psychiatric disorder and reproductive behaviors.

In this MR study, we evaluated causal relationships between the five reproductive behaviors based on their temporal order, and explored whether genetically predicted reproductive behaviors have effects on twelve psychiatric disorders, using genome-wide association studies (GWAS) summary data of reproductive behaviors and psychiatric disorders on large sample populations that are publicly available. For psychiatric disorders with strong evidence of genetically predicted effects with multiple reproductive behaviors, we applied multivariable MR to examine evidence of direct or indirect effects by accounting for reproductive behaviors with significant effects.

## Methods

### Data sources

#### Reproductive factors

The reproductive factors investigated in this study includes age at menarche (AgeMenarche), age at first sexual intercourse (AgeFirstSex), age at first birth (AgeFirstBirth), age at last birth (AgeLastBirth) and age at menopause (AgeMenopause). GWAS summary statistics for age of menarche [27] and menopause [28] were from ReproGen consortium, including 252,000 and 201,323 participants respectively. For AgeFirstSex and AgeFirstBirth, we used the summary statistics from the GWAS meta-analysis of 36 cohorts by Day et al*.* [29] study, including 542,901 and 418,758 individuals respectively. GWAS summary statistics for AgeLastBirth were from UK Biobank cohort [30] with the sample size of 170,248. These reproductive factors were restricted in European ancestry. Details for datasets are listed in Table 1. We selected SNPs at a GWAS threshold of statistical significance with $p<5\times {10}^{-8}$ and linkage disequilibrium (LD) ${r}^{2}<0.001(10000kb)$. The F statistic was used to test the strength of the association between these SNPs and the exposure factors. SNPs with strong statistical power (F statistics > 10) were included as IVs. F statistics is calculated by the formula: $\mathrm{F}={\mathrm{R}}^{2}\times (\mathrm{N}-\mathrm{K}-1)/\mathrm{K}\times (1-{\mathrm{R}}^{2})$ where R^2^ represented the variance in exposures explained by the genetic variance, K represented the number of instruments, and N meant the sample size of the GWAS for the association of the SNP-activity factors. The F-statistics of all reproductive factors were larger than 10 (Table 2), which means that the results of MR are less biased because of weak instrument [23]. Thus, it is sufficient to generate a strong genetic instrument based on the selected SNPs.

**Table 1:** Datasets

Traits	Consortium	Sample size (cases/controls)	Year	Pubmed ID
menarche	ReproGen	252,000	2014	28,436,984
Age at first sexual intercourse	-	397,338	2021	34,211,149
Age at first birth	-	542,901	2021	34,211,149
Age at last live birth	MRC-IEU	170,248	2018	-
menopause	ReproGen	201,323	2015	34,349,265
Autism spectrum disorder	PGC	46,351(18,382/27969)	2017	30,804,558
Depression	PGC	500,199(170,756/329443)	2019	30,718,901
Attention deficit or hyperactivity disorder	PGC	21,191(4945/16246)	2018	29,325,848
Obsessive compulsive disorder	PGC	9725(2688/7037)	2017	28,761,083
Schizophrenia	PGC	130,644(53,386/77258)	2022	35,396,580
Bipolar disorder (all)	PGC	413,466(41,917/371549)	2021	34,002,096
Bipolar disorder (I)	PGC	64,802(25,060/449978)	2021	34,002,096
Bipolar disorder (II)	PGC	22,560(6781/364075)	2021	34,002,096
Posttraumatic stress disorder	PGC	174,659(23,212/151447)	2019	31,594,949
Anxiety disorders	PGC	77,096(33,640/43456)	2016	26,754,954
Anorexia Nervosa	PGC	14,477(3495/10982)	2017	28,494,655
Tourette syndrome	PGC	14,307(4819/9488)	2018	30,818,990

**Table 2:** F statistics of IVs for exposures

Exposure	nSNPs	F statistics
menarche	190	67.549
menopause	198	111.759
Age at first sexual intercourse	194	46.822
Age at first birth	65	38.556
Age at last live birth	6	130.993

#### Psychiatric disorders

The GWAS summary data for psychiatric disorders were retrieved from the Psychiatric Genomes Consortium (PGC). We chose 12 related psychiatric disorders as the outcome, including anxiety disorders (33,640 cases/43,456 controls) [31], anorexia nervosa (14,477 samples) [32], attention deficit or hyperactivity disorder (4,945 cases/16,246 controls) [33], autism spectrum disorder (18,382 cases/27,969 controls) [34], bipolar disorder (41,917 cases/371,549 controls) and two BD subtypes: bipolar I disorder (25,060 cases/449,978 controls) and bipolar II disorder (6,781 cases/364,075 controls) [35], major depressive disorder (170,756 cases/329,443 controls) [36], obsessive compulsive disorder (2,688 cases/7,037 controls) [37], posttraumatic stress disorder (23,185 cases/151,309 controls) [38], schizophrenia (67,280 cases/86,912 controls) [39] and Tourette syndrome (55,386 cases/77,258 controls) [40]. Details for datasets are listed in Table 1. Ethical review and informed consent had been obtained in all of the original studies.

### Statistical analyses

#### Genetic correlation

We used high-definition likelihood (HDL) inference for genetic correlation analysis. Compared with LD score regression (LDSC), HDL makes full use of the information of the variance–covariance matrix of the Z-score in GWAS summary statistics [41]. The reference panel used in HDL is 1000 Genomes phase 3 European reference panel.

#### Univariable MR

To access the causal associations between reproductive factors and psychiatric disorders, we used “TwosampleMR” R package for MR analysis [42, 43]. The inverse variance weighted (IVW) method [23, 44] was conducted as our primary MR method to access the causal associations of reproductive behaviors in pairs, along with reproductive behaviors and psychiatric disorders. We applied a conservative Bonferroni correction (i.e.$p<0.05/70= 7.14\times {10}^{-4}$) as a strong evidence and a nominal threshold (i.e.$p<0.05$) as a weak evidence of causal relationship. We supplemented MR-Egger regression [45], weighted median [46], weighted mode [47], MR-robust adjusted profile score (MR-RAPS) [48] and MR-Mix methods [49] as our sensitivity MR analyses, which make different assumptions based on different effectiveness of the SNPs. The results from multiple methods of MR are helpful to evaluate the robustness of our MR estimations. The intercept of MR-Egger regression shows the average pleiotropic effect among all used SNPs under the InSIDE (Instrument Strength Independent of Direct Effect) assumption and a non-zero intercept means there exists directional pleiotropy. Additionally, we used applied MR-PRESSO (Mendelian Randomization Pleiotropy RESidual Sum and Outlier) [50] to identify and remove potential outliers. Egger’s test is used for pleiotropy test. Heterogeneity tests include two methods: IVW and MR-Egger.

#### Multivariable MR

Multivariable MR based on IVW method was subsequently applied in a two-sample setting. This statistical method is suitable for considering the genetically predicted effects of multiple risk factors (e.g. age at menarche and age at menopause) on an outcome (e.g. depression) simultaneously. This enables us to estimate the direct effects of a reproductive behavior in early life (i.e. the effect of age at menarche after accounting for age at menopause) and indirect effects (i.e. the effect mediated by age at menopause) on a psychiatric disorder. We applied this model using all genetic variants for the reproductive factors, which have nominal significant ($p<0.05$) causal effects on outcomes, after undertaking linkage disequilibrium clumping based on ${r}^{2}<0.001$ to ensure independence of our instruments. The statistical analyses of multivariable MR were performed using R package “MVMR” [51].

## Results

### Genetic correlation

**Fig. 1:**
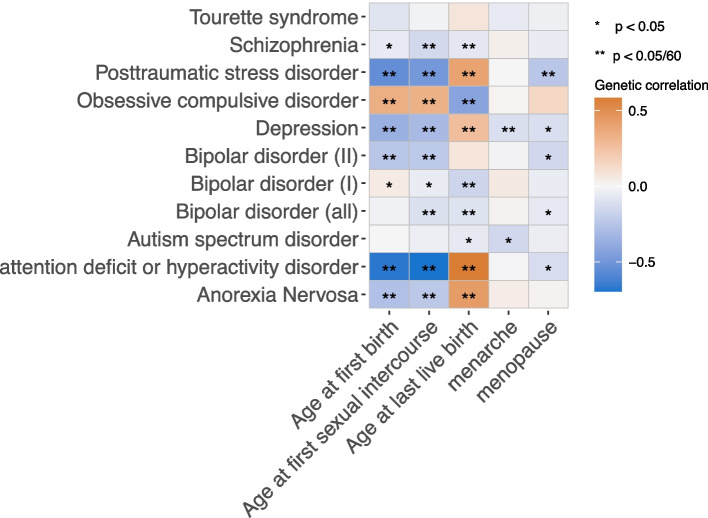
Genetic correlations between reproductive factors and psychiatric disorders

Among all the genetic correlation analyses between reproductive factors and psychiatric disorders, there were 26 pairs of traits with significant genetic correlations (${r}_{g} range:|0.07-0.69|$) under a conservative Bonferroni correction ($p<0.05/60= 8.33\times {10}^{-4}$) and another 9 pairs of traits had nominal significant genetic correlations under $p<0.05$. Results are shown in Fig. 1. Age of first sexual intercourse had the highest genetic correlations with all psychiatric disorders except autism spectrum disorder and Tourette syndrome, while age at menarche only had strong evidence of genetic correlation with depression. Among all psychiatric disorders, depression had evidence of genetic correlation with all reproductive factors (a weak evidence with menopause and strong evidence with other four traits), while there was null genetic correlation between Tourette syndrome and reproductive factors.

### Causal relationships among reproductive factors

#### Univariable MR

**Fig. 2:**
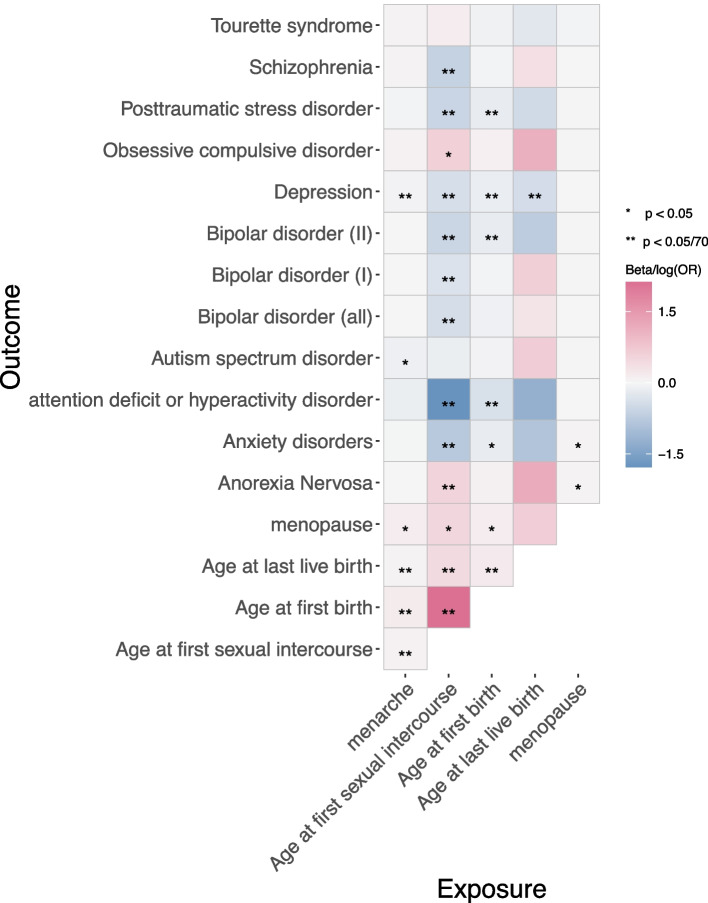
Univariable MR between reproductive factors and psychiatric disorders

Results of univariable MR among reproductive factors are shown in Fig. 2. The results of IVW method suggested that negative genetic correlation reflected causal effects of 190 SNPs determined later age at menarche (1 SD increase) on later AgeFirstSex ($\mathrm{b}=0.07;95\mathrm{\% confidence interval }(\mathrm{CI})=\mathrm{0.05,0.10}$), AgeFirstBirth ($\mathrm{b}=0.17;\mathrm{CI}=\mathrm{0.09,0.25}$) and AgeLastBirth ($\mathrm{b}=0.05;\mathrm{CI}=\mathrm{0.02,0.07}$) under Bonferroni-adjusted level of significance ($p< 7.14\times {10}^{-4}$). Results revealed that 194 SNPs determined later age at first sexual intercourse (1 SD increase) might lead to later AgeFirstBirth ($\mathrm{b}=2.11;\mathrm{CI}=\mathrm{1.96,2.27}$) and AgeLastBirth ($\mathrm{b}=0.43;\mathrm{CI}=\mathrm{0.37,0.48}$) under Bonferroni-adjusted level of significance ($p< 7.14\times {10}^{-4}$). For later age at first birth, its one SD increase led to later AgeLastBirth ($\mathrm{b}=0.19;\mathrm{CI}=\mathrm{0.17,0.20}$) under Bonferroni-adjusted level of significance ($p< 7.14\times {10}^{-4}$), using 65 SNPs as IVs. In addition, 6 SNPs determined age at last birth had no evidence of causal effect on AgeMenopause. These results were supported by weighted median, MR-RAPS methods (Figure S1) and MR PRESSO (Figure S2), suggesting the results were robust. In the results of ME PRESSO, we didn’t present the situation that outlier was not found. The rsid and r-square of IVs used in 70 univariable Mendelian randomization analyses were listed in Table S1. Egger’s test did not provide evidence that horizontal pleiotropy was driving these effects (Table S2). There is no heterogeneity in the Wald Ratio of all IVs (Table S3).

#### Multivariable MR

**Fig. 3:**
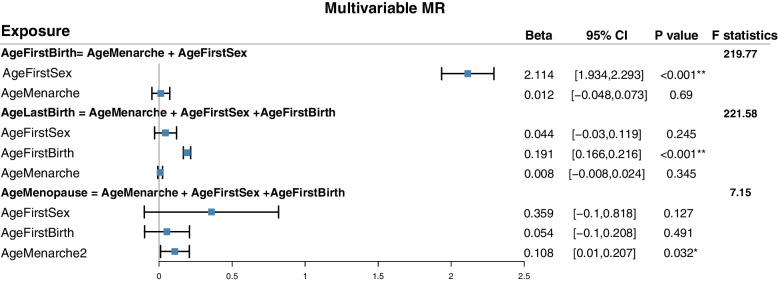
Multivariable MR among reproductive factors

Results of multivariable MR are shown in Fig. 3. Results suggested that only AgeFirstSex had significant causal effect on AgeFirstBirth ($\mathrm{b}=2.11;\mathrm{CI}=\mathrm{1.93,2.29}$), while AgeMenarche had no direct effects on AgeFirstBirth. After adjusting for AgeMenarche and AgeFirstSex, AgeFirstBirth had significant direct effect on AgeLastBirth ($\mathrm{b}=0.19;\mathrm{CI}=\mathrm{0.17,0.27}$). We also found only AgeMenarche had positive causal effect on AgeMenopause ($\mathrm{b}=0.11;\mathrm{CI}=\mathrm{0.01,0.21}$), while AgeFirstSex and AgeFirstBirth had no direct effects on AgeMenopause.

### Causal relationships between reproductive factors and psychiatric disorders

#### Age at menarche

MR findings (Fig. 2) from the primary analysis suggested that the negative genetic correlation reflects a causal relationship between later age at menarche (1 SD increase) and lower risk of depression ($\mathrm{OR}=0.96;\mathrm{CI}=\mathrm{0.93,0.98}$) under Bonferroni-adjusted level of significance ($p< 7.14\times {10}^{-4}$). Results of weighted median and MR-RAPS methods were consistent with this result (Figure S3). Egger’s test did not provide evidence of horizontal pleiotropy (Table S4). Heterogeneity existed in the Wald Ratio of age at menarche and depression and results may be affected by pleiotropy (Table S5). After removing outliers, MR PRESSO revealed that the later age at menarche reduced the risk of the depression ($\mathrm{OR}=0.96;p< 7.14\times {10}^{-4}$) (Figure S4) which means the effects remains after considering the pleiotropy.

#### Age at first sexual intercourse

Results revealed that later age at first sexual intercourse (1 SD increase) may lead to lower risk of anxiety disorder ($\mathrm{OR}=0.46;\mathrm{CI}=\mathrm{0.33,0.64}$), lower risk of ADHD ($\mathrm{OR}=0.17;\mathrm{CI}=\mathrm{0.12,0}.25$), bipolar disorder ($\mathrm{OR}=0.66;\mathrm{CI}=\mathrm{0.55,0.79}$), bipolar II disorder ($\mathrm{OR}=0.58;\mathrm{CI}=\mathrm{0.43,0.79}$), depression ($\mathrm{OR}=0.67;\mathrm{CI}=\mathrm{0.62,0.74}$), posttraumatic stress disorder ($\mathrm{OR}=0.57;\mathrm{CI}=\mathrm{0.47,0.69}$) and schizophrenia ($\mathrm{OR}=0.54;\mathrm{CI}=\mathrm{0.43,0.69}$) under Bonferroni-adjusted level of significance ($p< 7.14\times {10}^{-4}$). Although heterogeneity existed in several relationships including ADHD, bipolar disorder, bipolar II and depression (Table S5), these causal results were consistent with the results of weighted median, MR-RAPS methods (Figure S5) and MR PRESSO (Figure S6). After removing outliers of the above relationships, MR PRESSO revealed that the later age at first sexual intercourse reduced the risk of the ADHD ($\mathrm{OR}=0.16;p< 7.14\times {10}^{-4}$), bipolar disorder ($\mathrm{OR}=0.66;p< 7.14\times {10}^{-4}$), bipolar disorder II ($\mathrm{OR}=0.61;p= 0.001$) and depression ($\mathrm{OR}=0.68;p< 7.14\times {10}^{-4}$) (Figure S4) which means the effects remains after considering the pleiotropy. Egger’s test also did not provide evidence that horizontal pleiotropy (Table S4).

#### Age at first birth

We found that later age at first birth (1 SD increase) may lead to lower risk of ADHD ($\mathrm{OR}=0.69;\mathrm{CI}=\mathrm{0.60,0.79}$), depression ($\mathrm{OR}=0.87;\mathrm{CI}=\mathrm{0.84,0.90}$) and posttraumatic stress disorder ($\mathrm{OR}=0.85;\mathrm{CI}=\mathrm{0.79,0.92}$) under Bonferroni-adjusted level of significance ($p< 7.14\times {10}^{-4}$). Results of weighted median and MR-RAPS methods were consistent with these results (Figure S7). Egger’s test did not provide evidence that horizontal pleiotropy (Table S4). There existed heterogeneity in the Wald Ratio of age at first birth and depression (Table S5). The MR-PRESSO method could not remove the outliers of the relationships between AgeFirstBirth and depression, which means the result may be influenced by pleiotropy.

#### Age at last birth

MR results suggested that age at last birth had no evidence of causal effect on psychiatric disorders at Bonferroni-adjusted level of significance. Although heterogeneity existed in several relationships (Table S5), these causal results were consistent with the results of weighted median, MR-RAPS methods (Figure S9) and MR PRESSO (Figure S10). Egger’s test did not provide evidence that horizontal pleiotropy (Table S4).

#### Age at menopause

There was no evidence of causal effect of age at menopause on psychiatric disorders at Bonferroni-adjusted level of significance. Results of weighted median and MR-RAPS methods were consistent with these results (Figure S11). Egger’s test did not provide evidence that horizontal pleiotropy (Table S4).

#### Multivariable MR

**Fig. 4:**
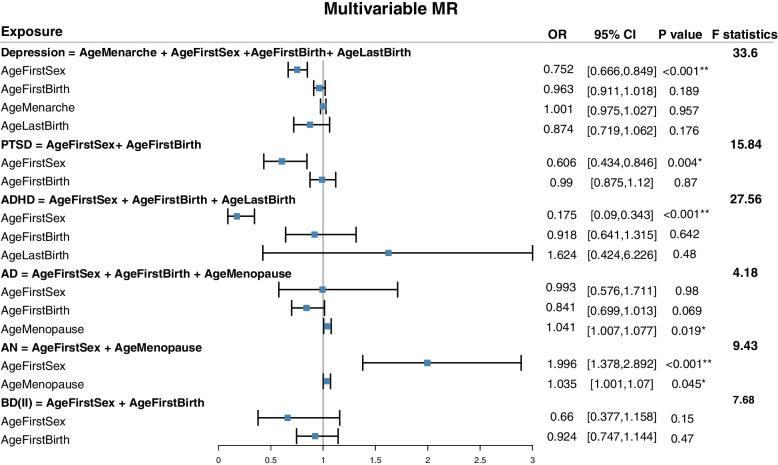
Multivariable MR between reproductive factors and psychiatric disorders

Since multiple reproductive behaviors have significant effect on the same psychiatric disorder (AgeMenarche, AgeFirstSex. AgeFirstBirth amd AgeLastBirth on depression for example), we used multivariable MR to evaluate the direct effects of these reproductive behaviors. As a result, most effects attenuating to include the null (p < 0.05) after the adjustment of multivariable MR (AgeMenarche, AgeFirstBirth and AgeLaseBirth have null effects on depression in multivariable MR for example).Results are shown in Fig. 4.

We found that only AgeFirstSex had significant causal effect on depression ($\mathrm{OR}=0.75;\mathrm{CI}=\mathrm{0.67,0.85}$), while AgeMenarche, AgeFirstBirth and AgeLastBirth had no direct effects to depression; only AgeFirstSex had significant causal effect on PTSD ($\mathrm{OR}=0.61;\mathrm{CI}=\mathrm{0.43,0.85}$), while AgeFirstBirth had no direct effect; only AgeFirstSex had significant causal effect on ADHD ($\mathrm{OR}=0.21;\mathrm{CI}=\mathrm{0.10,0.41}$), while AgeMenarche and AgeFirstBirth had no direct effects; only AgeMenopause had significant causal effect on anxiety disorders ($\mathrm{OR}=1.04;\mathrm{CI}=\mathrm{1.00,1.08}$), while AgeFirstSex and AgeFirstBirth had no direct effects; AgeFirstSex and AgeMenopause both had significant direct effects to anorexia nervosa ($\mathrm{OR}=2.00;\mathrm{CI}=\mathrm{1.38,2.90}$ for AgeFirstSex and $\mathrm{OR}=1.04;\mathrm{CI}=\mathrm{1.00,1.07}$ for AgeMenopause); neither reproductive factor had significant causal effect on bipolar II disorder.

**Fig. 5:**
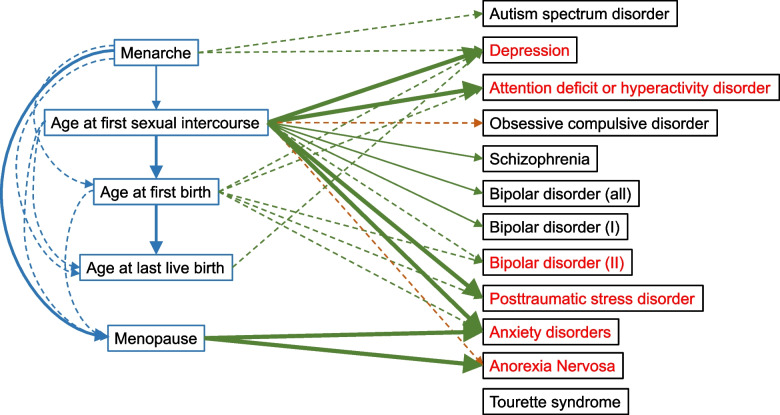
Causal relationships between reproductive factors and psychiatric disorders. The dashed lines represent the significant results in univariable MR but null significant results in multivariable MR or nominal significant results in univariable MR. The solid lines represent the significant results in both univariable and multivariable MR. The green lines represent the negative effects. The brown and blue lines represent the positive effects. Psychiatric disorders with red color represent the diseases considered in multivariable MR analysis

This indicates that evidence of the total effects between AgeMenarche and depression detected in the univariable analysis may due to the long-term effects of the earlier reproductive behavior across the life course (i.e. not just age at menarche). The evidence of the effects between AgeFirstBirth and depression, PTSD, ADHD and anxiety disorder were likely confounded by AgeFirstSex, suggesting there was no causal relations between AgeFirstBirth and these psychiatric disorders. The evidence of the effects between AgeLastBirth and depression, ADHD and anxiety disorder were likely also confounded by AgeFirstSex, suggesting there was no causal relations between AgeFirstBirth and these psychiatric disorders. Figure 5 is the causal graph that shows where we found evidence of an effect between reproductive behaviors and psychiatric disorders.

## Discussion

We present evidence suggesting causal effects of several female reproductive behaviors, including AgeMenarche, AgeFirstSex and AgeFirstBirth, on psychiatric disorders using MR analyses. However, these effects estimation attenuated once accounting for other reproductive behaviors of different stages of life using multivariable MR analyses. These causal association between reproductive behaviors in early life and later psychiatric disorders may be explained by the sustained impact of earlier sexual maturity in childhood and thus tend to remain so into adulthood. On the other hand, impact of common cause (i.e. AgeFirstSex) is also important, and it has causal effects on both reproductive factors during later adulthood and psychiatric disorders.

We corroborate the direct effects of age at menarche on AgeFirstSex and age at menopause in previous studies [18, 19, 52, 53], while showing the effects of age at menarche on AgeFirstBirth and AgeLastBirth are totally mediated by AgeFirstSex and AgeFirstBirth, respectively. Our study also supports positive causal links between AgeFirstSex and AgeFirstBirth, AgeFirstBirth and AgeLastBirth [21, 53, 54]. We show that earlier reproductive behaviors including age at menarche, AgeFirstSex and AgeFirstBirth have effects on subsequent reproductive events through two main pathways: (i) the directed path from AgeMenarche to AgeMenopause; (ii) the pathway from age at menarche to AgeLastBirth through AgeFirstSex and AgeFirstBirth. Our findings suggest that for reproductive behaviors except age at menopause, an earlier reproductive behavior only have direct causal effect on the next reproductive behavior following the temporal order; and the age at menopause only affected by age at menarche. These findings oppose previous MR studies that supported causal effects between AgeFirstSex and age at menopause or AgeFirstBirth and age at menopause [53, 55–59].

For the causal relations between reproductive behaviors and psychiatric disorders, our study supports evidence for the earlier of age of reproductive behaviors in early life are causal factors in the rising risks of psychiatric disorders in previous studies [4–17]. However, the null effects of age at menarche, AgeFirstBirth and AgeLastBirth after accounting for AgeFirstSex using multivariable MR suggest that the effect of age at menarche on depression is totally mediated by AgeFirstSex and the effects of AgeFirstBirth and AgeLastBirth on psychiatric disorders are confounded by AgeFirstSex. Our findings show that AgeFirstSex is a direct risk factor on the pathway from reproductive behaviors to multiple psychiatric disorders, which has sustained impact on both reproductive behaviors and psychiatric disorders. The multivariable MR estimates for reproductive behaviors illustrate the importance of using a lifecourse MR approach to separate the effects of reproductive behaviors at separate stages in the life course [60–62].

One explanation for why earlier reproductive behaviors lead to earlier subsequent reproductive behaviors and psychiatric disorders is the life history theory. The core idea of this theory is to allocate limited resources between reproductive efforts and human growth, which can be divided into ‘fast’ and ‘slow’ life history strategies [63, 64]. Our study suggests that earlier AgeMenarche has subsequent causal effects on other reproductive behaviors using multivariable MR analyses, e.g. earlier AgeFirstSex [65] and AgeMenopause, which is supported by ‘fast’ life history strategy. A ‘fast’ life history strategy makes more direct efforts to reproduce, such as earlier sexual intercourse which may lead to an earlier AgeFirstBirth [63, 64] and completeness of reproduction at a younger age which lead to earlier AgeMenopause [66, 67]. Besides the age of reproductive behavior, life history theory can also explain the association between reproductive behaviors and psychiatric disorders. Our results of multivariable MR analyses suggest that earlier AgeFirstSex is an important risk factor for several psychiatric disorders including depression, PTSD etc. The ‘fast’ strategy in modern environments of earlier AgeFirstSex is associated with teenage pregnancy and risk behaviors like violence, criminality, and substance abuse because of the little knowledge of reproductive health and these risk behaviors have been shown to be risk factors of multiple psychiatric disorders [68–73]. Our findings suggest that for preventing psychiatric disorders, it is important to increase sex education to help young individuals protect themselves from poor living conditions including sexual abuse or strong peer pressure, and it is also important to emphasize the protection and communication from parents.

Our research has various strengths and limitations. Firstly, we used data from large meta-analyzed sample of PGC consortia and ReproGen consortium, whose sample size is larger than any other studies before. On the other hand, the MR framework allowed us to avoid the effects of unmeasured confounders and reverse causation compared to more traditional epidemiology approaches [74] and the sensitive analysis of MR allowed us to evaluate the strong assumptions of MR. Furthermore, we used multivariable MR to investigate the causal pathways from five reproductive behaviors to twelve psychiatric disorders, which shows whether women with earlier menarche and first sexual intercourse could reduce the risk of psychiatric disorders by attaining a different sexual lifestyle in later life. Conversely, one of the main limitations of our study is that all participants were of European descent. Therefore, it is not clear whether our findings are applicable to other ethnicities or populations of different ages, and further study needs to be discussed using samples from other ancestry groups or age groups [75–78]. In addition, the GWAS of the age of reproductive behaviors were derived from recall data with the possibility of recall bias, especially for reproductive behaviors during early life such as age at menarche and age at first sexual intercourse [75, 76]. Another limitation of our study is that the ages of some important reproductive events are not included in our study such as the age of some reproductive diseases or the age at abortion. The second assumption (Exchangeability) is difficult to test in application due to the complex nature of phenotypes and their potential to be directly affected by the IVs used here, e.g. rs113247159 and rs359271 are selected as IVs for both age at first birth and age at first sexual intercourse. Figure 2 shows that these two reproductive traits are both causally related to three psychiatric disorders: posttraumatic stress disorder, depression and attention deficit or hyperactivity disorder at a Bonferroni significance level (0.05/70). In Fig. 2, we also find that age at first sexual intercourse affects age at first birth. In this situation, age at first sexual intercourse may be the confounder between age at first birth and each psychiatric disorder, but the IVs of age at first birth are associated with this confounder, this phenomenon violates the Exchangeability assumption. Next, we perform MVMR for these three psychiatric disorders to explore the independent causal effect of two reproductive traits on each psychiatric disorder. Thus, to some extent, MVMR avoids the violation of the Exchangeability assumption. However, there are inevitable and unknown mechanisms that may violate this assumption and we cannot capture, e.g. these three psychiatric disorders may have causal relationships with each other [79, 80], the IVs may affect the interested psychiatric disorder not through the exposure but through the other psychiatric disorder. This is a limitation for MR studies. For the no pleiotropic effects assumption, we used MR Egger intercept to show no evidence of balanced pleiotropy. However, the results of heterogeneity test showed there may be directional pleiotropic. Though MR-PRESSO results showed that most effects remained significantly after removing the outliers, pleiotropic may still have potential impact on the analyses in some situations such as the violation of the InSIDE condition which is untestable. False positive is another limitation of our study, since we provided tens of univariable Mendelian randomization analysis using the IVW method. The associations in the previous sections are under a Bonferroni-adjusted level of significance ($p< 7.14\times {10}^{-4}$). However, only few results of sensitivity analyses could get the conserved Bonferroni significance. There may still be possibility of false positives of our results. The results still need to be verified by longitudinal cohort studies in future studies.

## Conclusion

In conclusion, our findings provided evidence of causal relationships between age at first sexual intercourse and five psychiatric disorders and emphasis the importance of implementing preventative polices to control the age at first sexual intercourse in young age and its subsequent influence on the rising numbers of psychiatric disorders.

### Supplementary Information

**Additional file 1: Table S1.** Instrumental variables in MR analyses. **Table S2.** Egger’s test of reproductive factors. **Table S3.** Heterogeneity test of reproductive factors. **Table S4.** Egger’s test of reproductive factors on psychiatric diseases. **Table S5.** Heterogeneity test of reproductive factors on psychiatric diseases. **Table S6.** Values for Fig. 2.

**Additional file 2:** **Figure S1.** MR results of reproductive factors. **Figure S2.** MR PRESSO results of reproductive factors. **Figure S3.** MR results of age at menarche on psychiatric diseases. **Figure S4.** MR PRESSO results of age at menarche on psychiatric diseases. **Figure S5.** MR results of age at first sexual intercourse on psychiatric diseases. **Figure S6.** MR PRESSO results of age at first sexual intercourse on psychiatric diseases. **Figure S7.** MR results of age at first birth on psychiatric diseases. **Figure S8.** MR PRESSO results of age at first birth on psychiatric diseases. **Figure S9.** MR results of age at last live birth on psychiatric diseases. **Figure S10.** MR PRESSO results of age at last live birth on psychiatric diseases. **Figure S11.** MR results of age at menopause on psychiatric diseases. **Figure S12.** MR PRESSO results of age at menopause on psychiatric diseases. **Figure S13.** Regression scatter plots for MR analysis.

## Data Availability

All the GWAS summary data are publicly available. GWAS summary data for reproductive behaviors can be download at MR base (https://www.mrbase.org/). GWAS summary data for psychiatric disorders can be download at PGC consortium (https://pgc.unc.edu/).
