# Supplementary material for: Causal associations between female reproductive behaviors and psychiatric disorders: a lifecourse Mendelian randomization study
Source: BMC Psychiatry. 2023 Nov 2;23:799. doi: 10.1186/s12888-023-05203-y (PMC10621101; doi:10.1186/s12888-023-05203-y)
Supplement: Supplementary file 2 — Additional file 2: Figure S1. MR results of reproductive factors. Figure S2. MR PRESSO results of reproductive factors. Figure S3. MR results of age at menarche on psychiatric diseases. Figure S4. MR PRESSO results of age at menarche on psychiatric diseases. Figure S5. MR results of age at first sexual intercourse on psychiatric diseases. Figure S6. MR PRESSO results of age at first sexual intercourse on psychiatric diseases. Figure S7. MR results of age at first birth on psychiatric diseases. Figure S8. MR PRESSO results of age at first birth on psychiatric diseases. Figure S9. MR results of age at last live birth on psychiatric diseases. Figure S10. MR PRESSO results of age at last live birth on psychiatric diseases. Figure S11. MR results of age at menopause on psychiatric diseases. Figure S12. MR PRESSO results of age at menopause on psychiatric diseases. Figure S13. Regression scatter plots for MR analysis. [file 12888_2023_5203_MOESM2_ESM.pdf]

# Supplemental Figures

## Content


Figure S1. MR results of reproductive factors ........ 2
Figure S2. MR PRESSO results of reproductive factors ........ 3
Figure S3. MR results of age at menarche on psychiatric diseases ........ 4
Figure S4. MR PRESSO results of age at menarche on psychiatric diseases ........ 5
Figure S5. MR results of age at first sexual intercourse on psychiatric diseases ........ 6
Figure S6. MR PRESSO results of age at first sexual intercourse on psychiatric diseases ........ 7
Figure S7. MR results of age at first birth on psychiatric diseases ........ 8
Figure S8. MR PRESSO results of age at first birth on psychiatric diseases ........ 9
Figure S9. MR results of age at last live birth on psychiatric diseases ........ 10
Figure S10. MR PRESSO results of age at last live birth on psychiatric diseases ........ 11
Figure S11. MR results of age at menopause on psychiatric diseases ........ 12
Figure S12. MR PRESSO results of age at menopause on psychiatric diseases ........ 13
Figure S13. Regression scatter plots for MR analysis ........ 43

Table S1. Instrumental variables in MR analyses
Table S2. Egger's test of reproductive factors
Table S3. Heterogeneity test of reproductive factors
Table S4. Egger's test of reproductive factors on psychiatric diseases
Table S5. Heterogeneity test of reproductive factors on psychiatric diseases
Table S6. Values for Figure 2

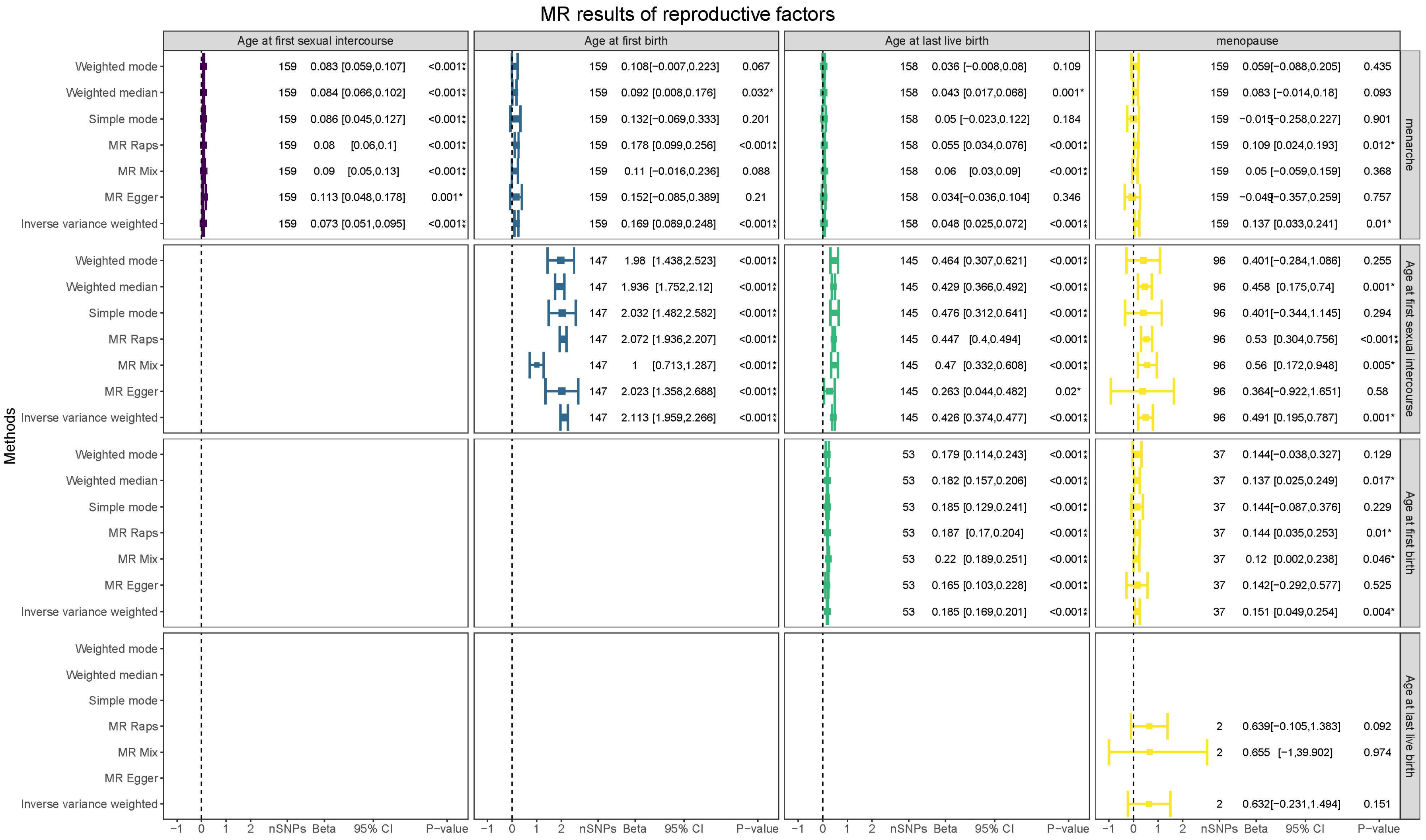


**Figure S1. MR results of reproductive factors**

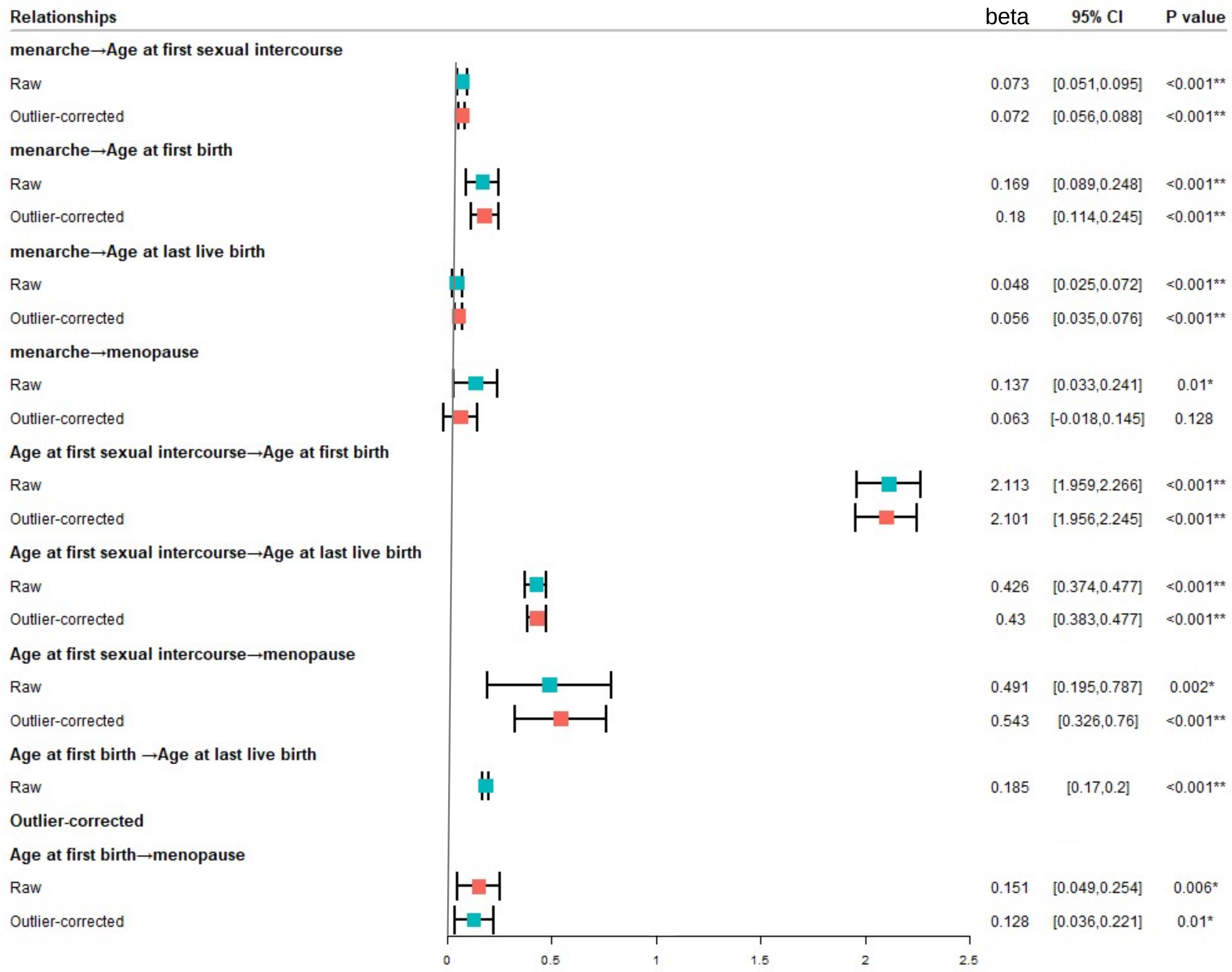


**Figure S2. MR PRESSO results of reproductive factors**

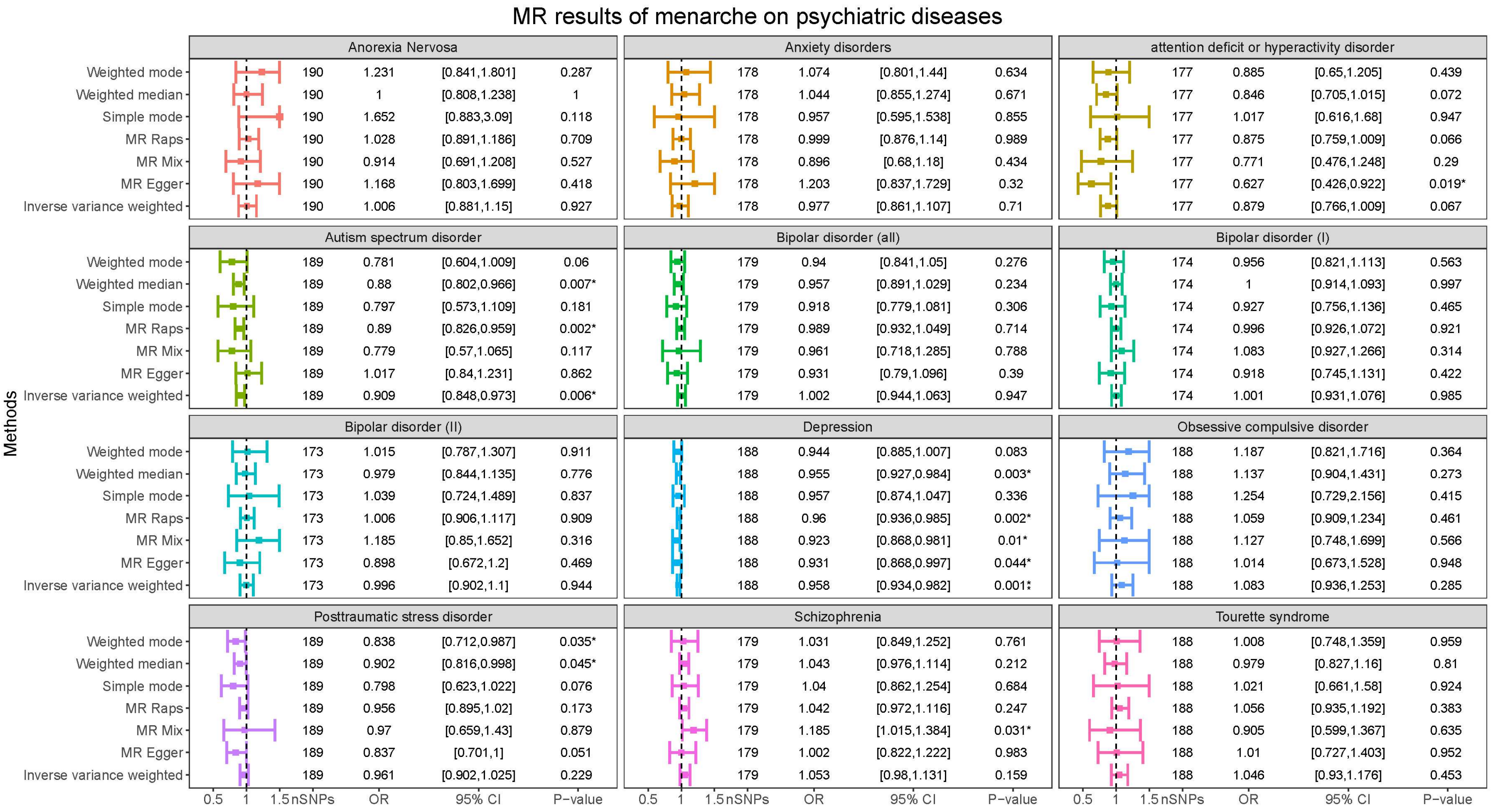


**Figure S3. MR results of age at menarche on psychiatric diseases**

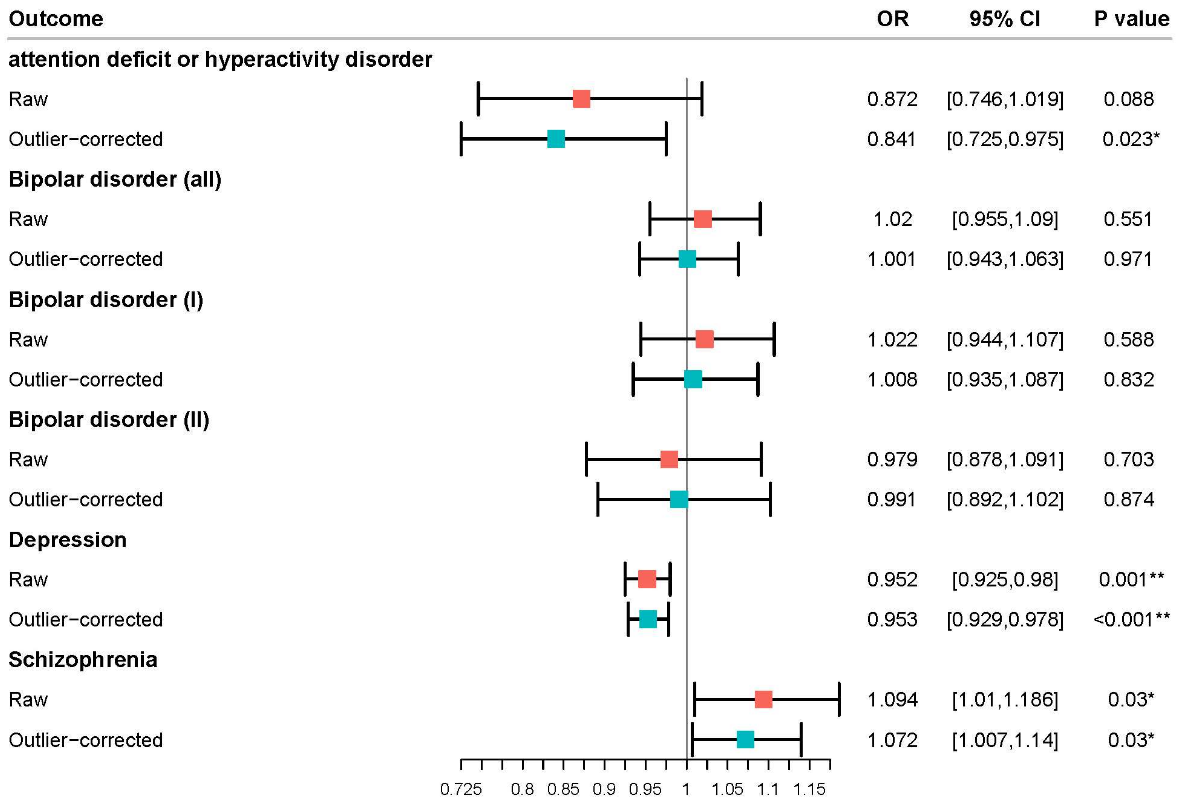


**Figure S4. MR PRESSO results of age at menarche on psychiatric diseases**

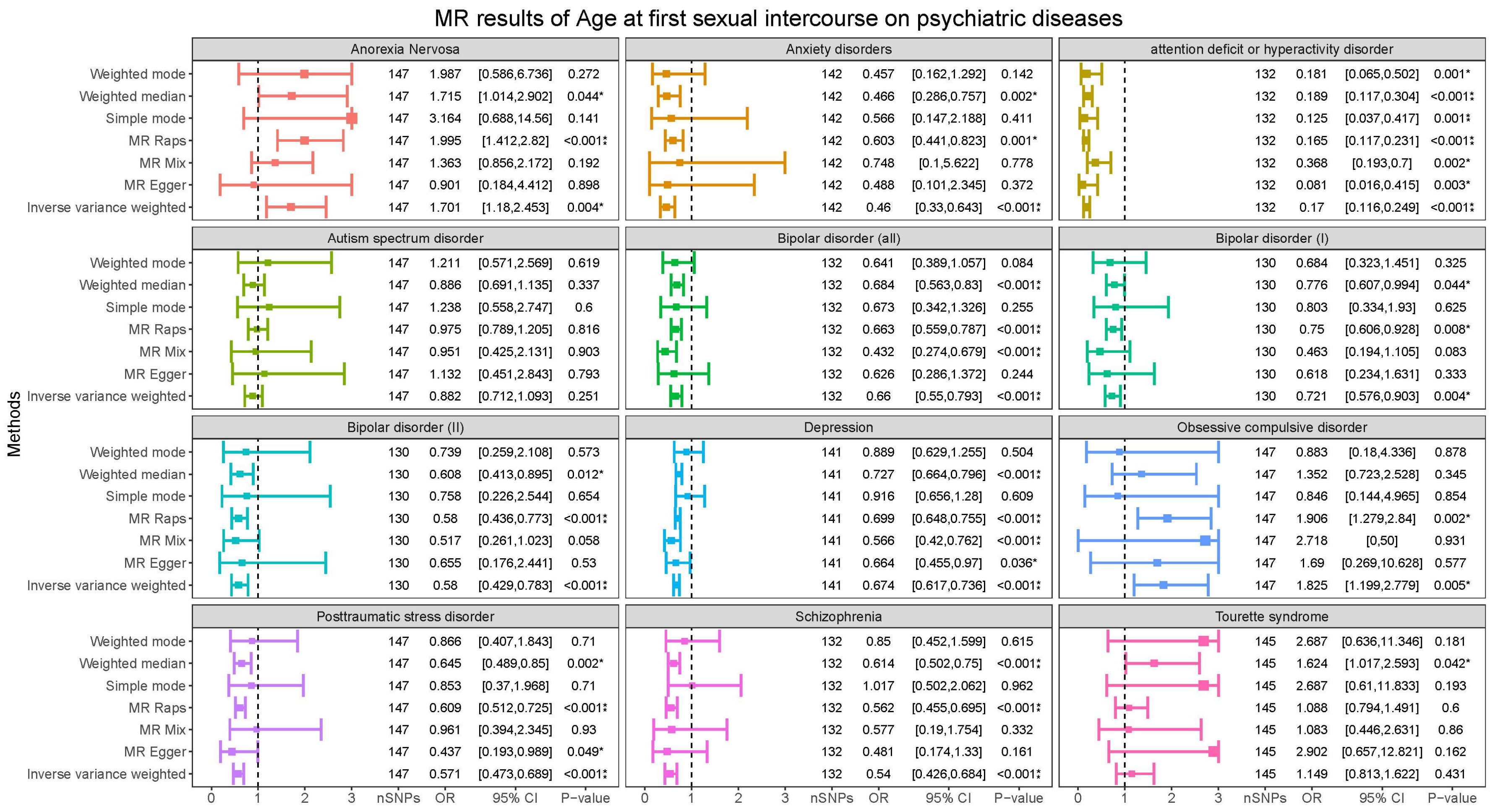


**Figure S5. MR results of age at first sexual intercourse on psychiatric diseases**

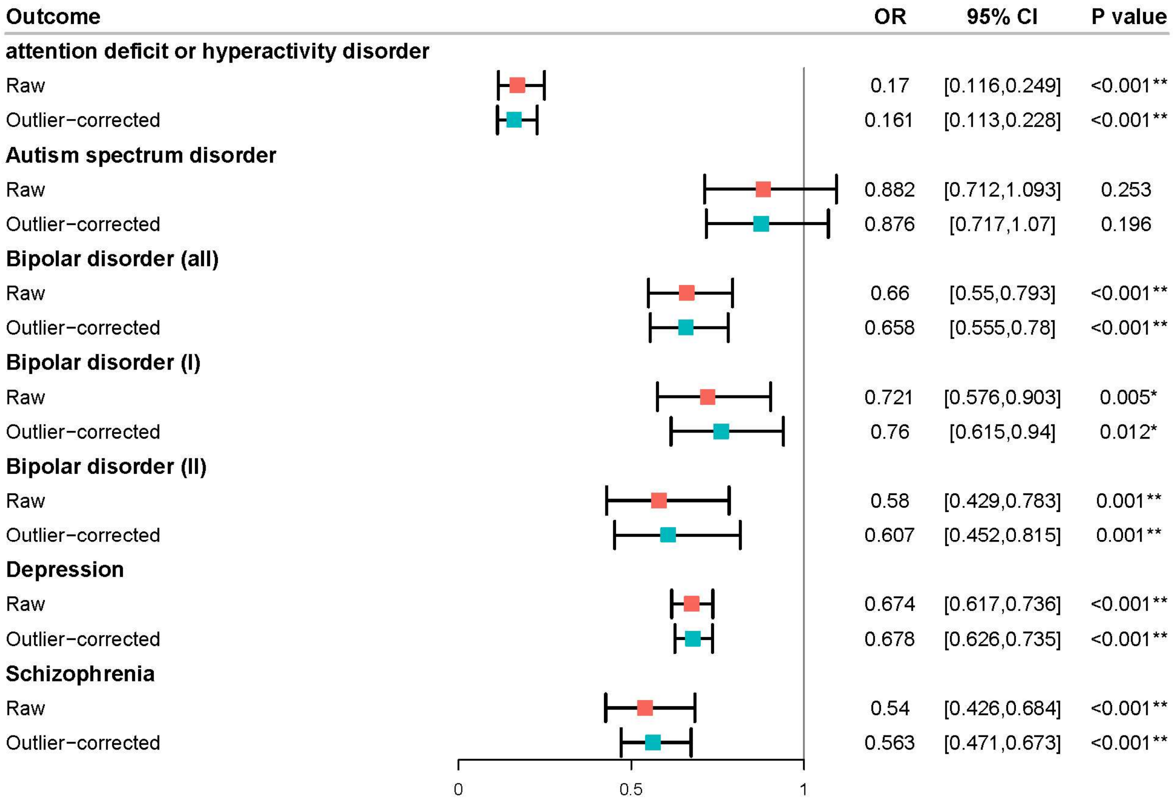


**Figure S6. MR PRESSO results of age at first sexual intercourse on psychiatric diseases**

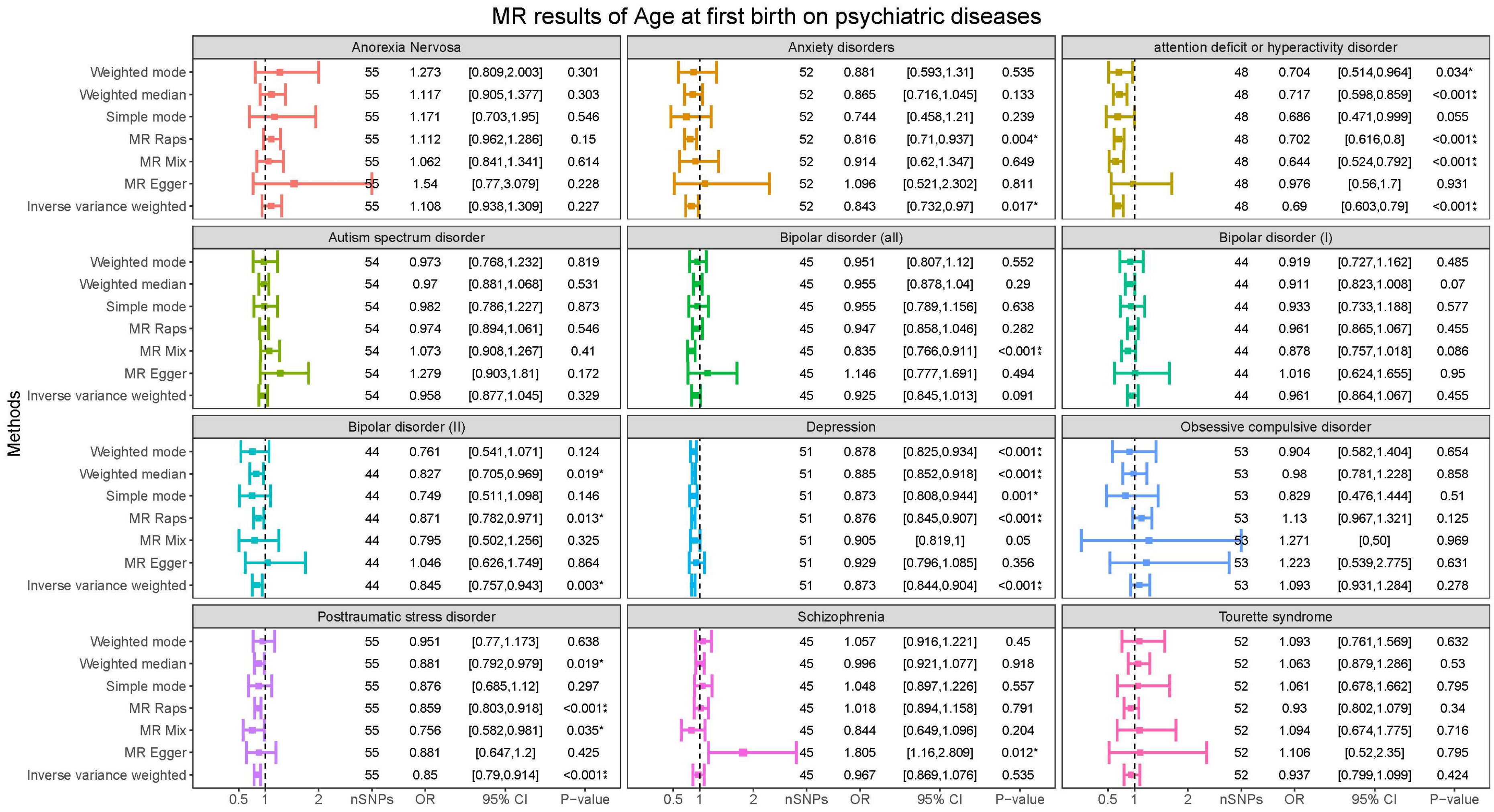


**Figure S7. MR results of age at first birth on psychiatric diseases**

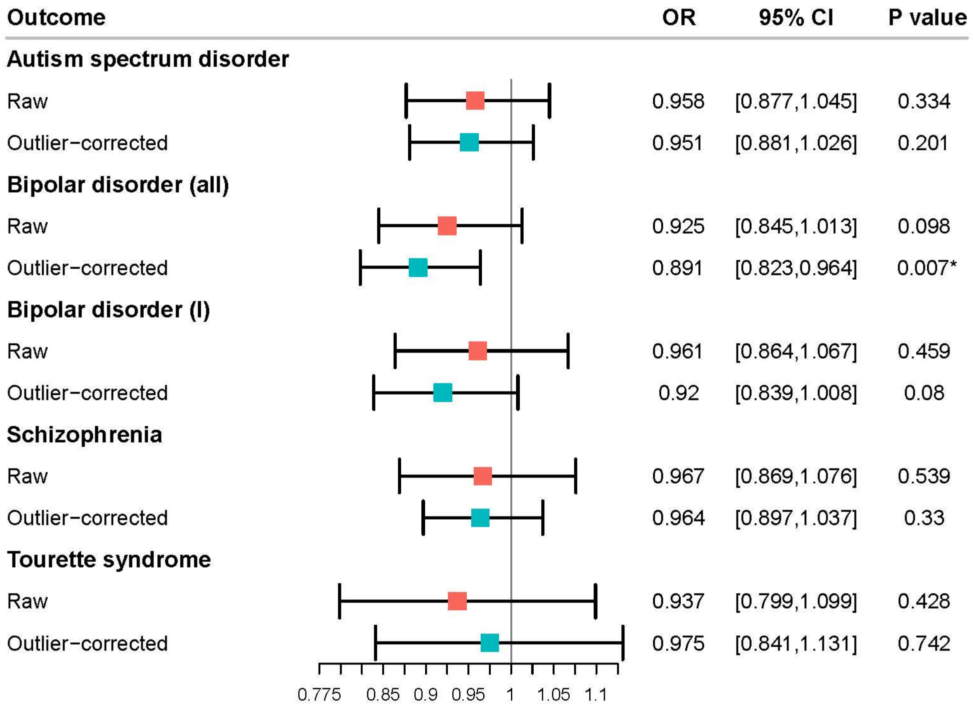


**Figure S8. MR PRESSO results of age at first birth on psychiatric diseases**

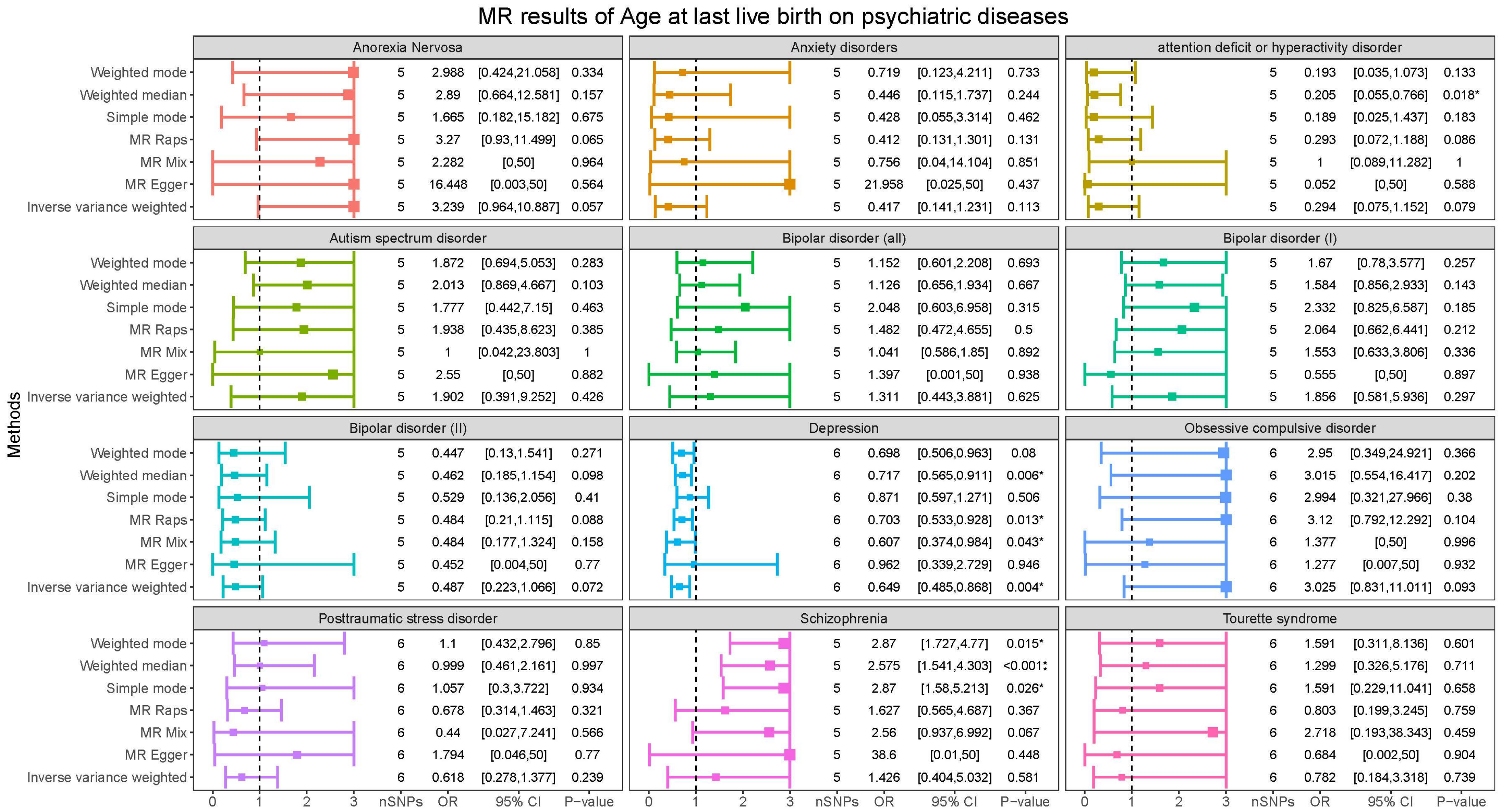


**Figure S9. MR results of age at last live birth on psychiatric diseases**

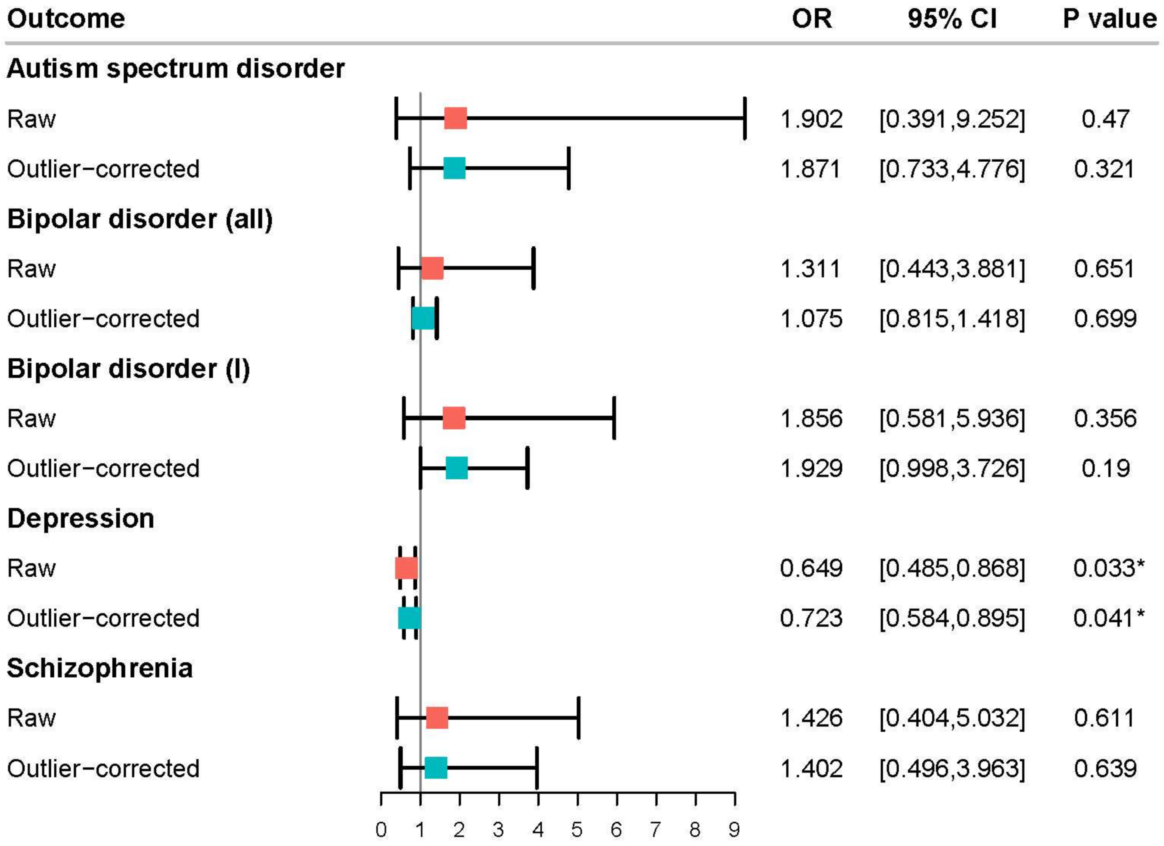


**Figure S10. MR PRESSO results of age at last live birth on psychiatric diseases**

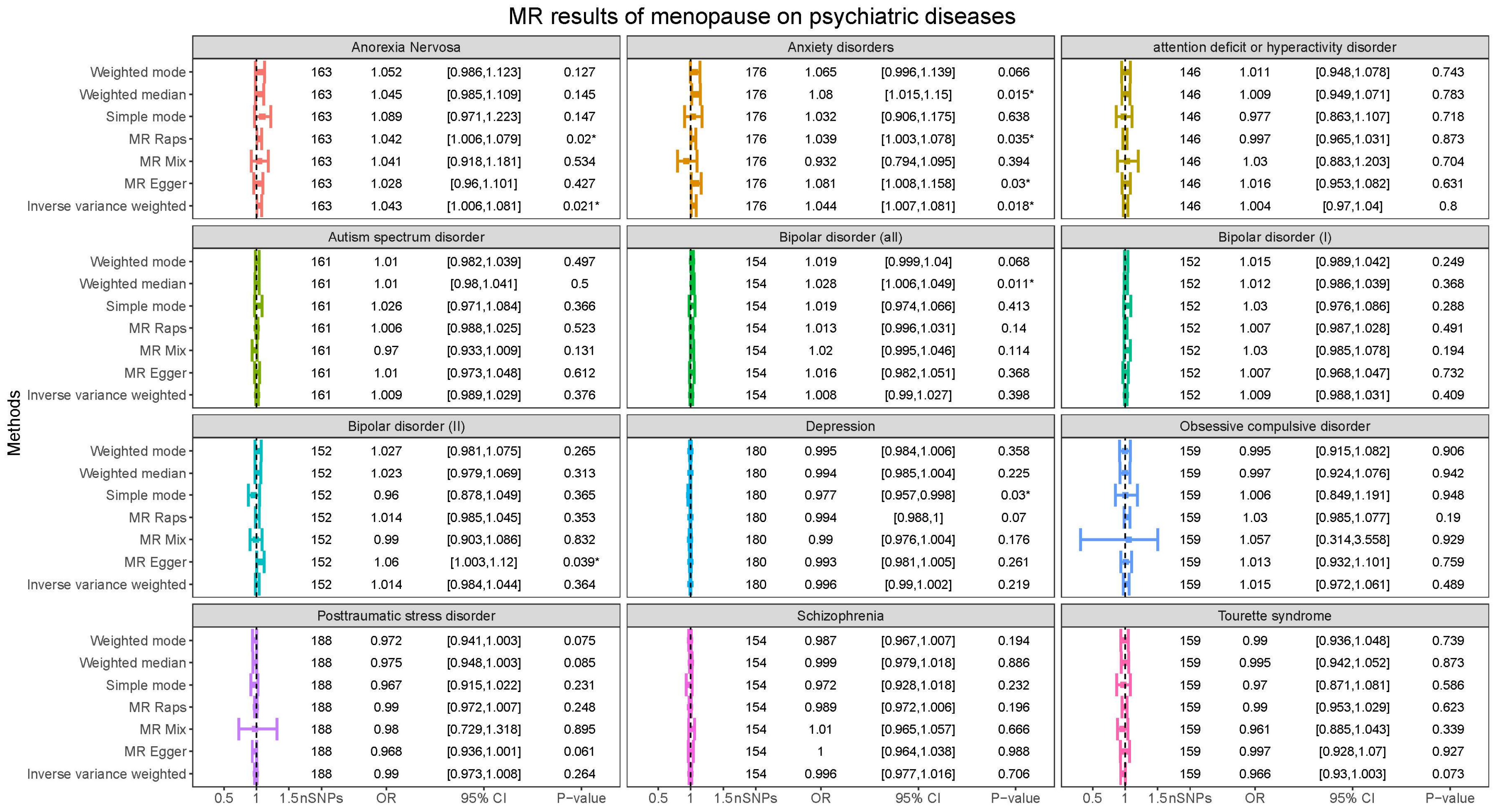


**Figure S11. MR results of age at menopause on psychiatric diseases**

| Outcome | OR | 95% CI | P value |
|---|---|---|---|
| **Autism spectrum disorder** | | | |
| Raw | 1.009 | [0.989,1.029] | 0.378 |
| Outlier−corrected | 1.01 | [0.991,1.028] | 0.31 |
| **Bipolar disorder (all)** | | | |
| Raw | 1.008 | [0.99,1.027] | 0.399 |
| Outlier−corrected | 1.017 | [1.002,1.033] | 0.032* |
| **Bipolar disorder (I)** | | | |
| Raw | 1.009 | [0.988,1.031] | 0.41 |
| Outlier−corrected | 1.013 | [0.993,1.033] | 0.212 |
| **Bipolar disorder (II)** | | | |
| Raw | 1.014 | [0.984,1.044] | 0.366 |
| Outlier−corrected | 1.018 | [0.99,1.047] | 0.216 |
| **Depression** | | | |
| Raw | 0.996 | [0.99,1.002] | 0.22 |
| Outlier−corrected | 0.994 | [0.988,1] | 0.072 |
| **Schizophrenia** | | | |
| Raw | 0.996 | [0.977,1.016] | 0.706 |
| Outlier−corrected | 0.987 | [0.97,1.003] | 0.116 |
| **Tourette syndrome** | | | |
| Raw | 0.966 | [0.93,1.003] | 0.075 |
| Outlier−corrected | 0.969 | [0.934,1.005] | 0.091 |

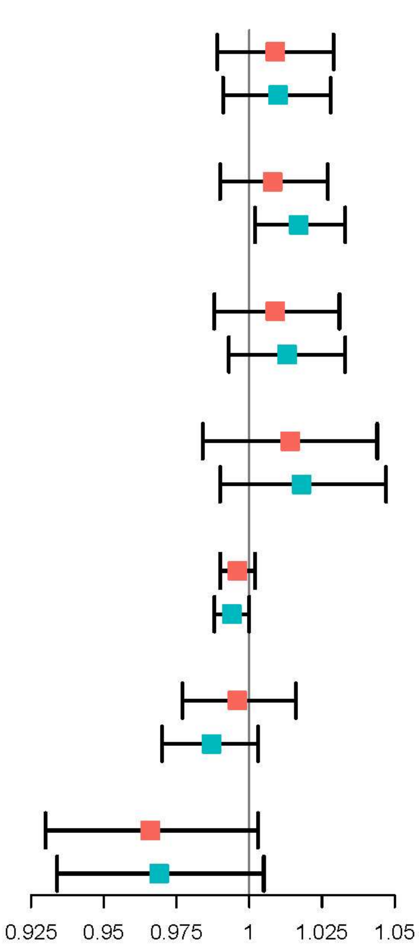


**Figure S12. MR PRESSO results of age at menopause on psychiatric diseases**

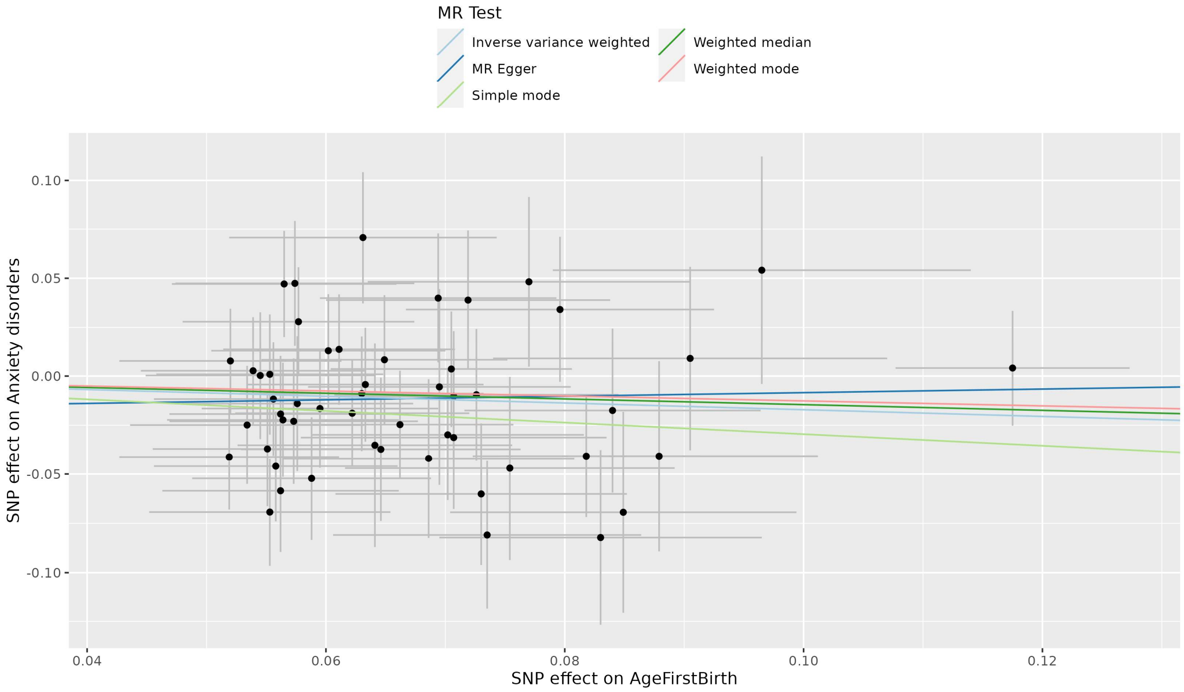


Scatter plot of AgeFirstBirth on Anxiety disorders

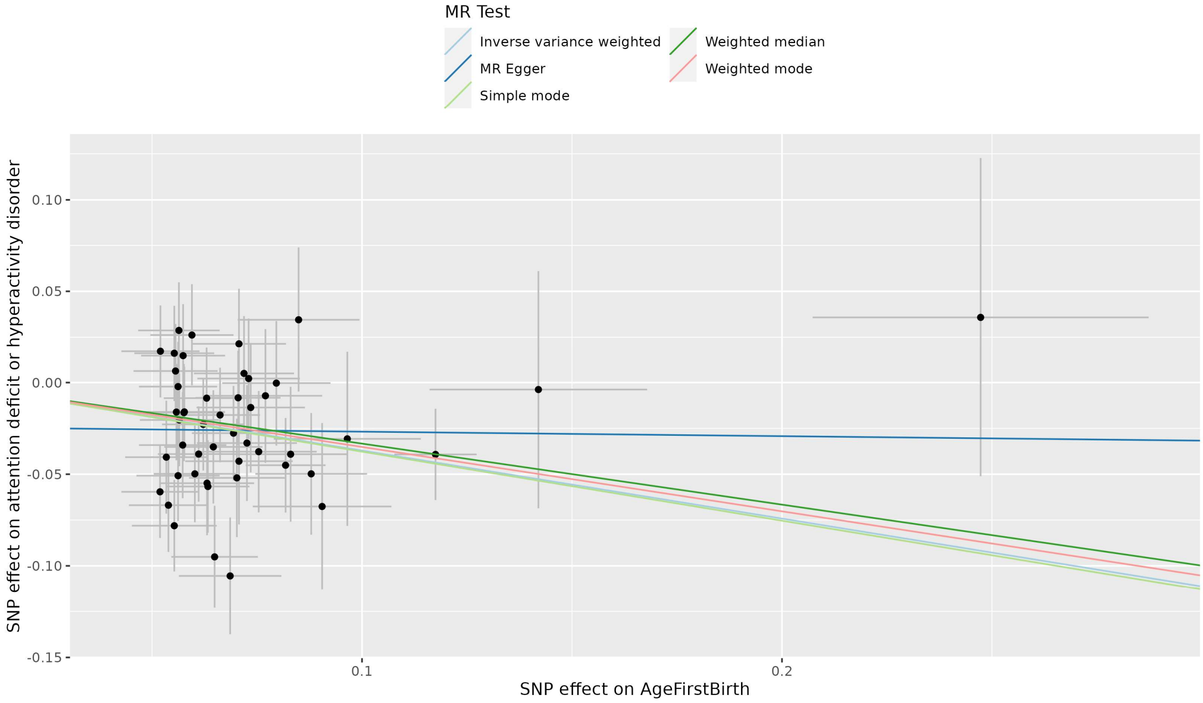


Scatter plot of AgeFirstBirth on attention deficit or hyperactivity disorder

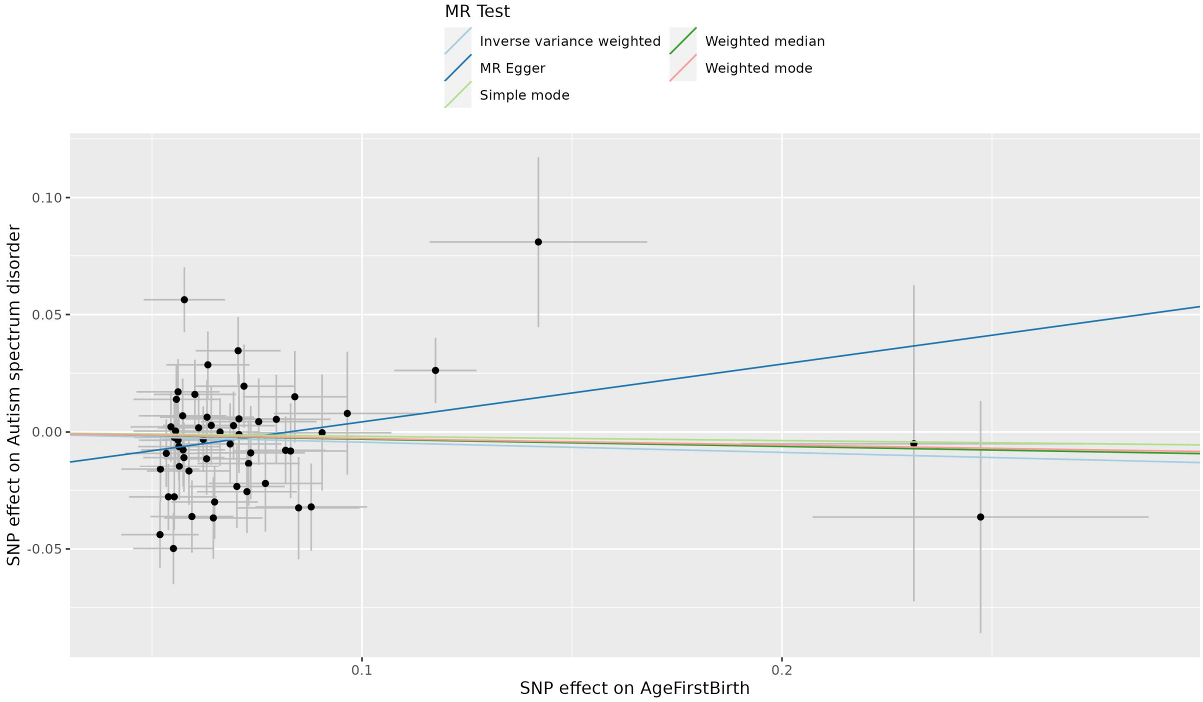


Scatter plot of AgeFirstBirth on Autism spectrum disorder

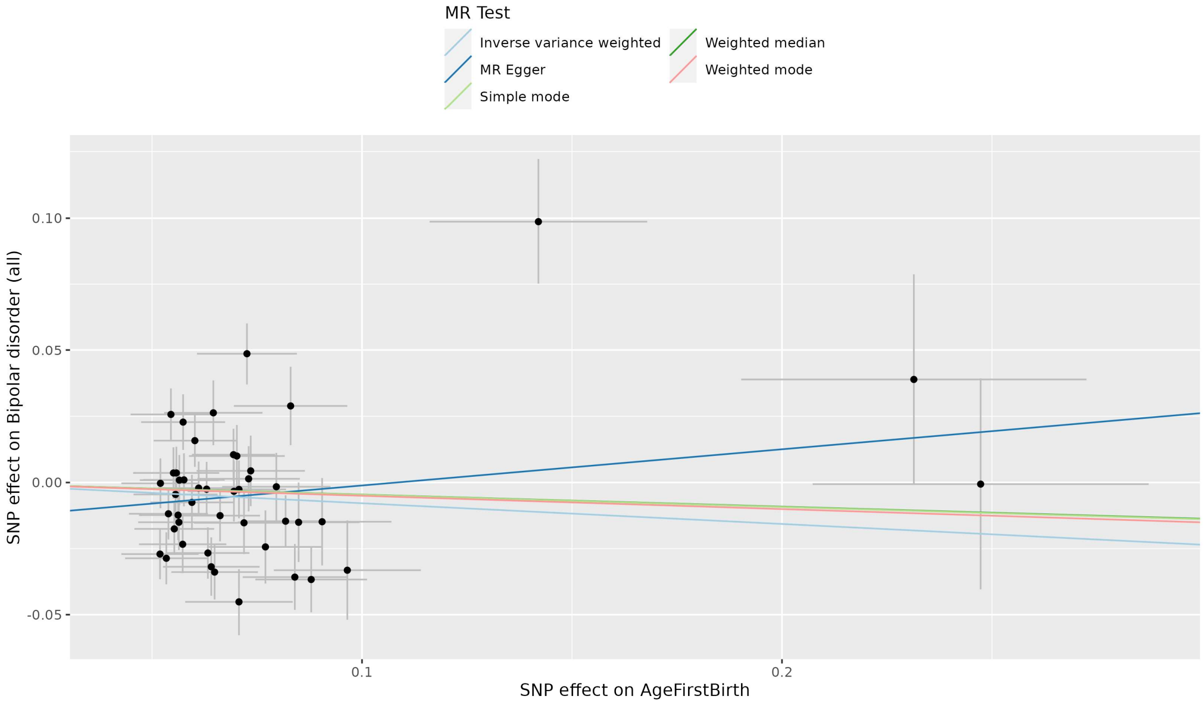


Scatter plot of AgeFirstBirth on Bipolar disorder (all)

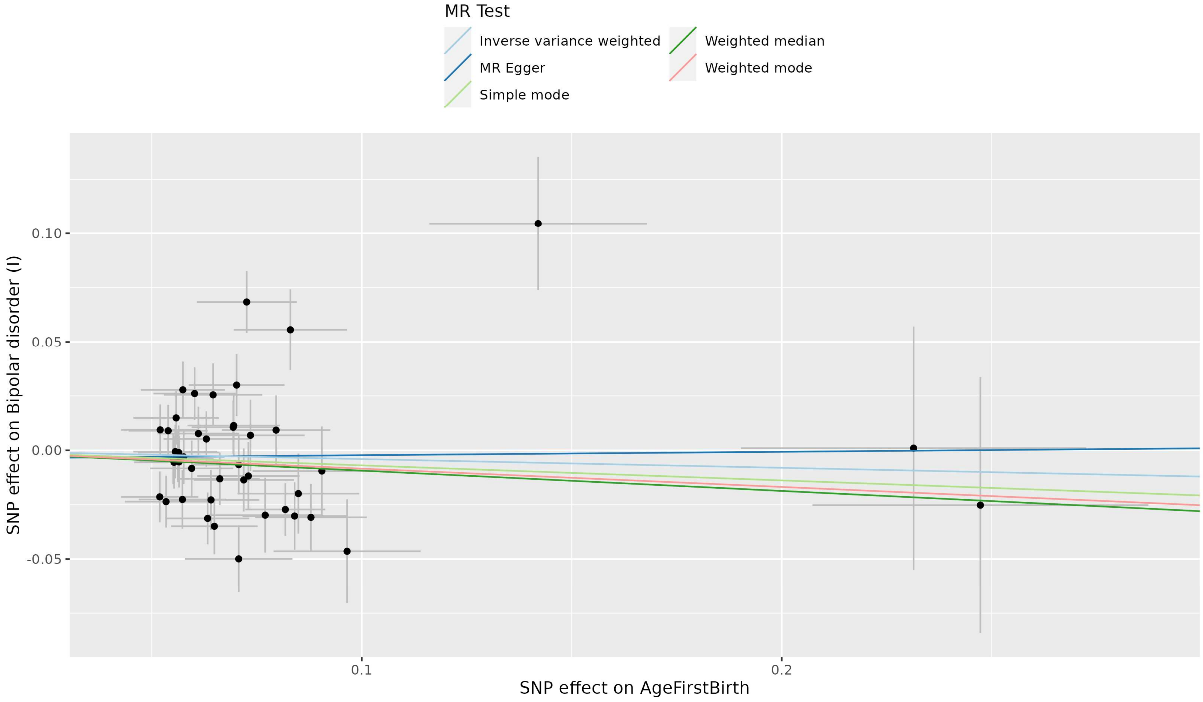


Scatter plot of AgeFirstBirth on Bipolar disorder (I)

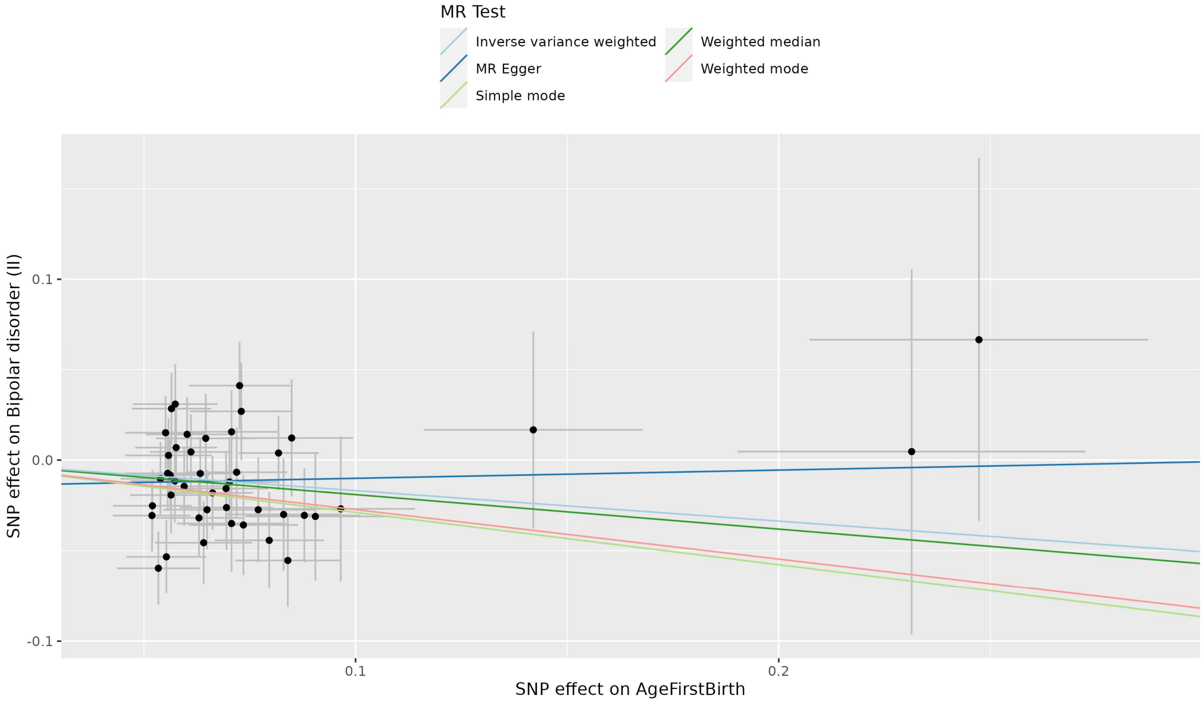


Scatter plot of AgeFirstBirth on Bipolar disorder (II)

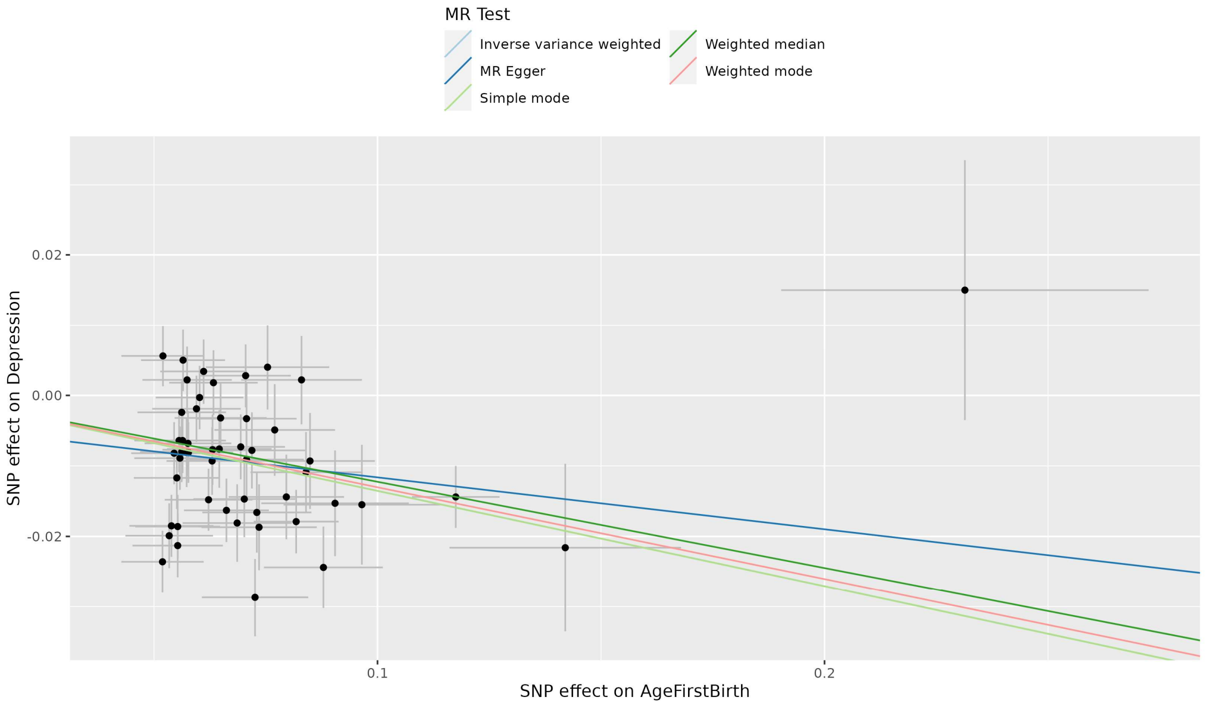


Scatter plot of AgeFirstBirth on Depression

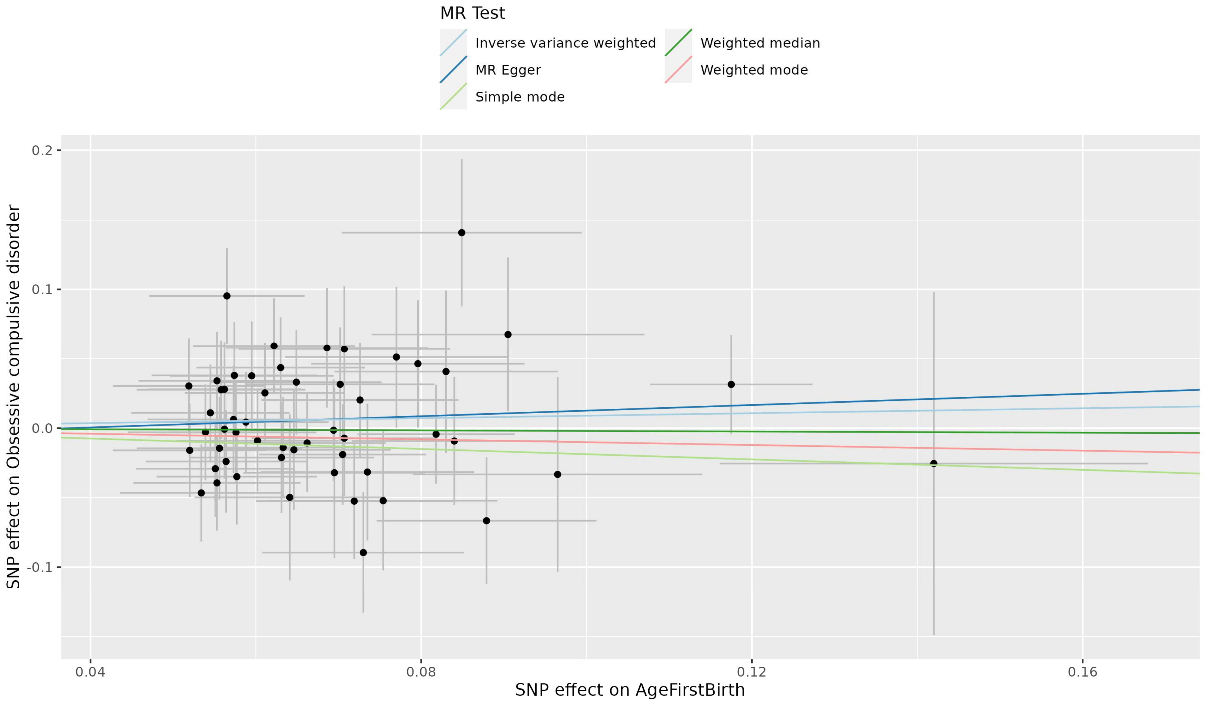


Scatter plot of AgeFirstBirth on Obsessive compulsive disorder

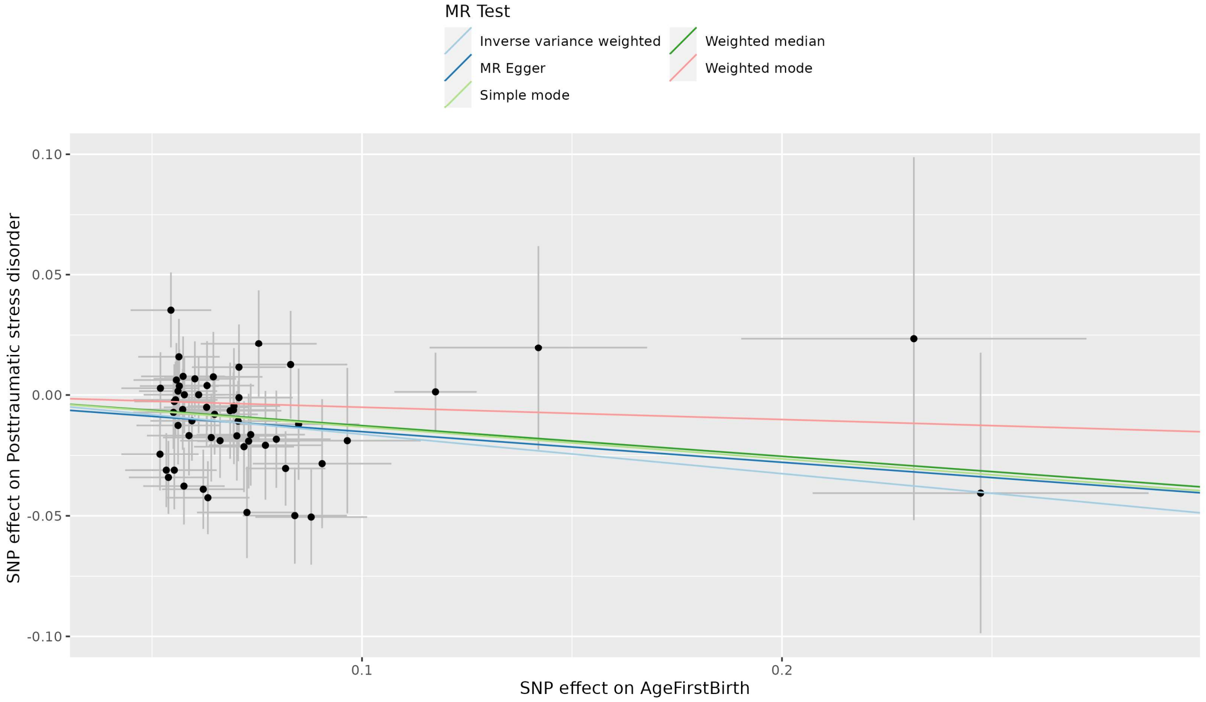


Scatter plot of AgeFirstBirth on Posttraumatic stress disorder

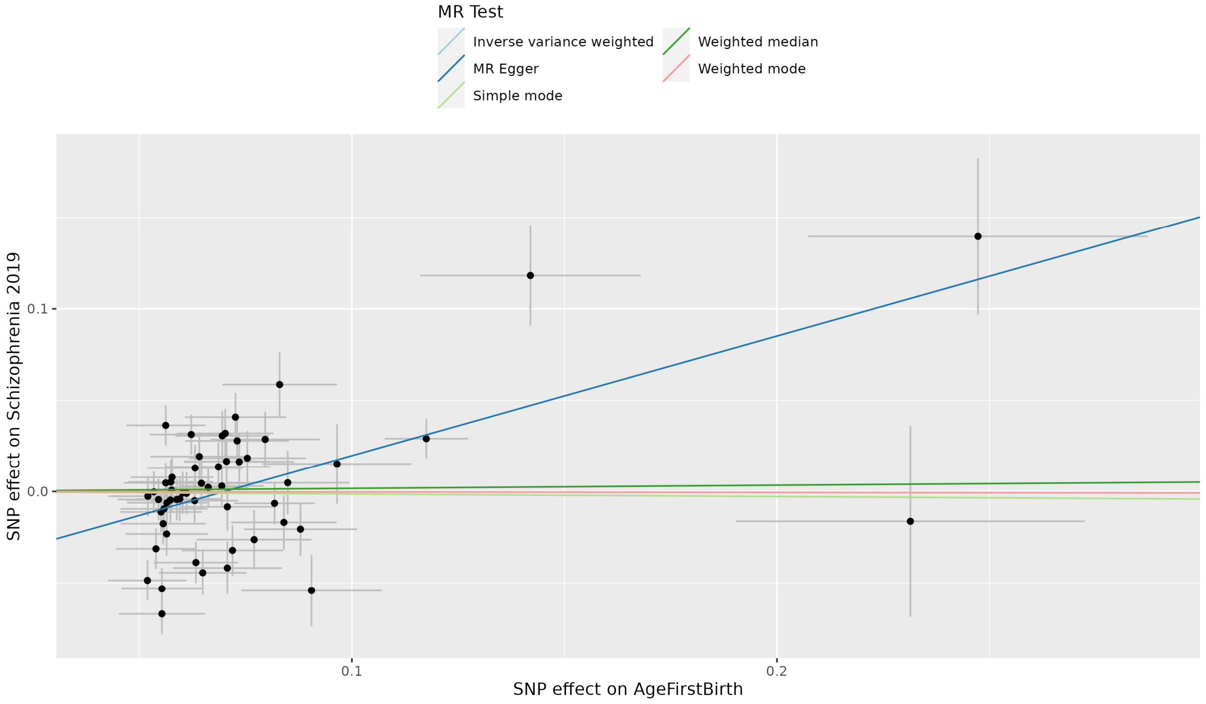


Scatter plot of AgeFirstBirth on Schizophrenia 2019

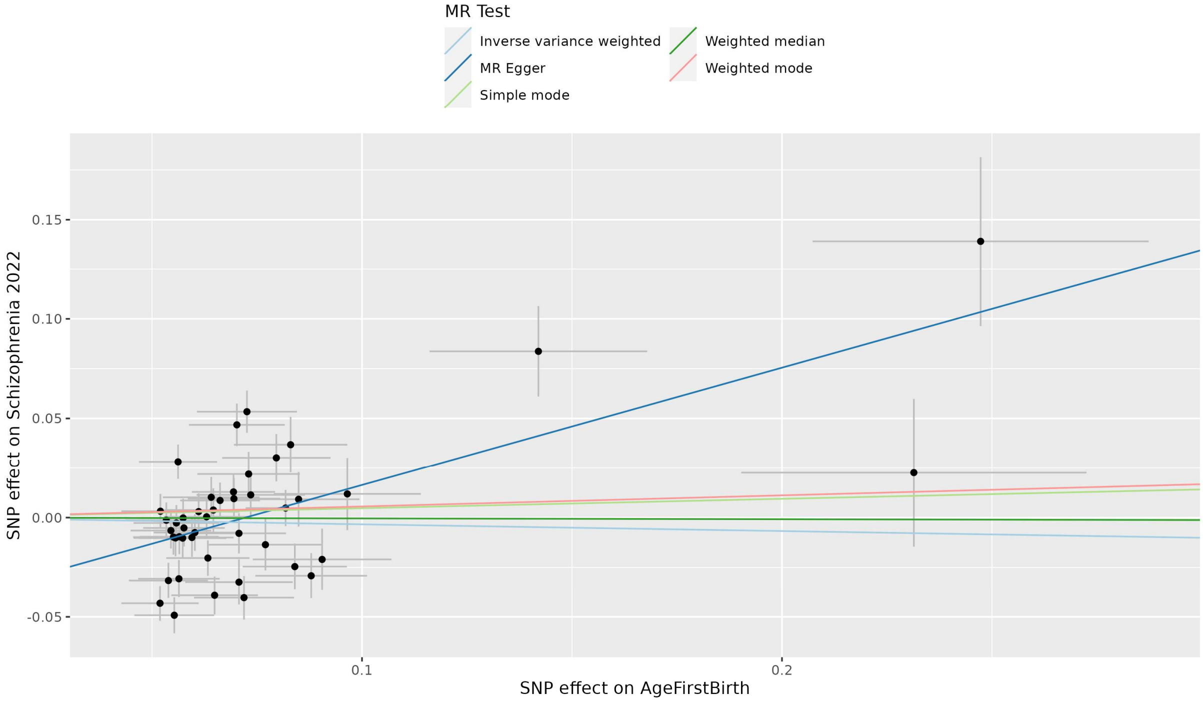


Scatter plot of AgeFirstBirth on Schizophrenia 2022

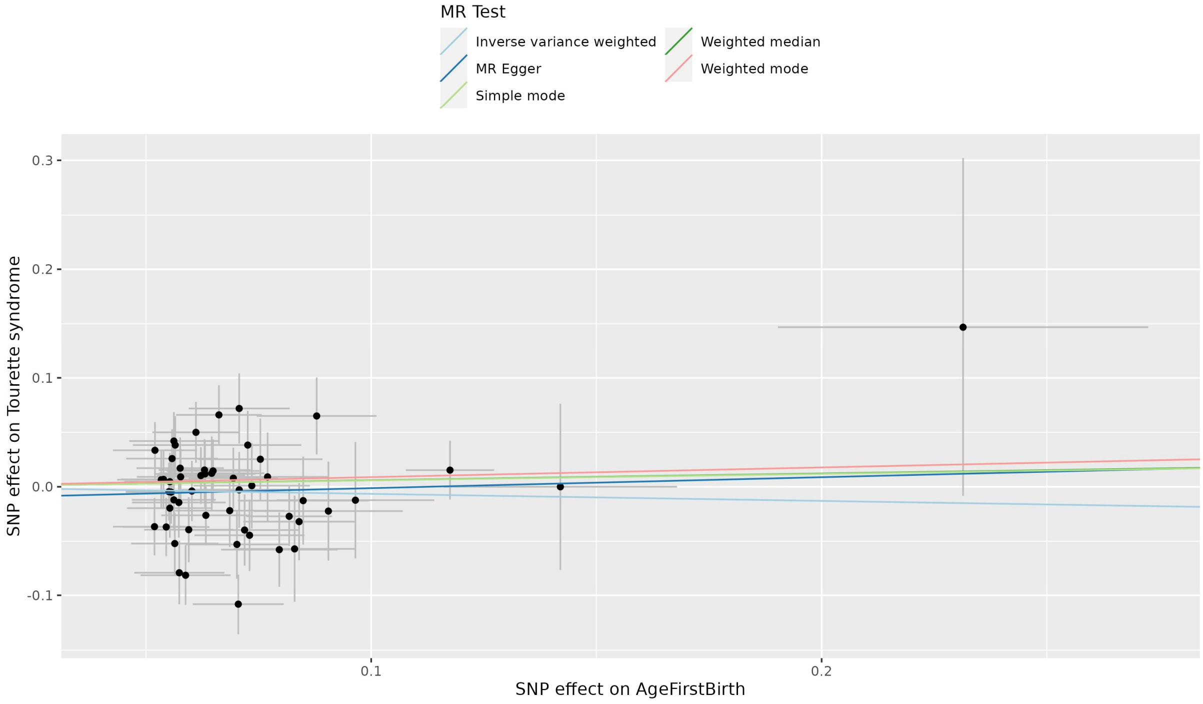


Scatter plot of AgeFirstBirth on Tourette syndrome

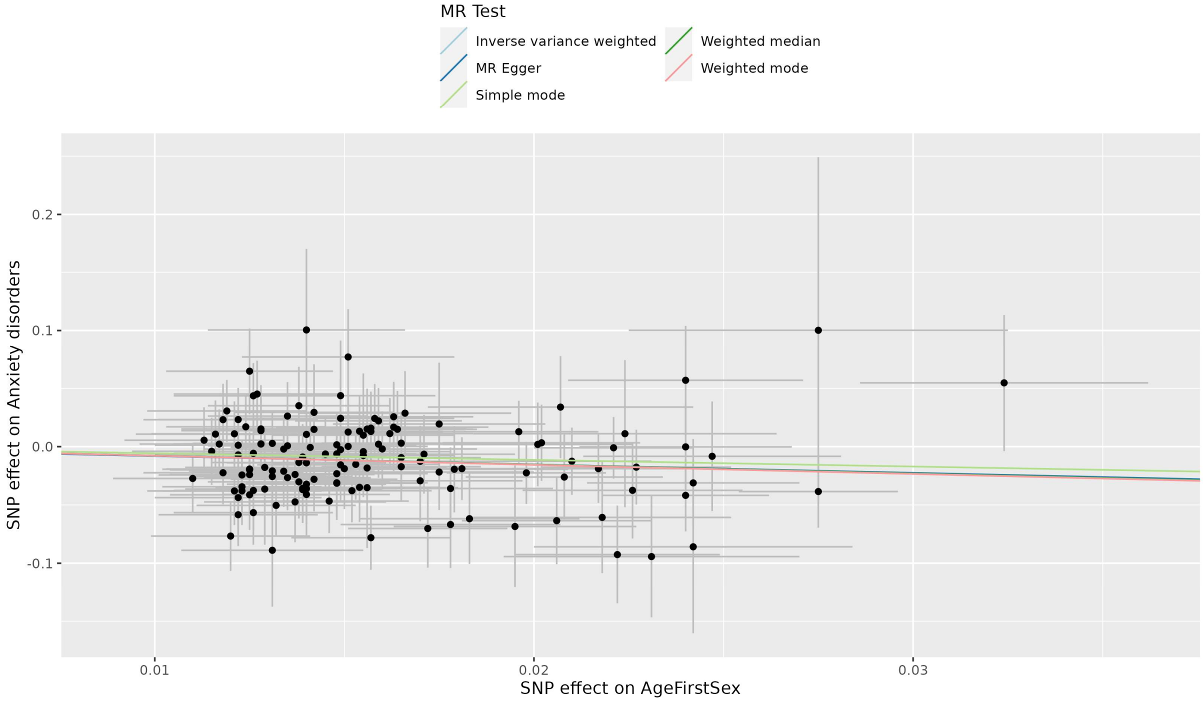


Scatter plot of AgeFirstSex on Anxiety disorders

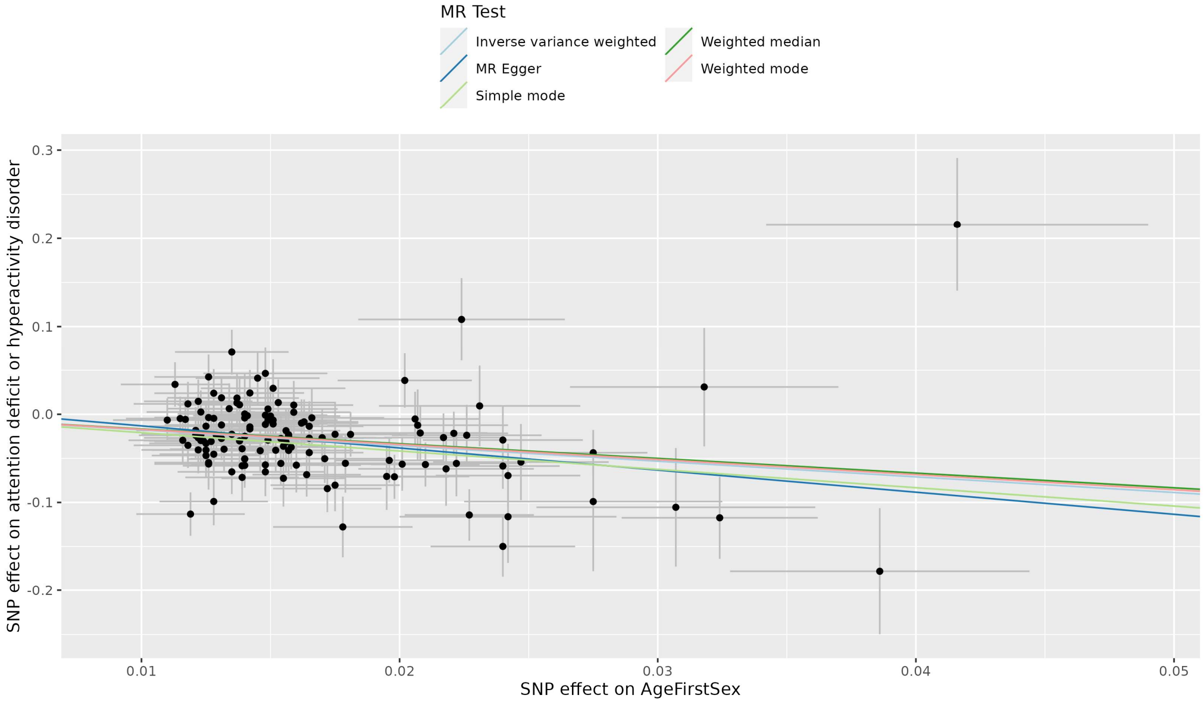


Scatter plot of AgeFirstSex on attention deficit or hyperactivity disorder

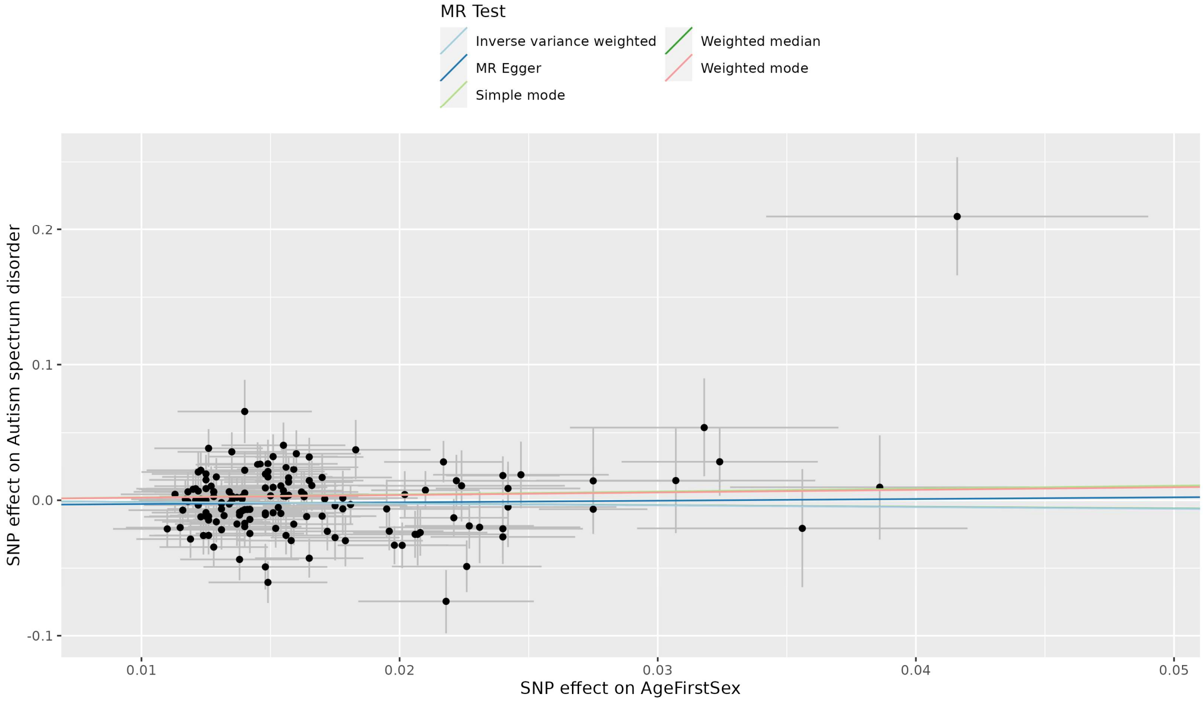


Scatter plot of AgeFirstSex on Autism spectrum disorder

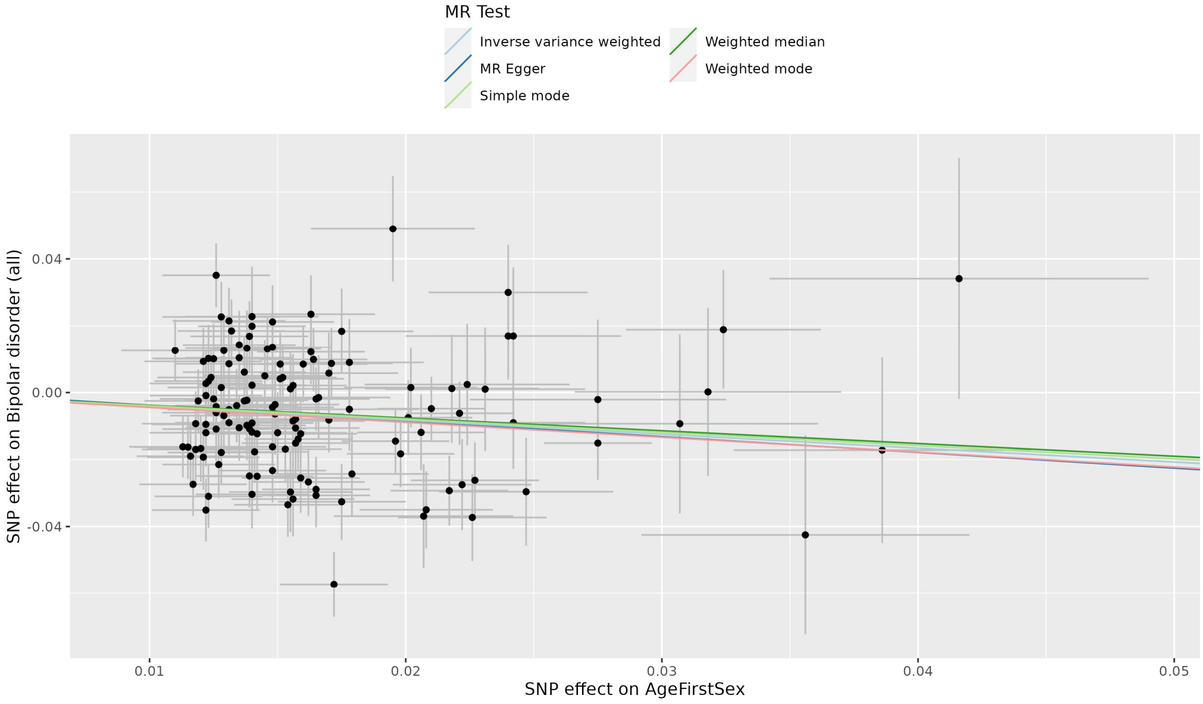


Scatter plot of AgeFirstSex on Bipolar disorder (all)

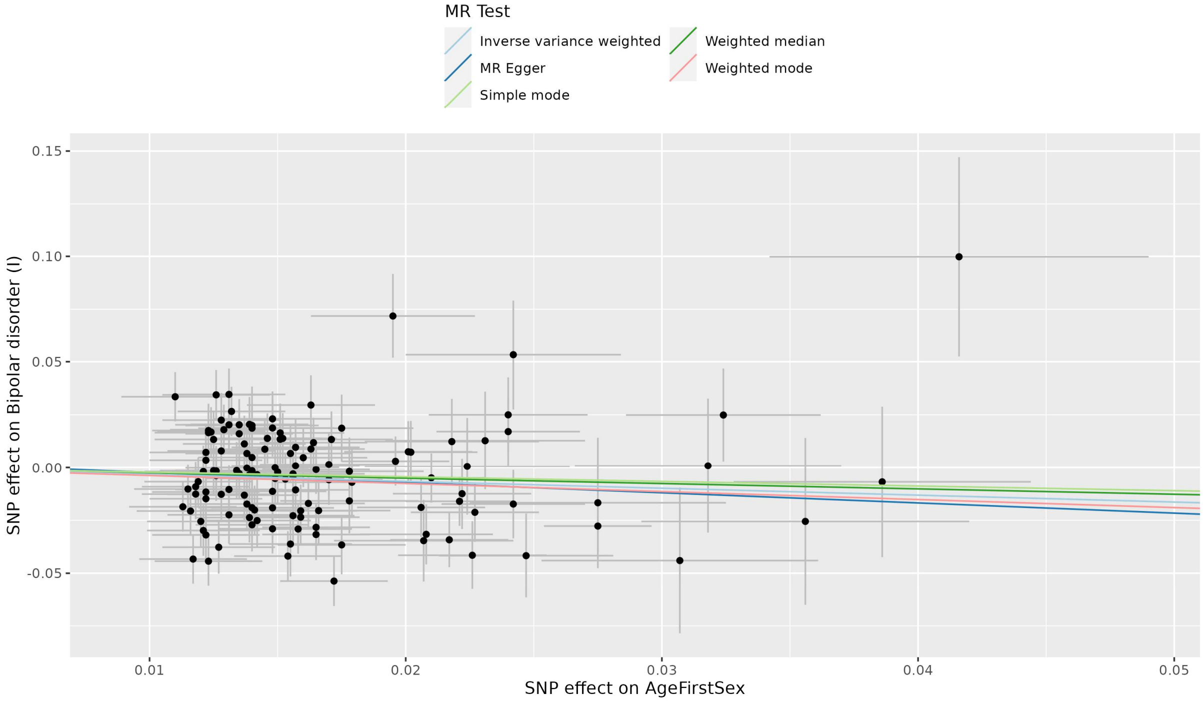


Scatter plot of AgeFirstSex on Bipolar disorder (I)

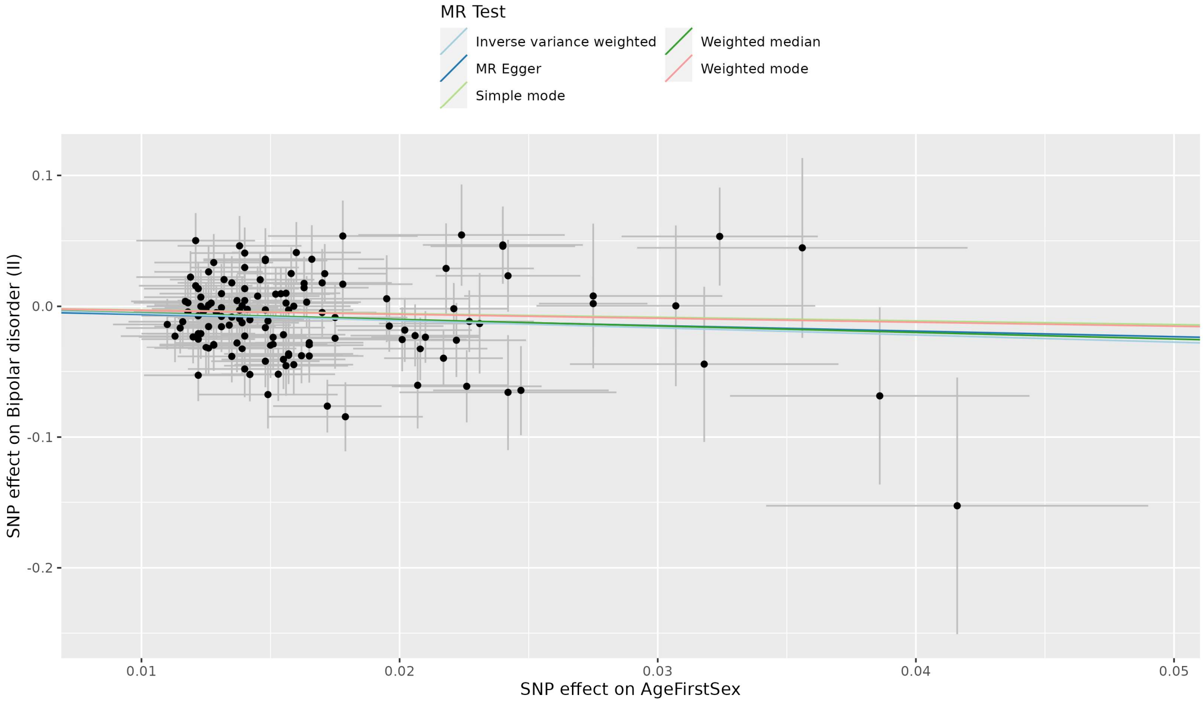


Scatter plot of AgeFirstSex on Bipolar disorder (II)

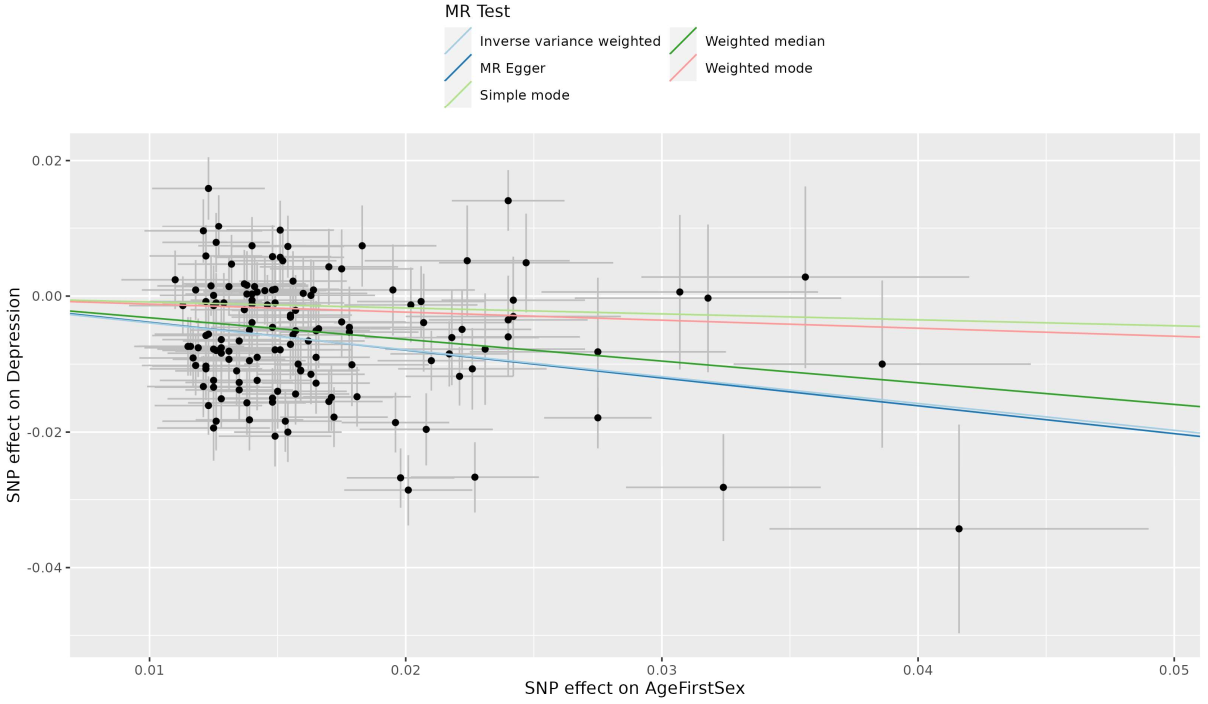


Scatter plot of AgeFirstSex on Depression

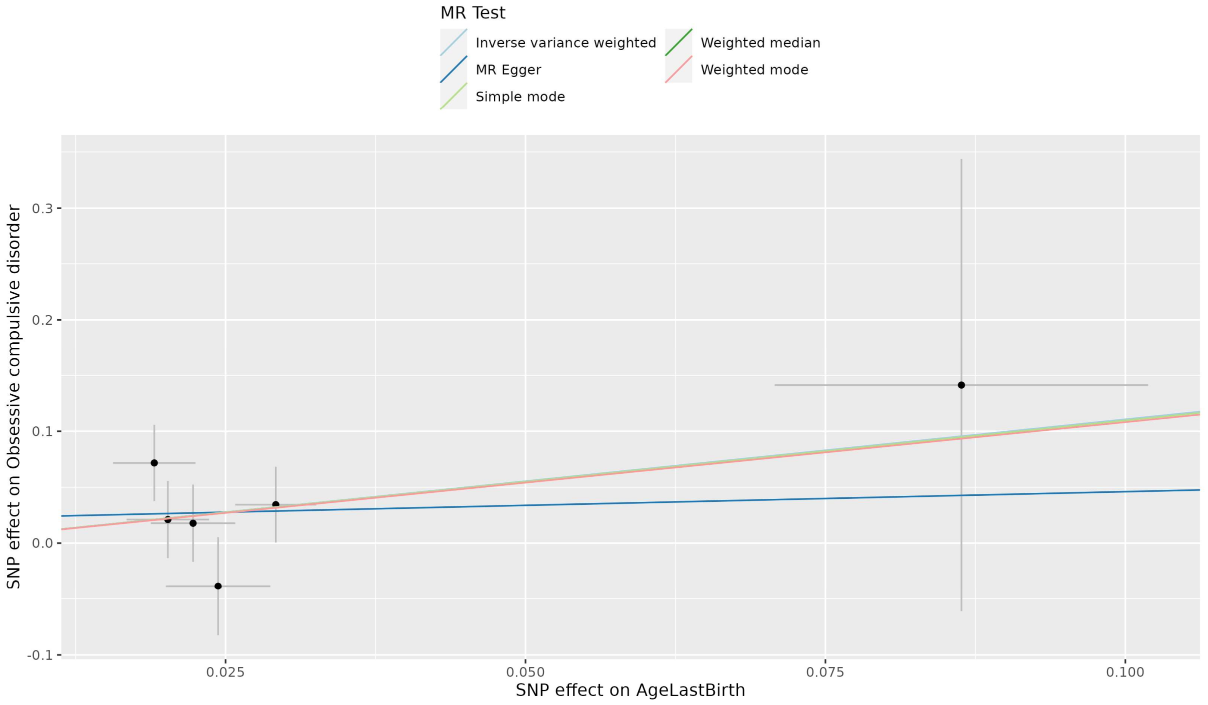


Scatter plot of AgeFirstSex on Obsessive compulsive disorder

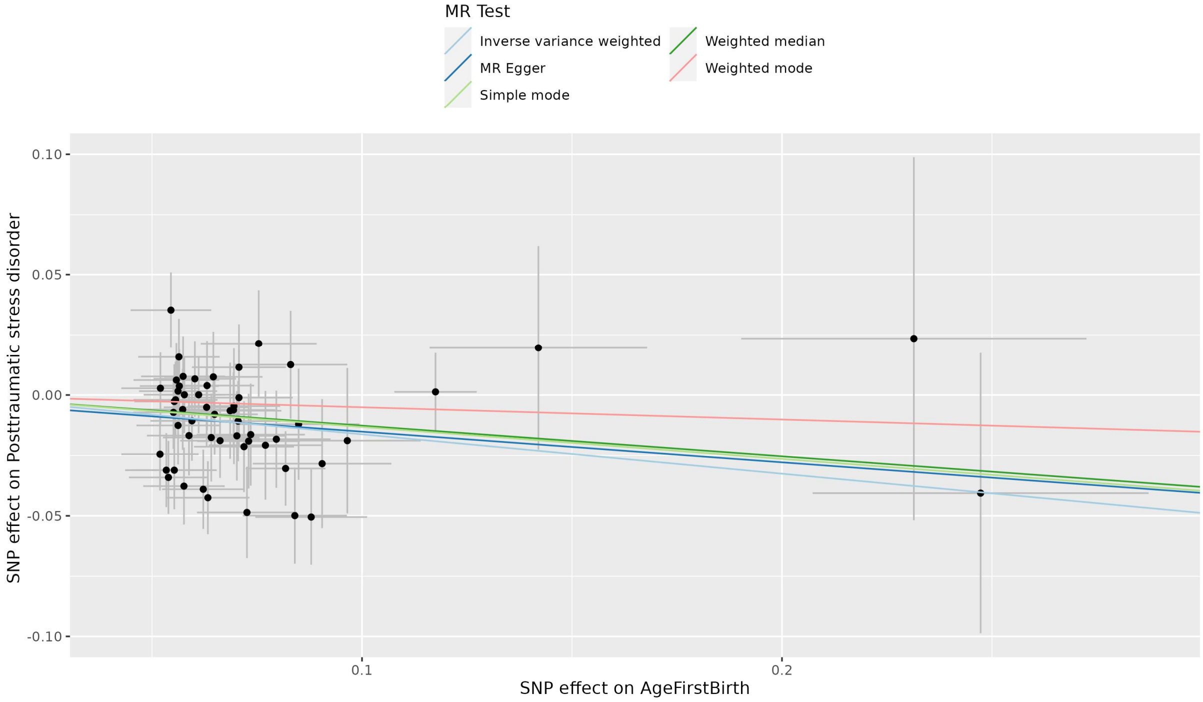


Scatter plot of AgeFirstSex on Posttraumatic stress disorder

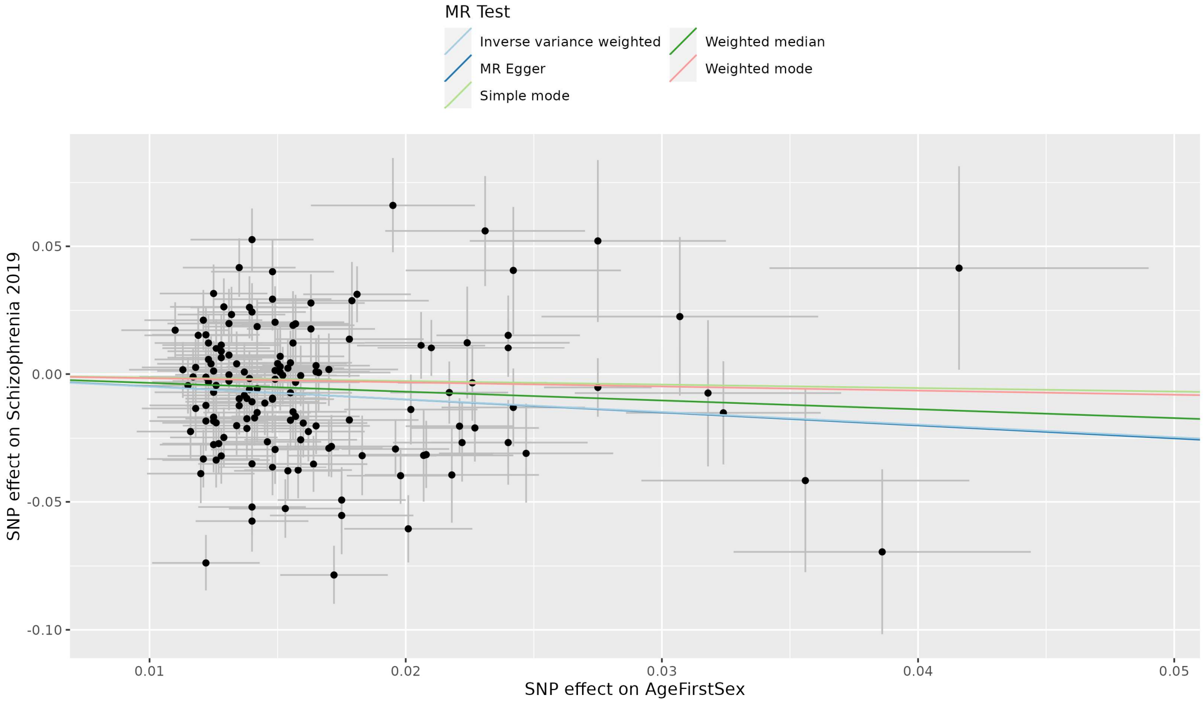


Scatter plot of AgeFirstSex on Schizophrenia 2019

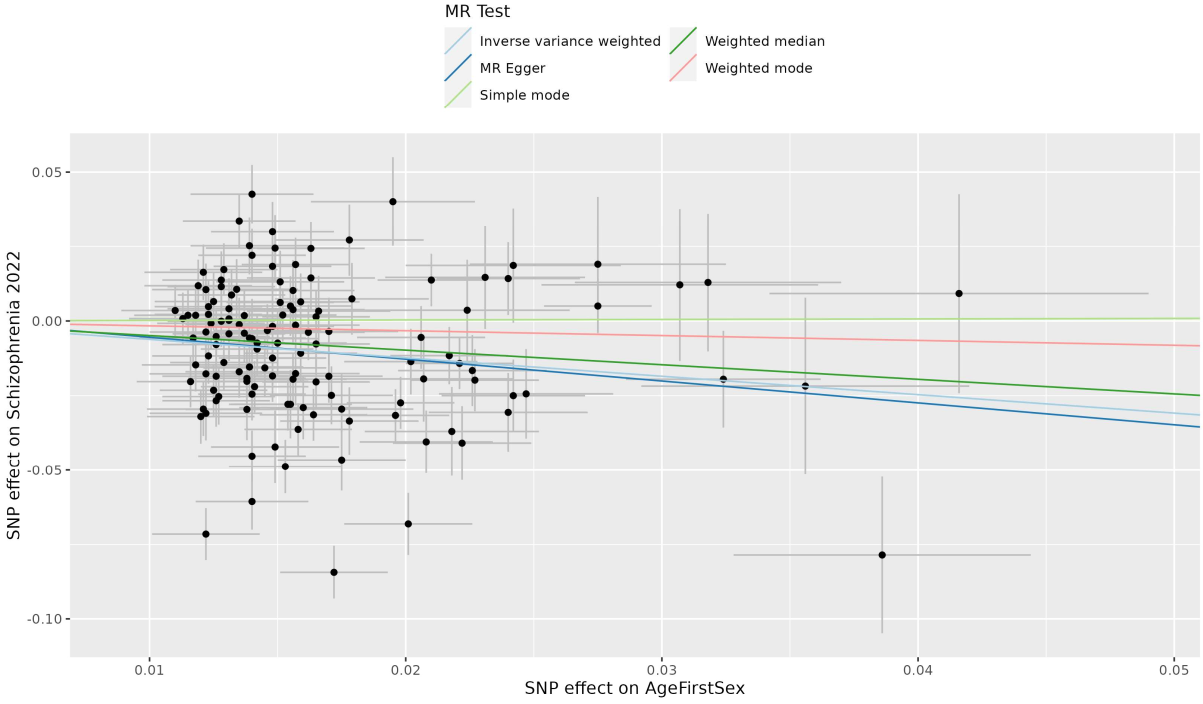


Scatter plot of AgeFirstSex on Schizophrenia 2022

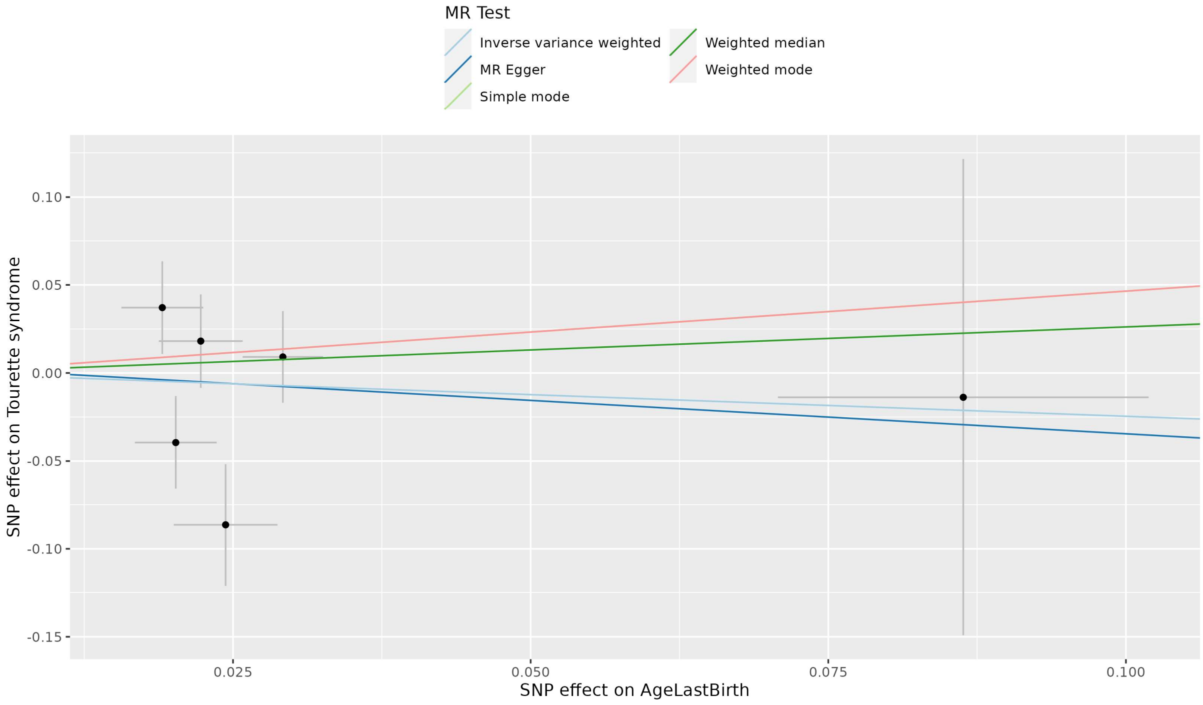


Scatter plot of AgeFirstSex on Tourette syndrome

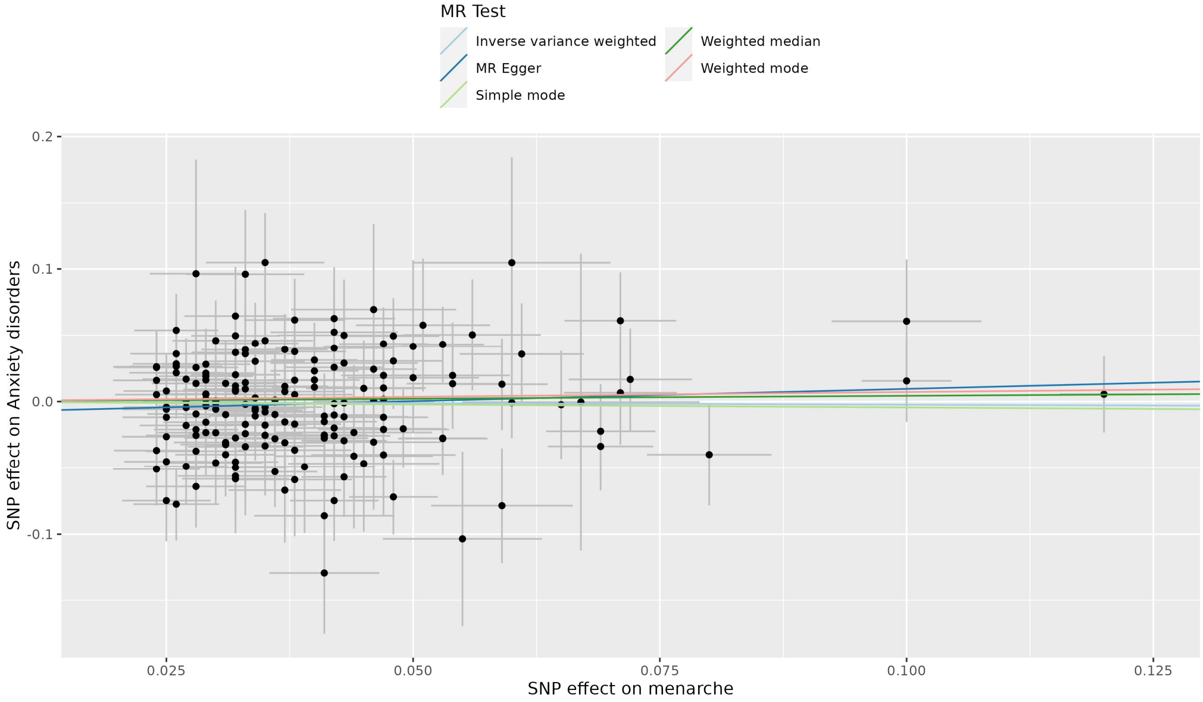


Scatter plot of AgeLastBirth on Anxiety disorders

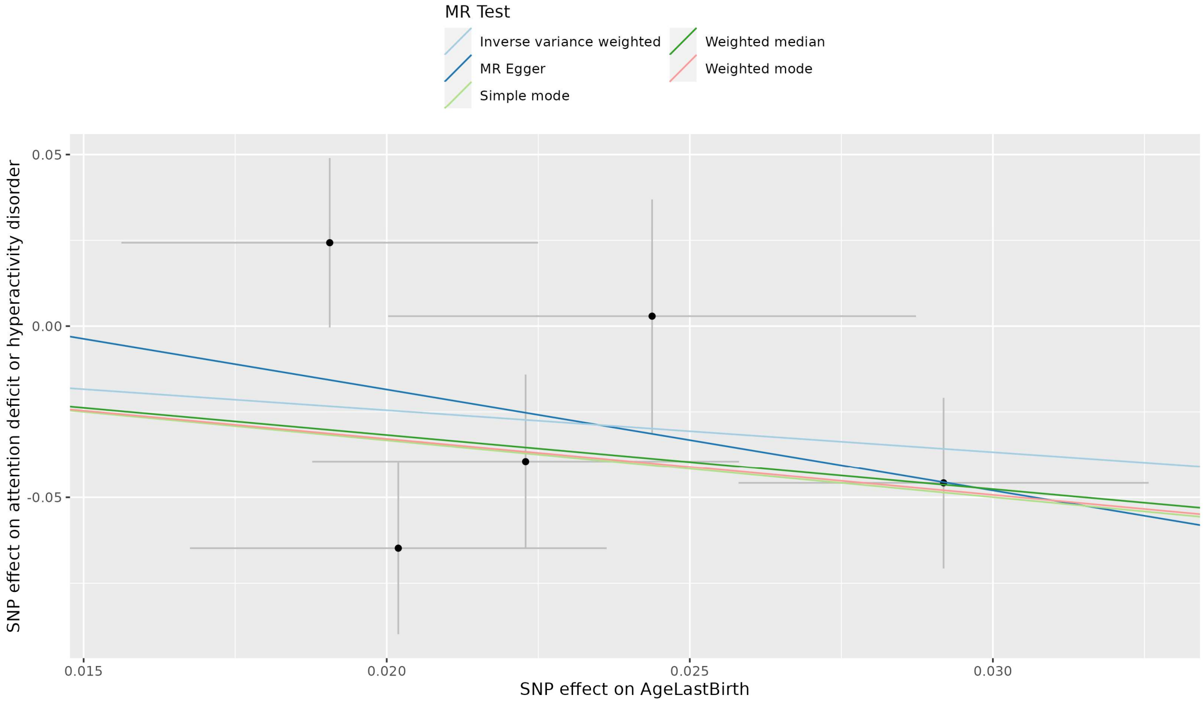


Scatter plot of AgeLastBirth on attention deficit or hyperactivity disorder

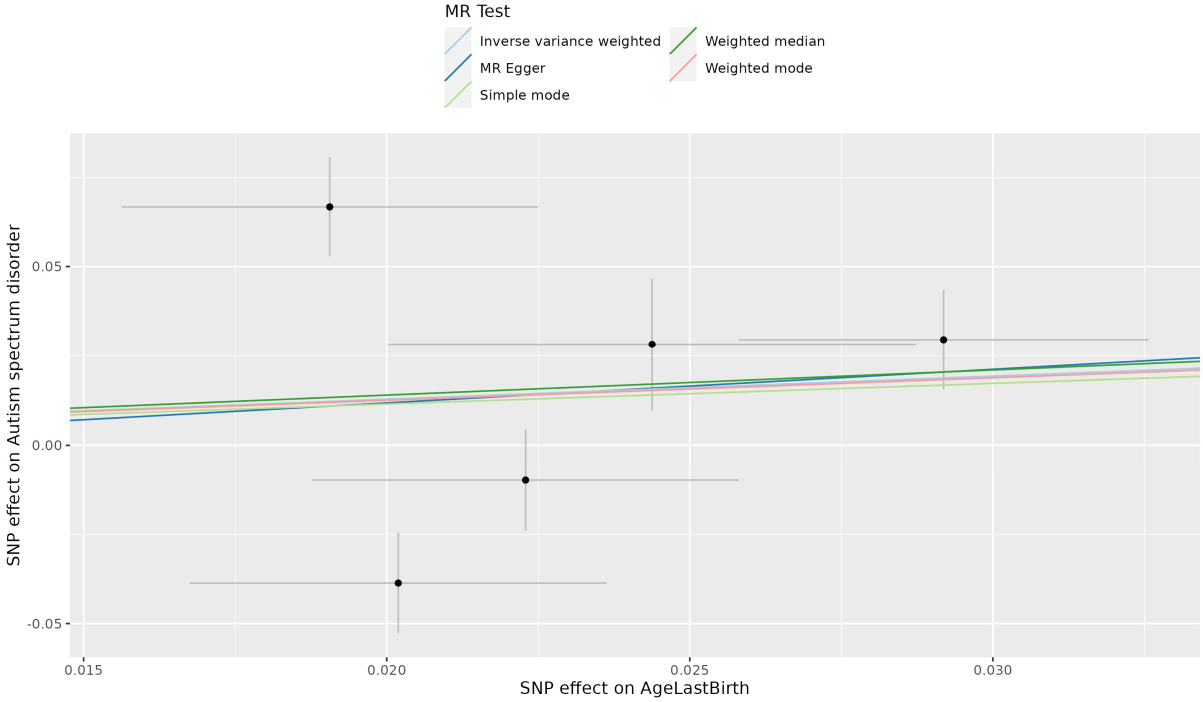


Scatter plot of AgeLastBirth on Autism spectrum disorder

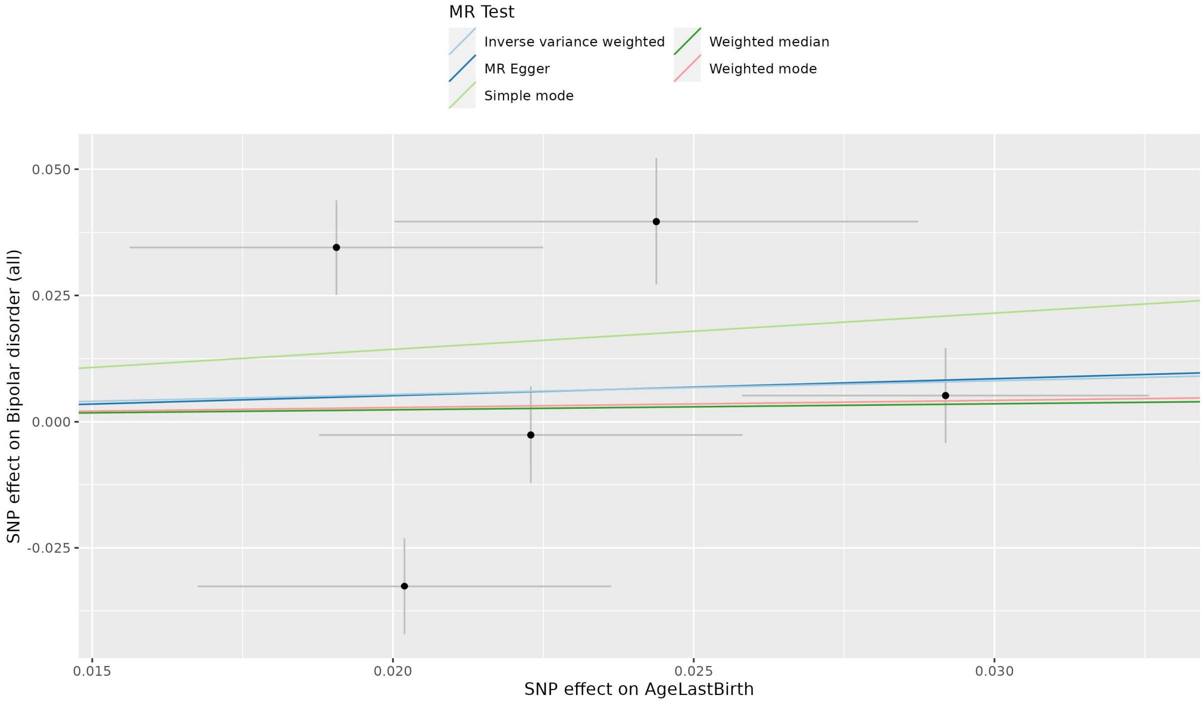


Scatter plot of AgeLastBirth on Bipolar disorder (all)

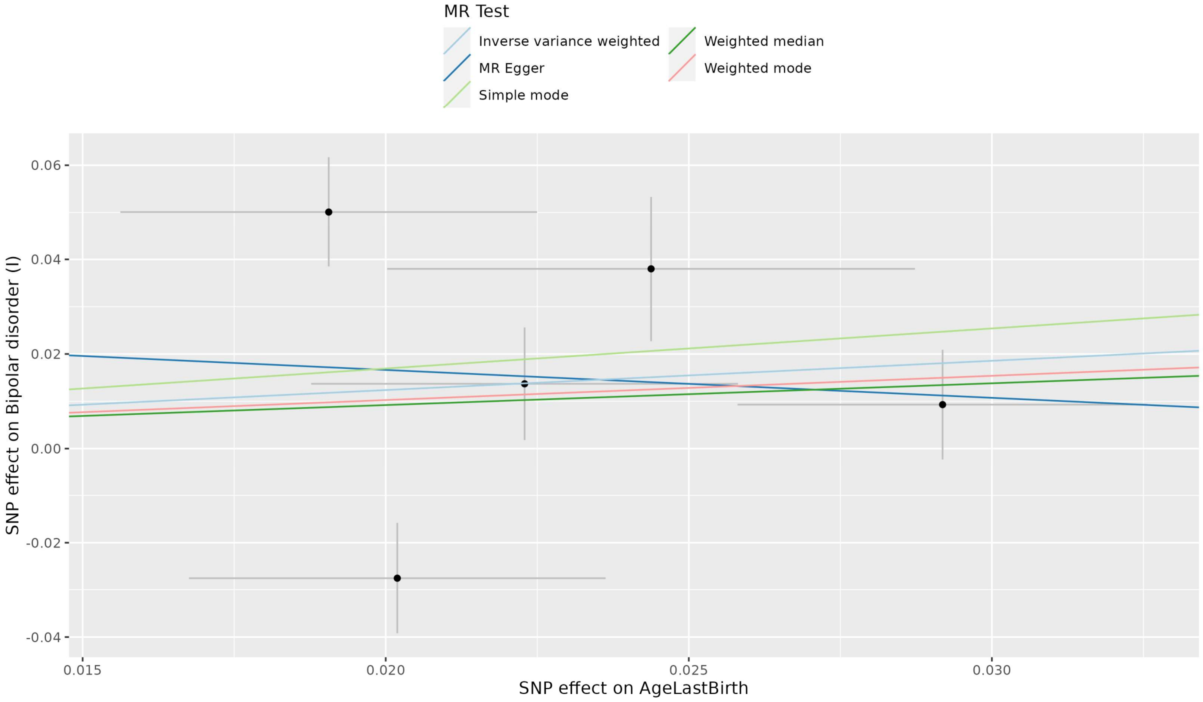


Scatter plot of AgeLastBirth on Bipolar disorder (I)

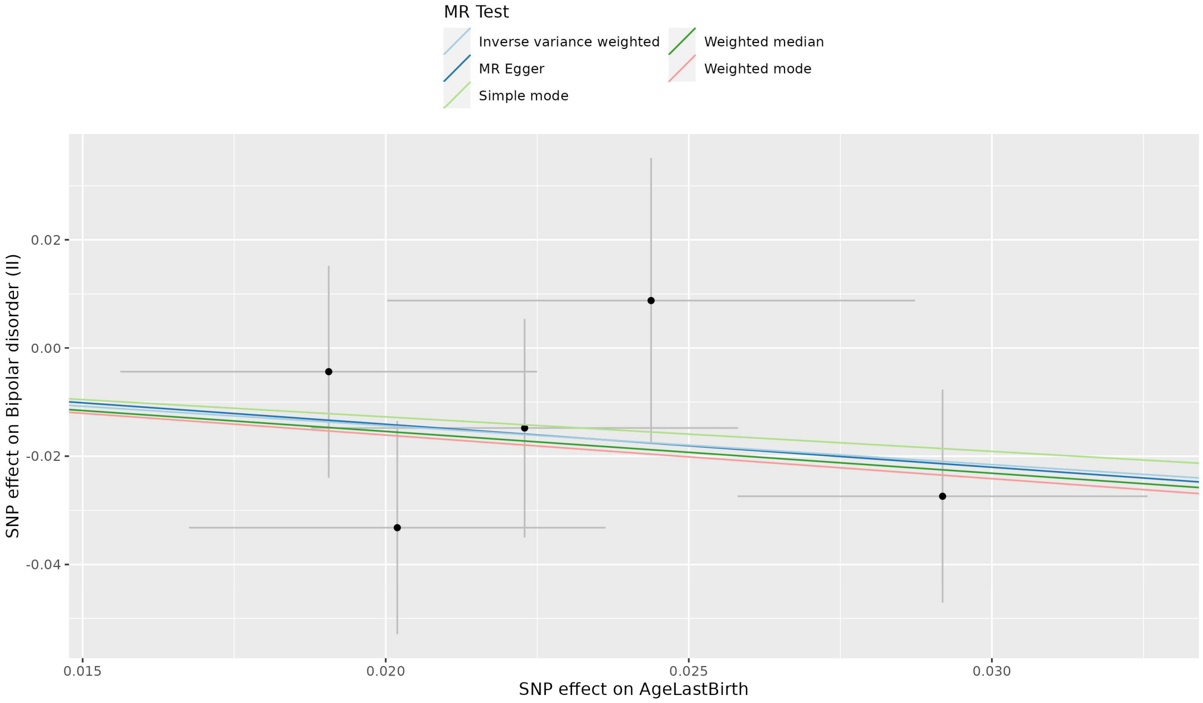


Scatter plot of AgeLastBirth on Bipolar disorder (II)

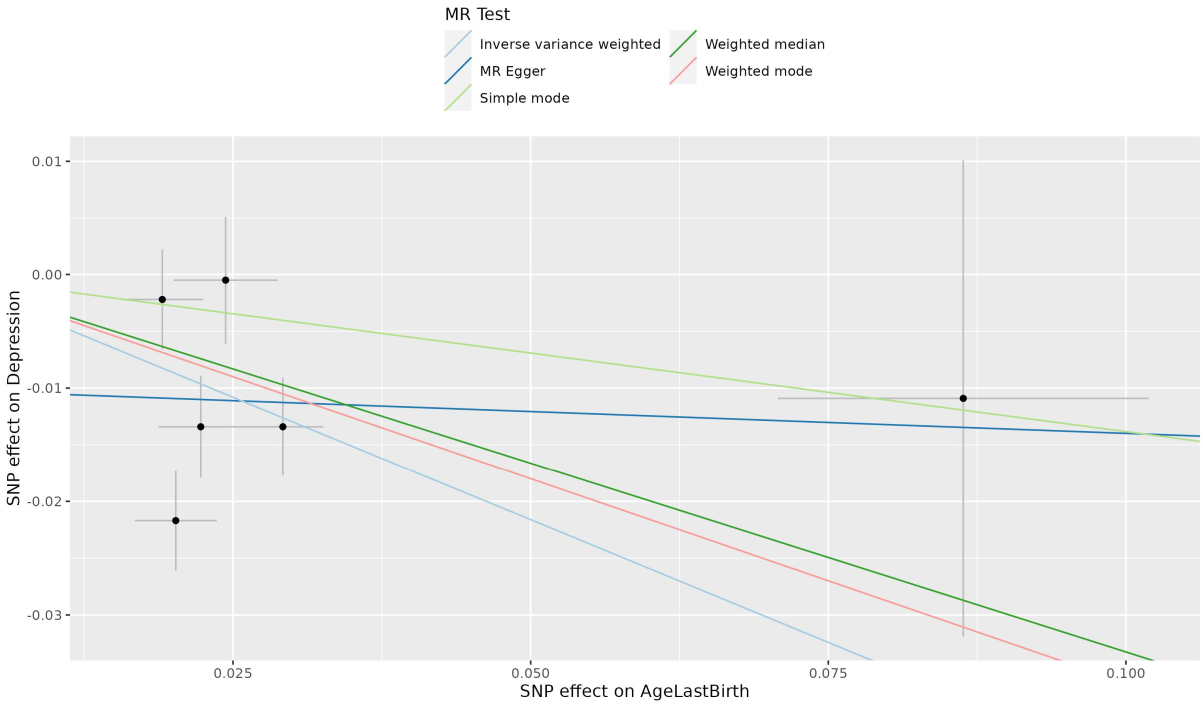


Scatter plot of AgeLastBirth on Depression

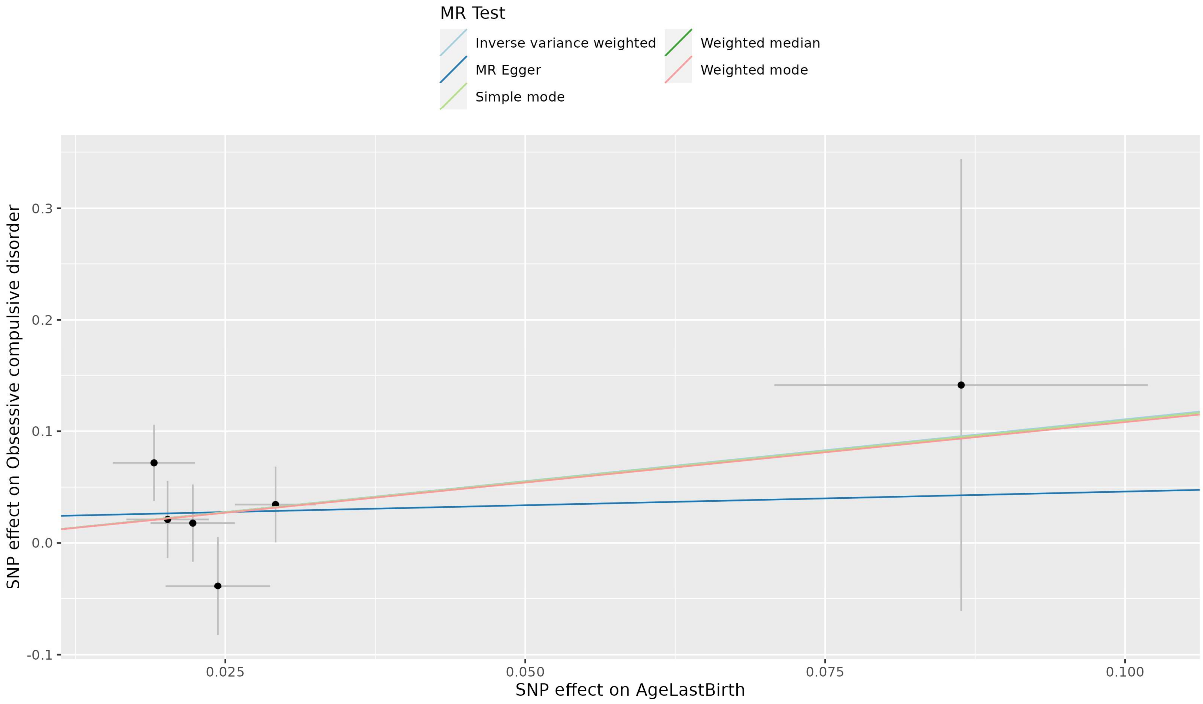


Scatter plot of AgeLastBirth on Obsessive compulsive disorder

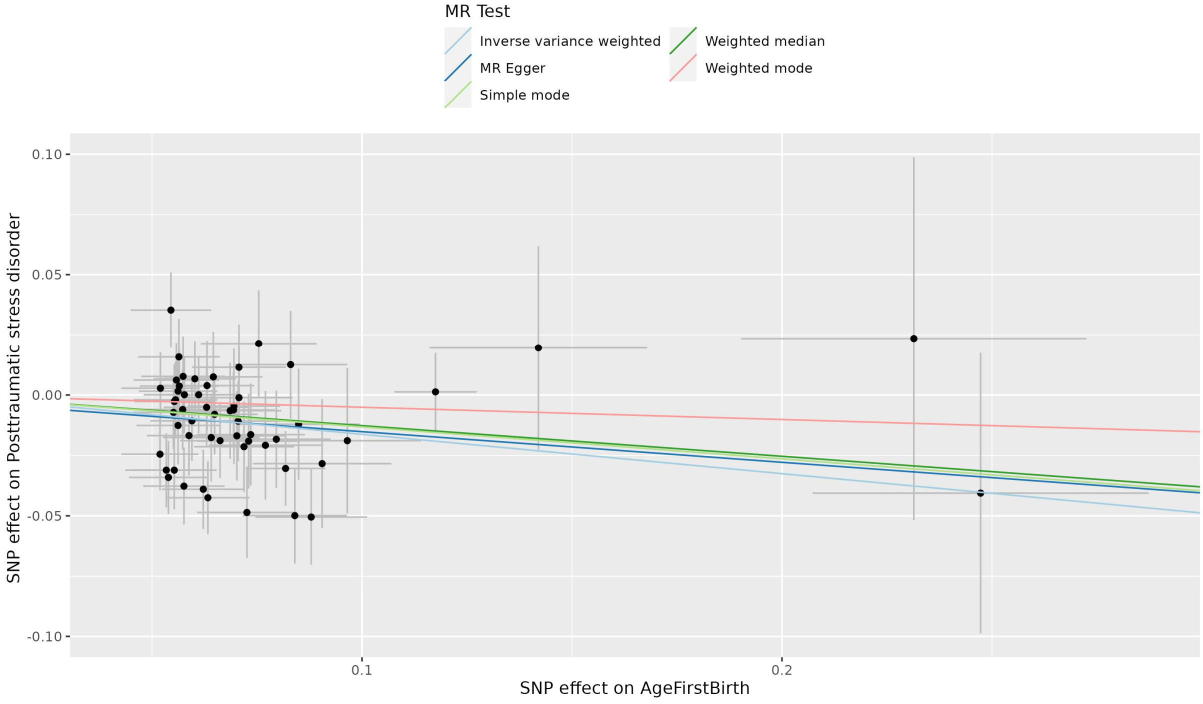


Scatter plot of AgeLastBirth on Posttraumatic stress disorder

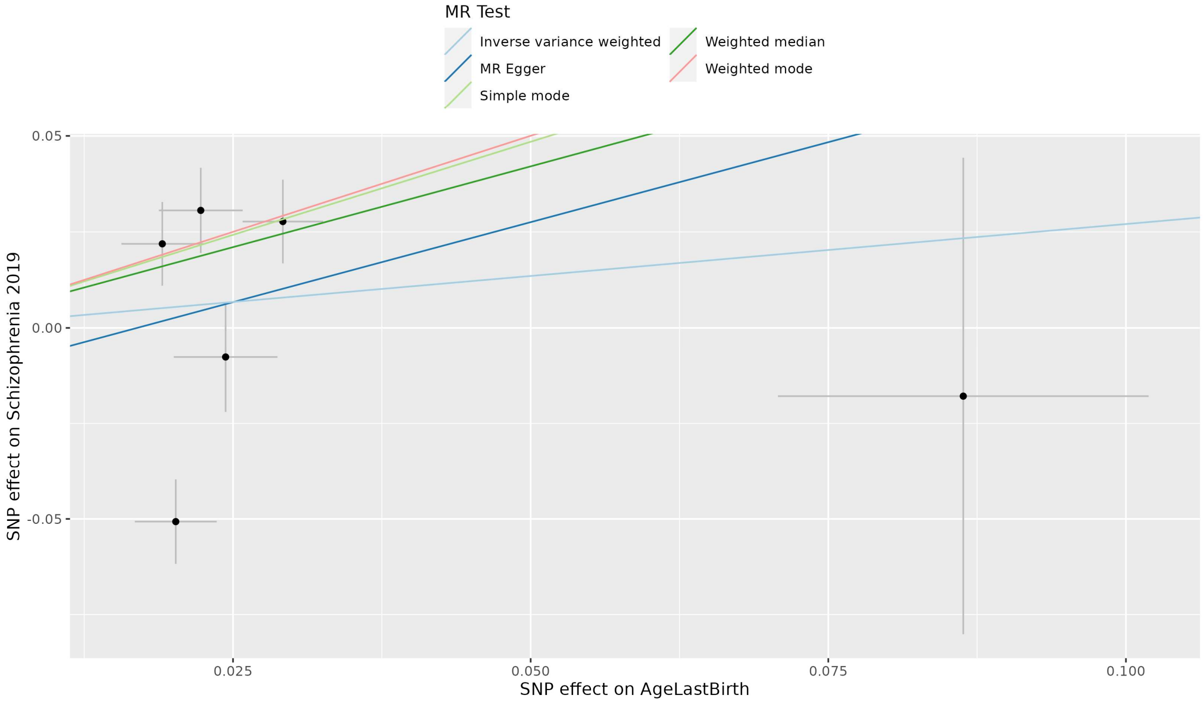


Scatter plot of AgeLastBirth on Schizophrenia 2019

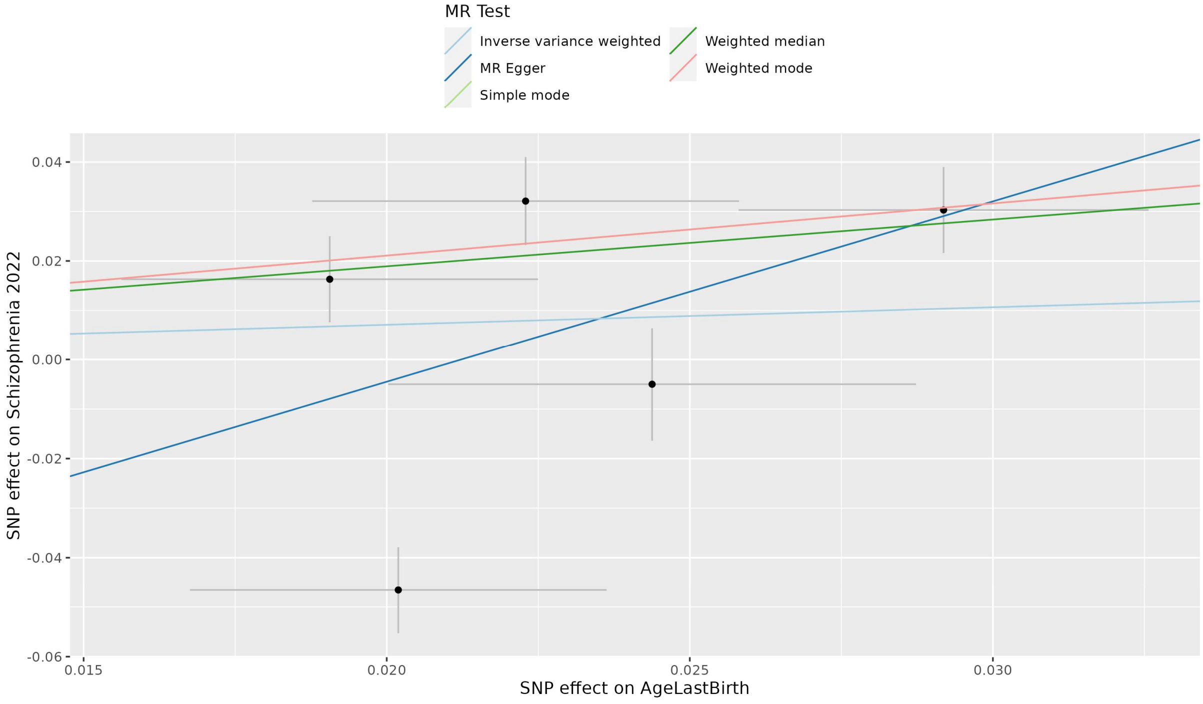


Scatter plot of AgeLastBirth on Schizophrenia 2022

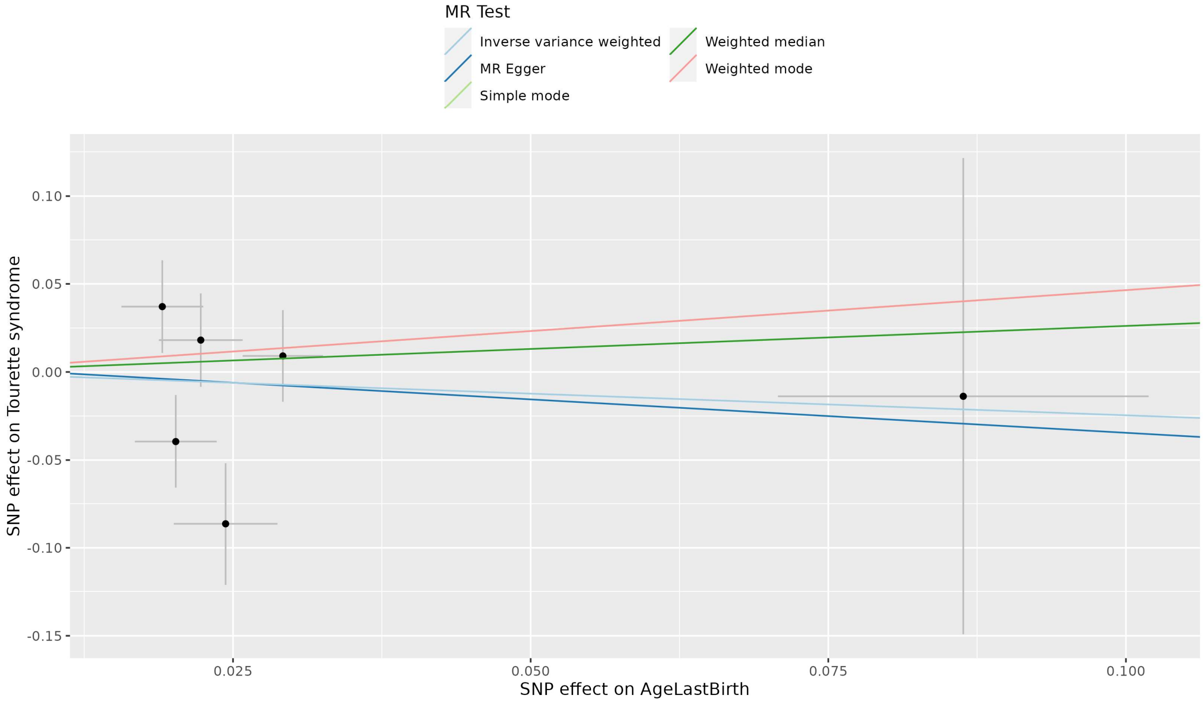


Scatter plot of AgeLastBirth on Tourette syndrome

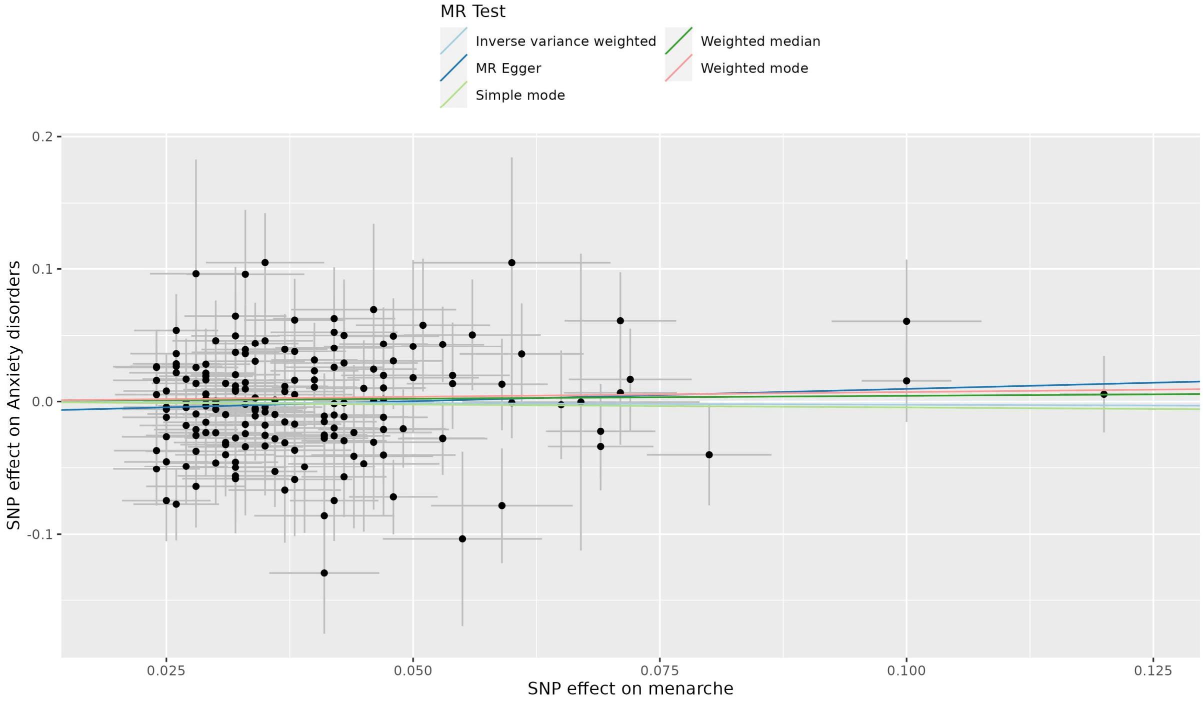


Scatter plot of menarche on Anxiety disorders

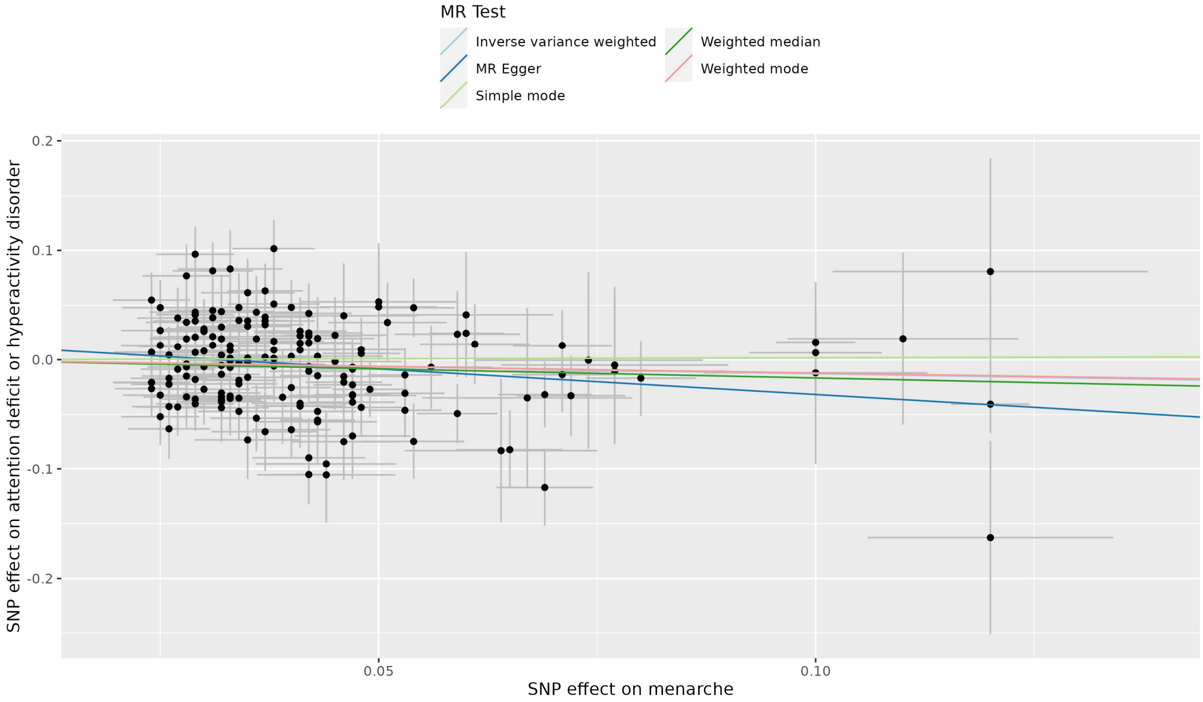


Scatter plot of menarche on attention deficit or hyperactivity disorder

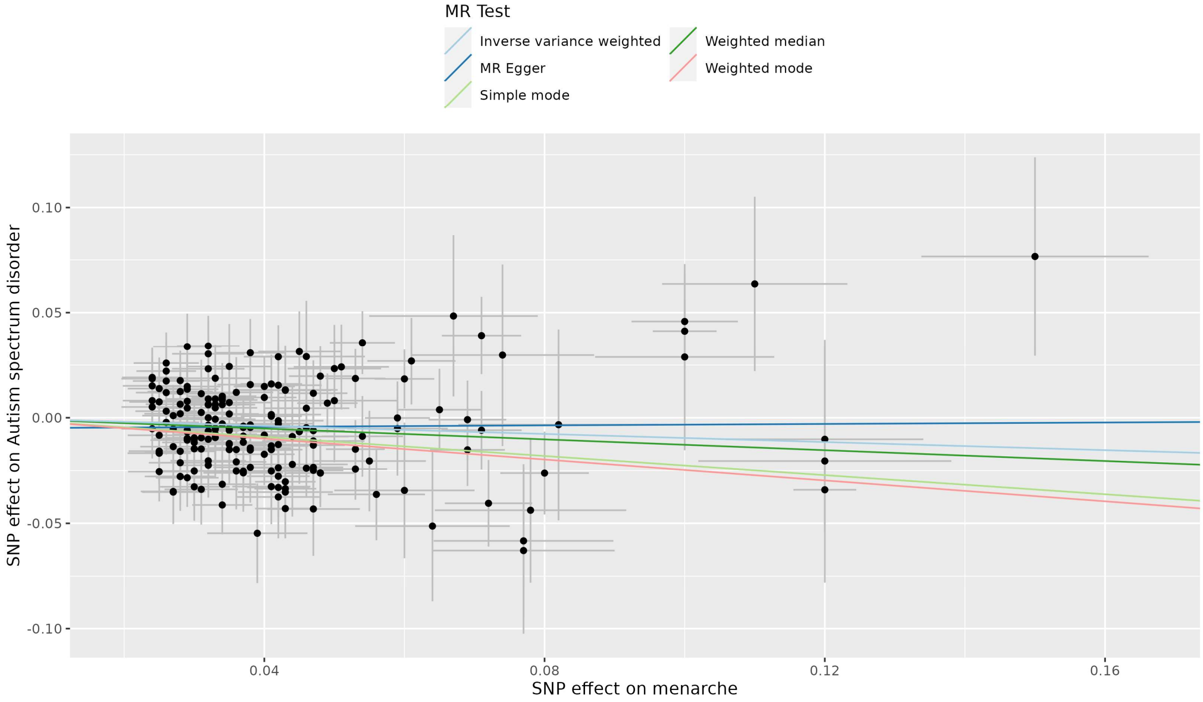


Scatter plot of menarche on Autism spectrum disorder

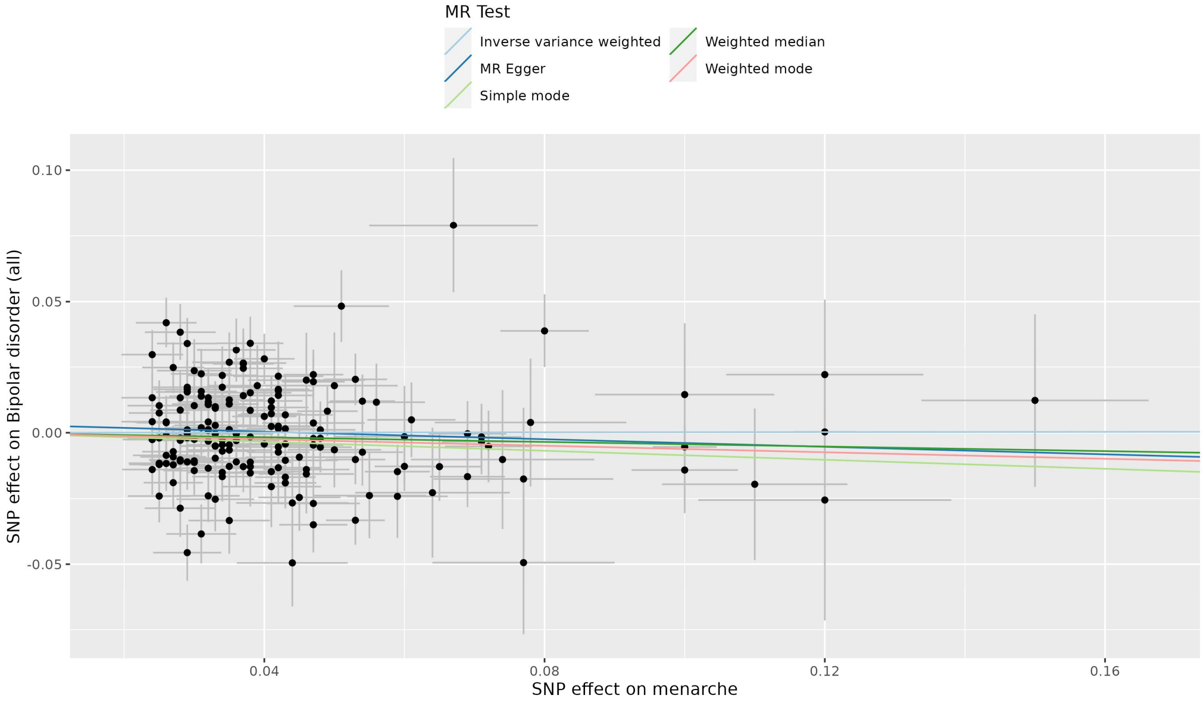


Scatter plot of menarche on Bipolar disorder (all)

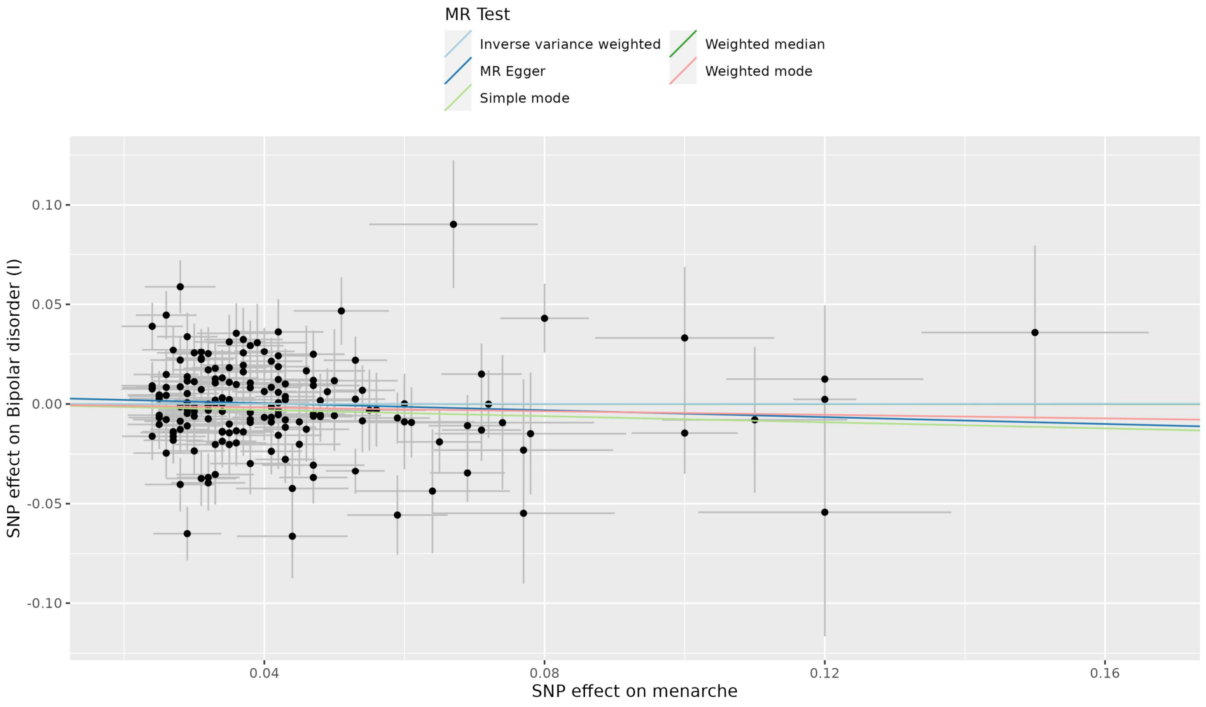


Scatter plot of menarche on Bipolar disorder (I)

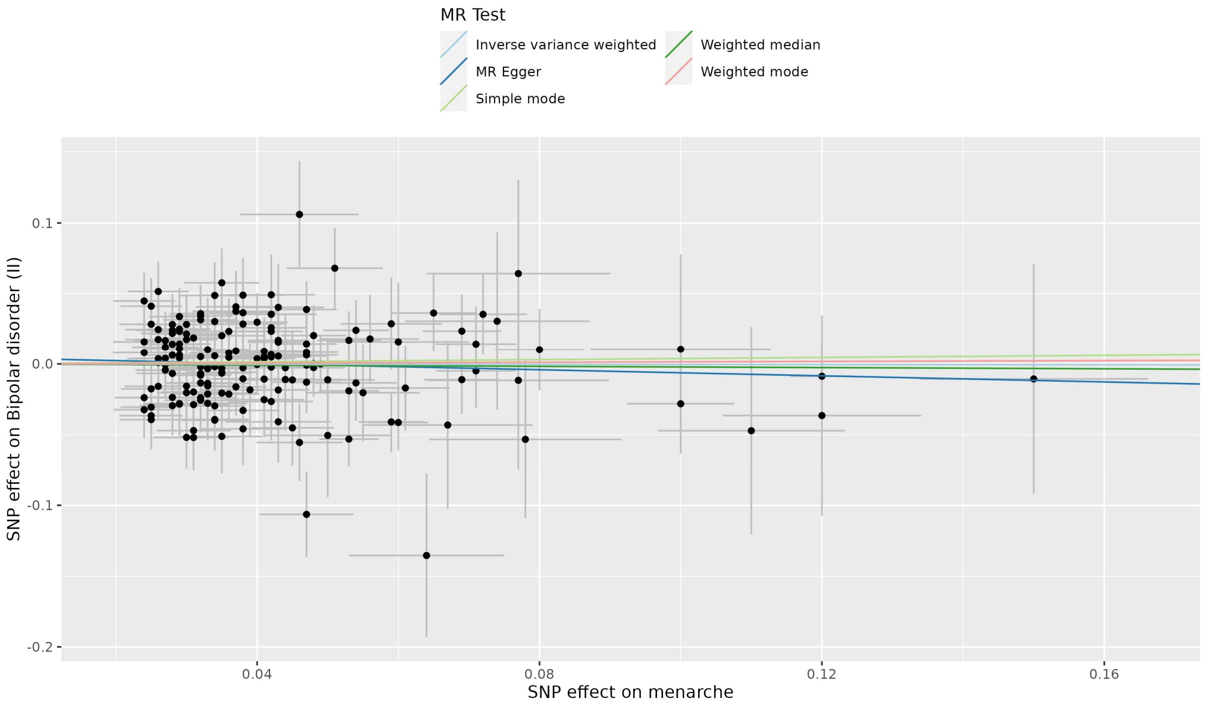


Scatter plot of menarche on Bipolar disorder (II)

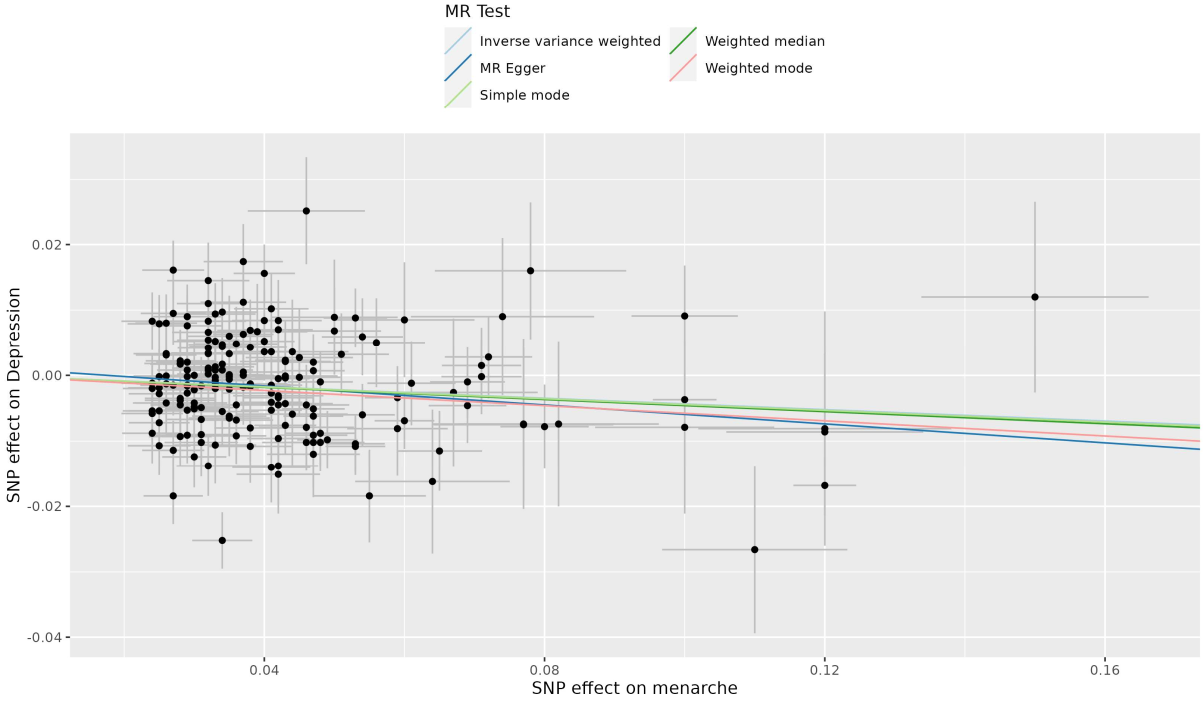


Scatter plot of menarche on Depression

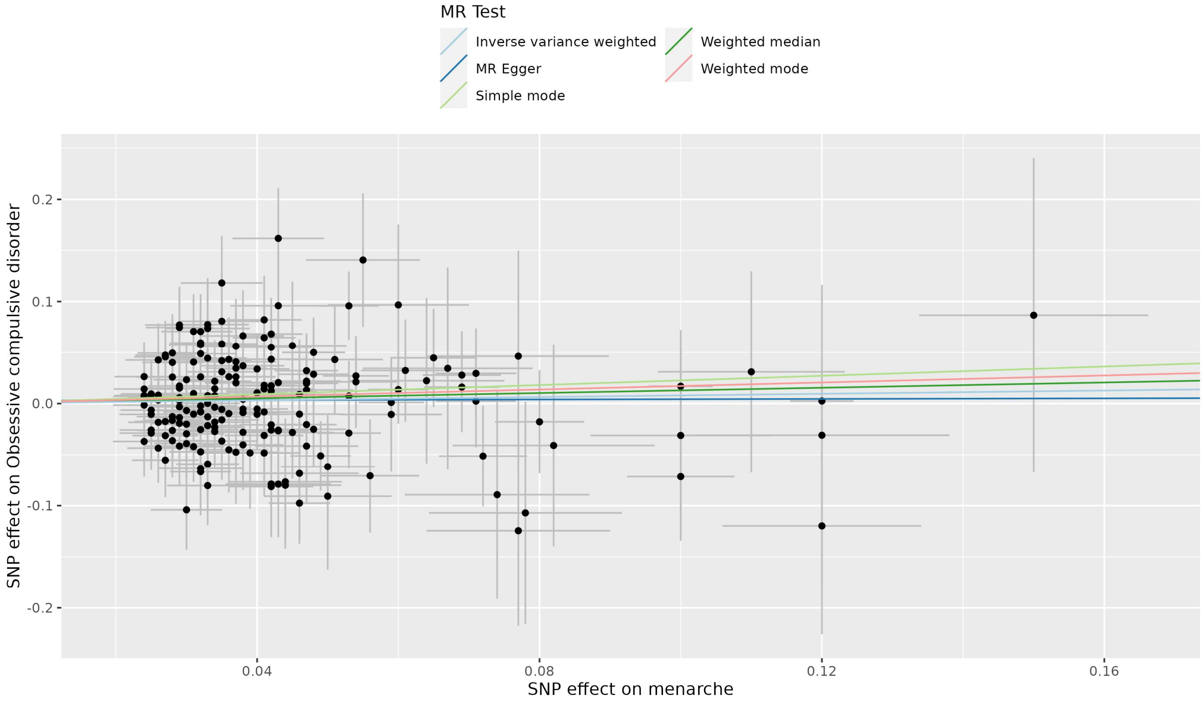


Scatter plot of menarche on Obsessive compulsive disorder

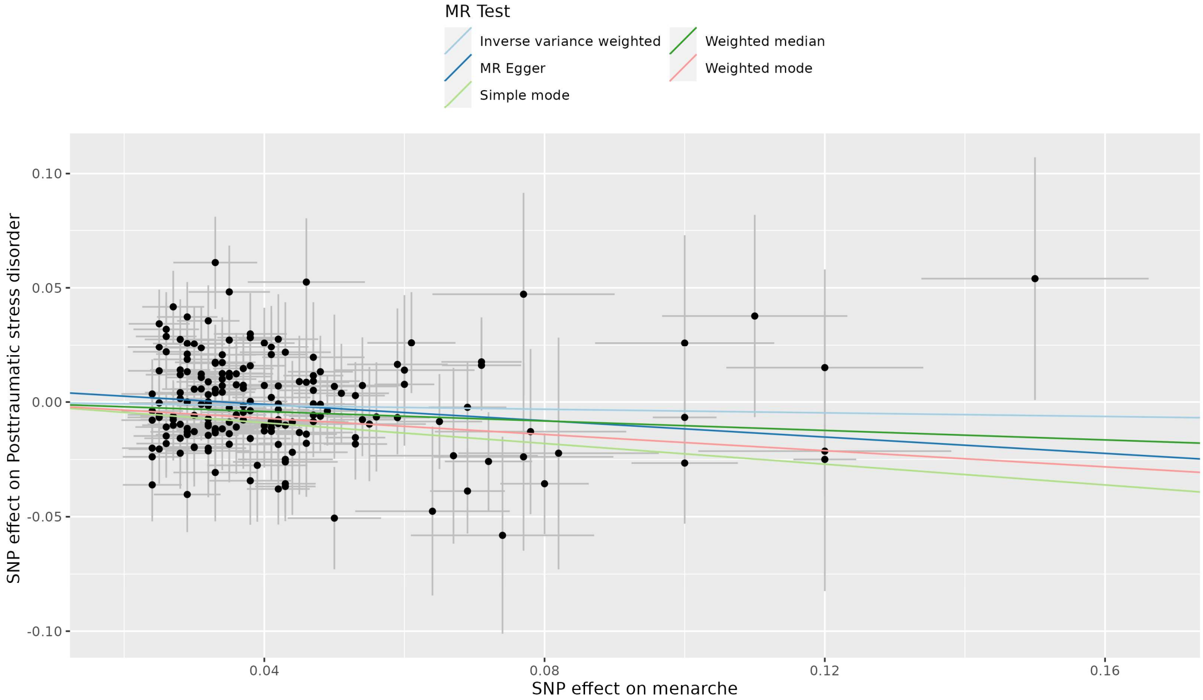


Scatter plot of menarche on Posttraumatic stress disorder

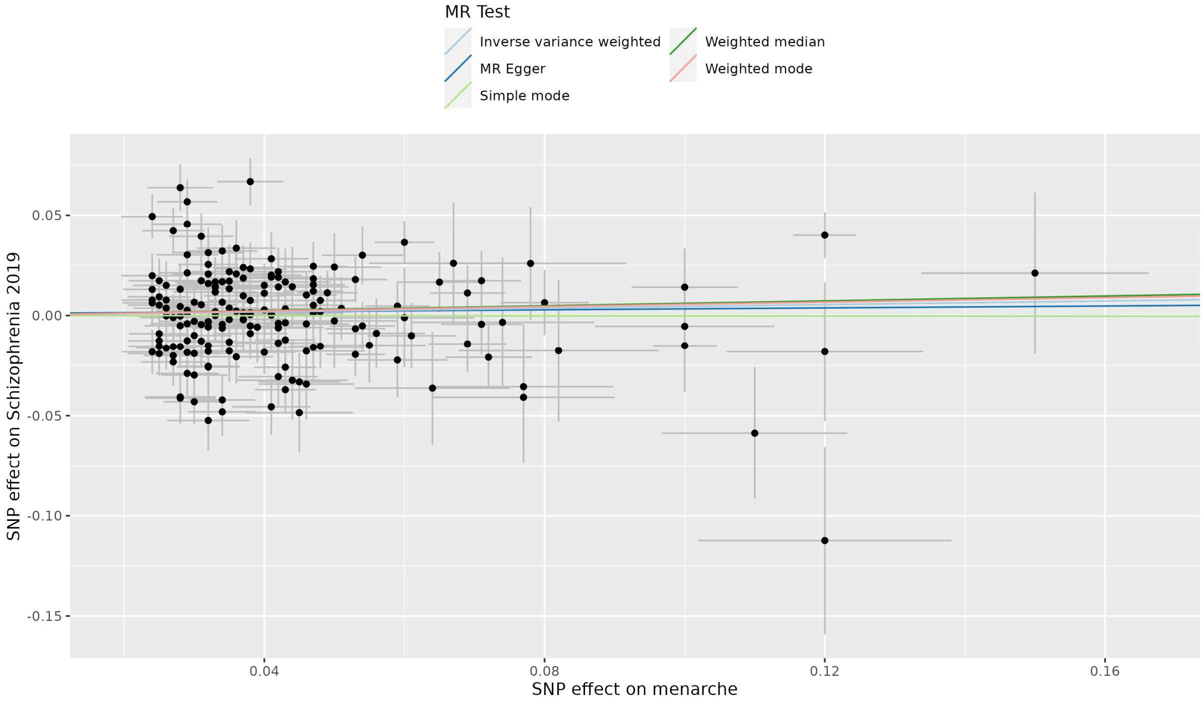


Scatter plot of menarche on Schizophrenia 2019

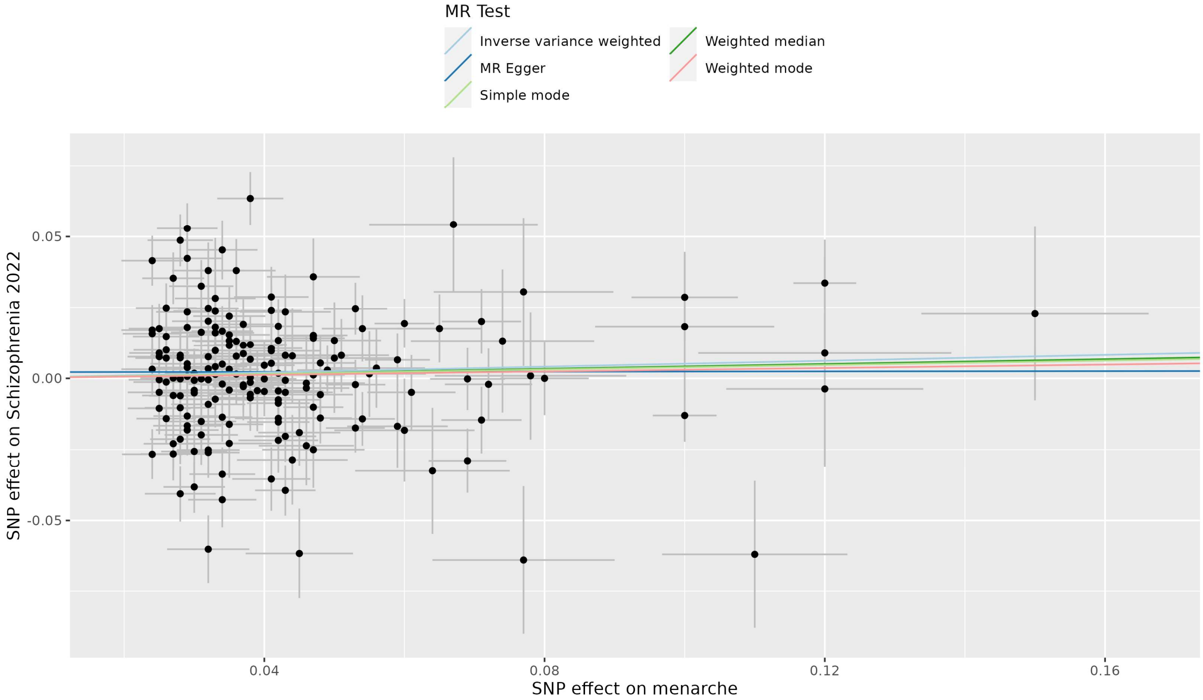


Scatter plot of menarche on Schizophrenia 2022

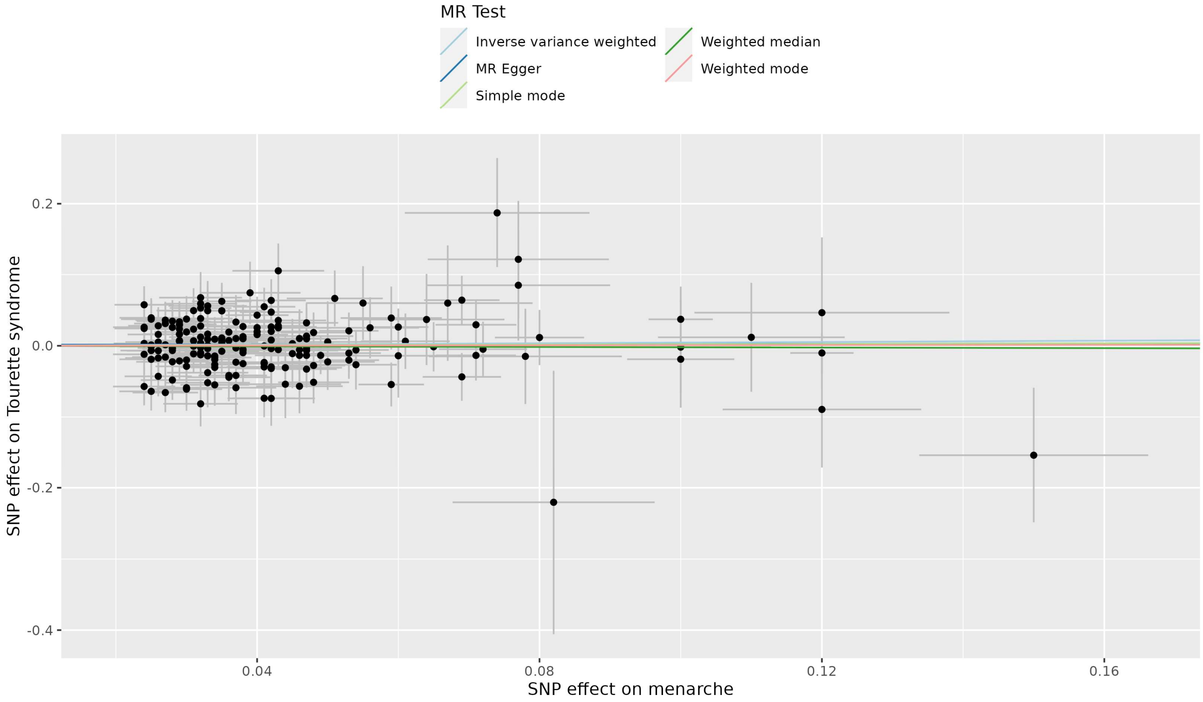


Scatter plot of menarche on Tourette syndrome

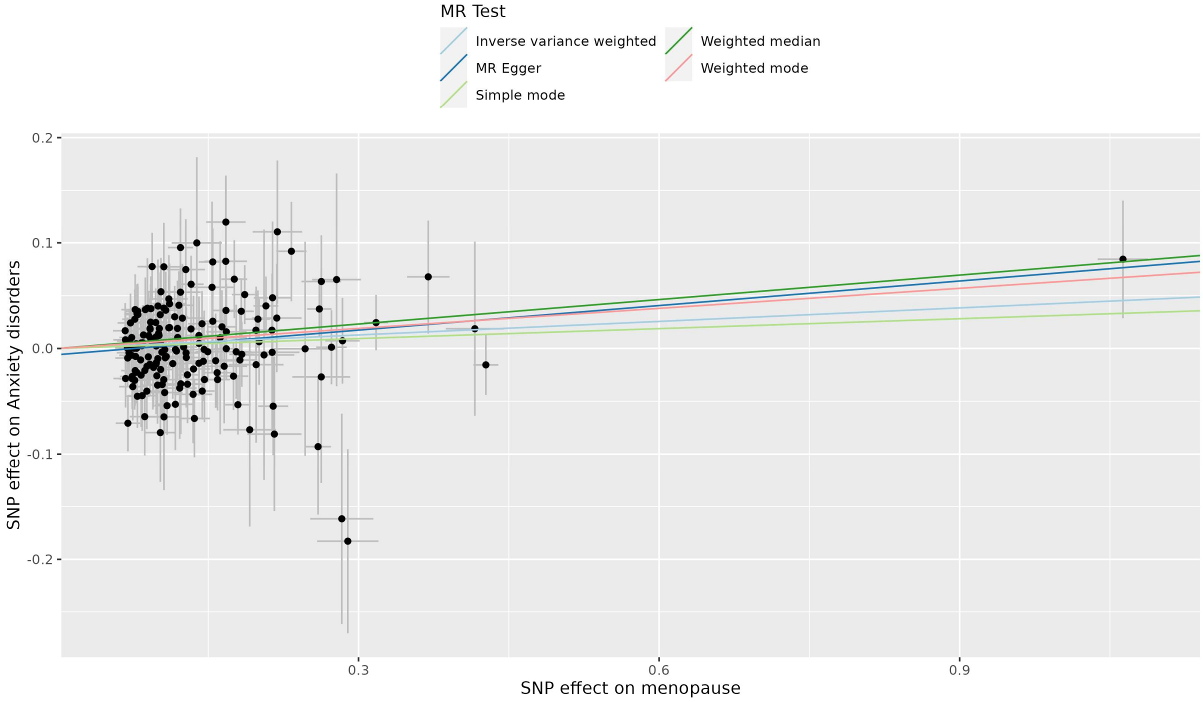


Scatter plot of menopause on Anxiety disorders

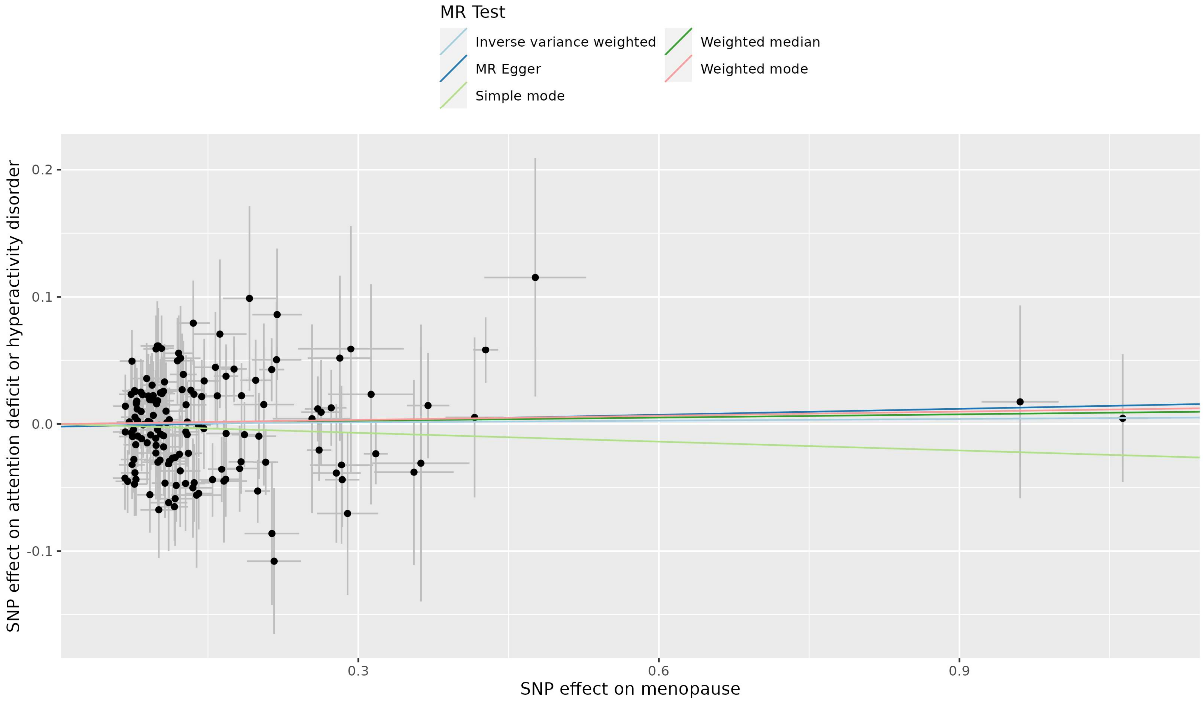


Scatter plot of menopause on attention deficit or hyperactivity disorder

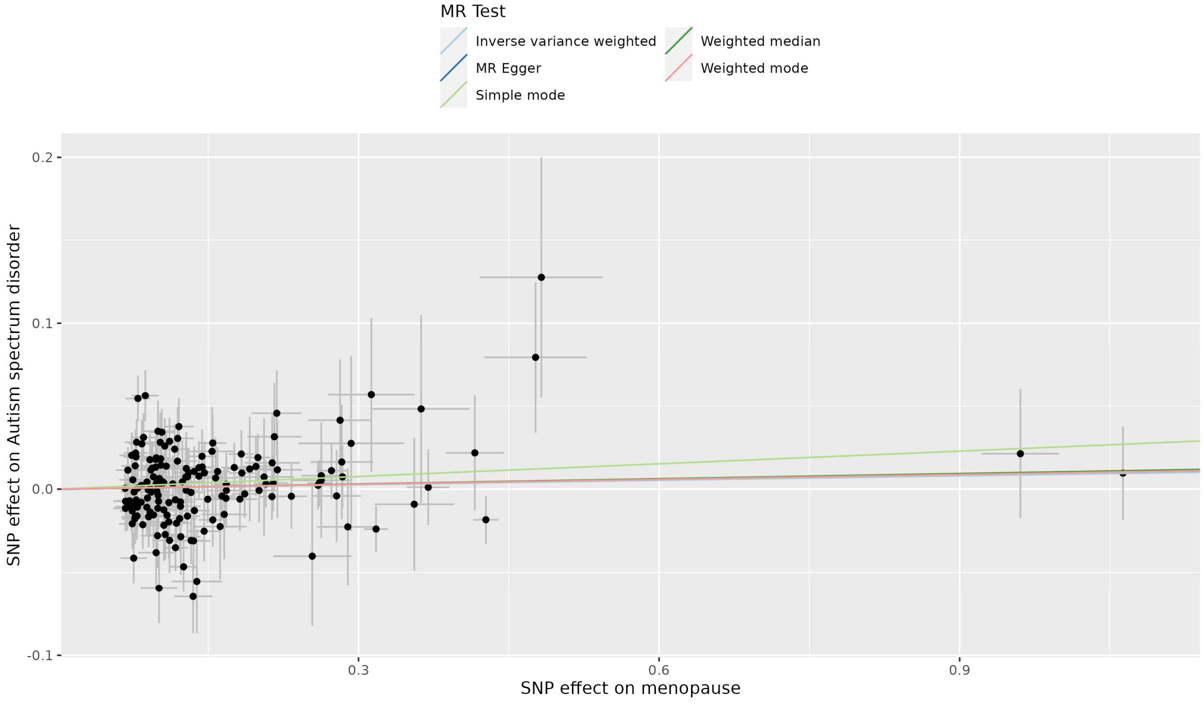


Scatter plot of menopause on Autism spectrum disorder

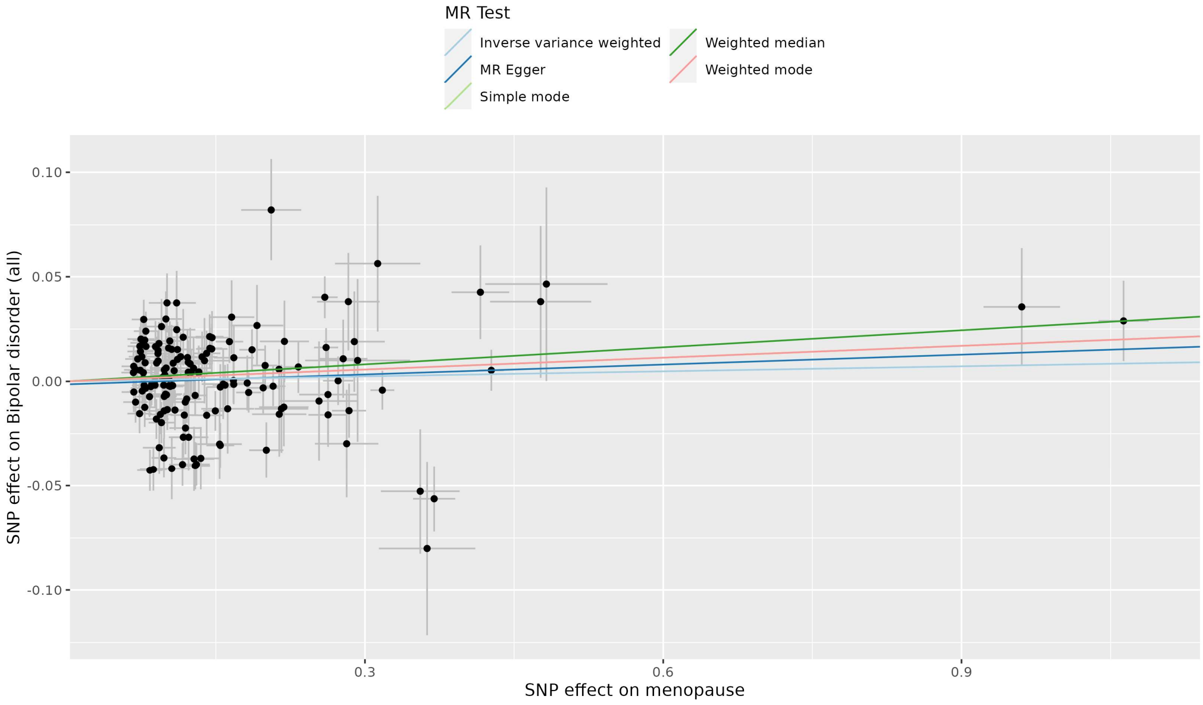


Scatter plot of menopause on Bipolar disorder (all)

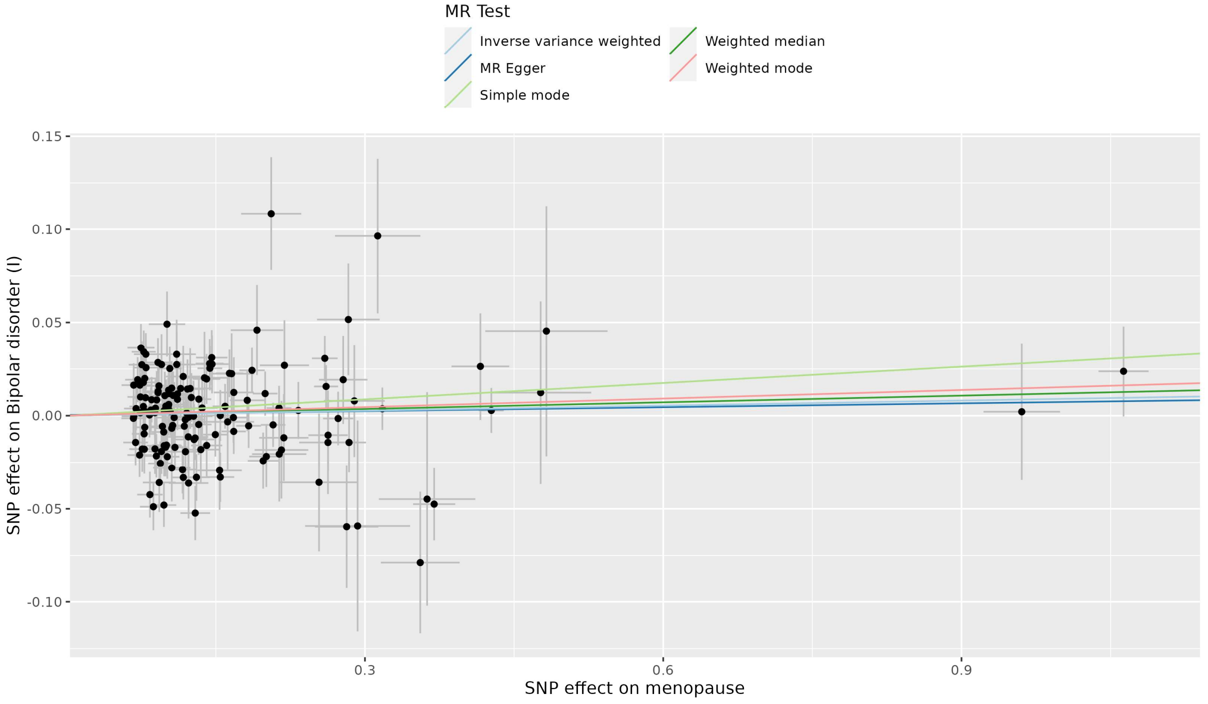


Scatter plot of menopause on Bipolar disorder (I)

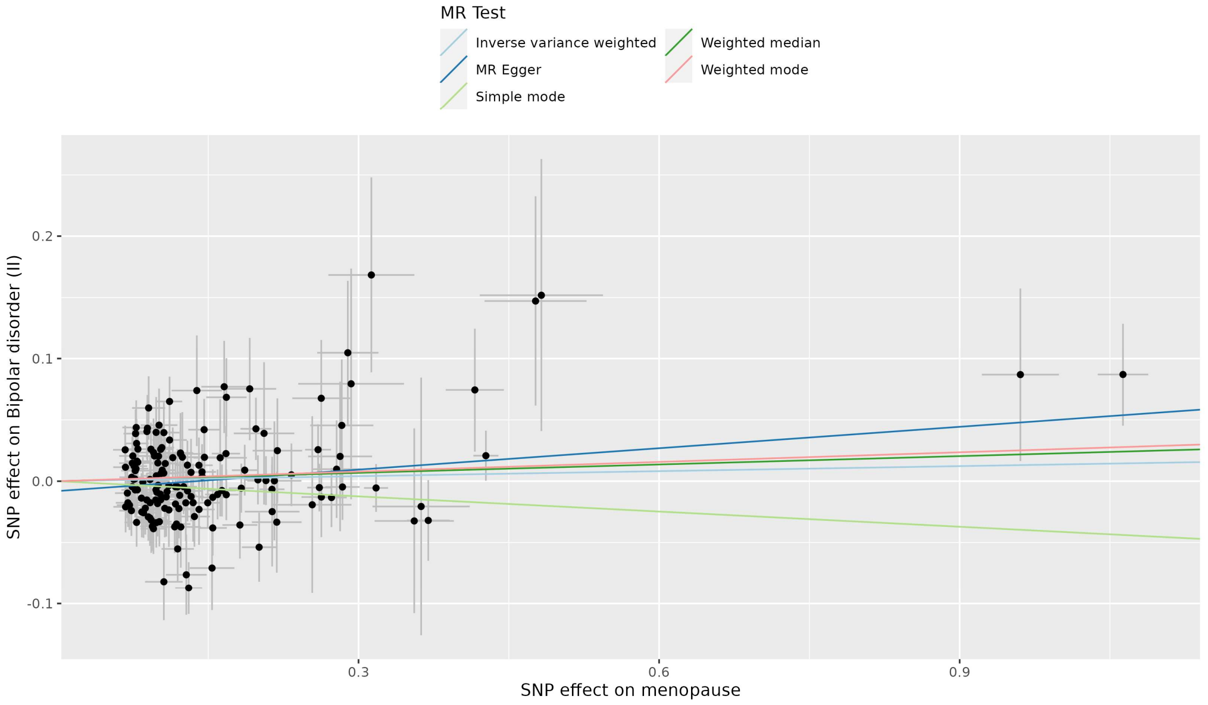


Scatter plot of menopause on Bipolar disorder (II)

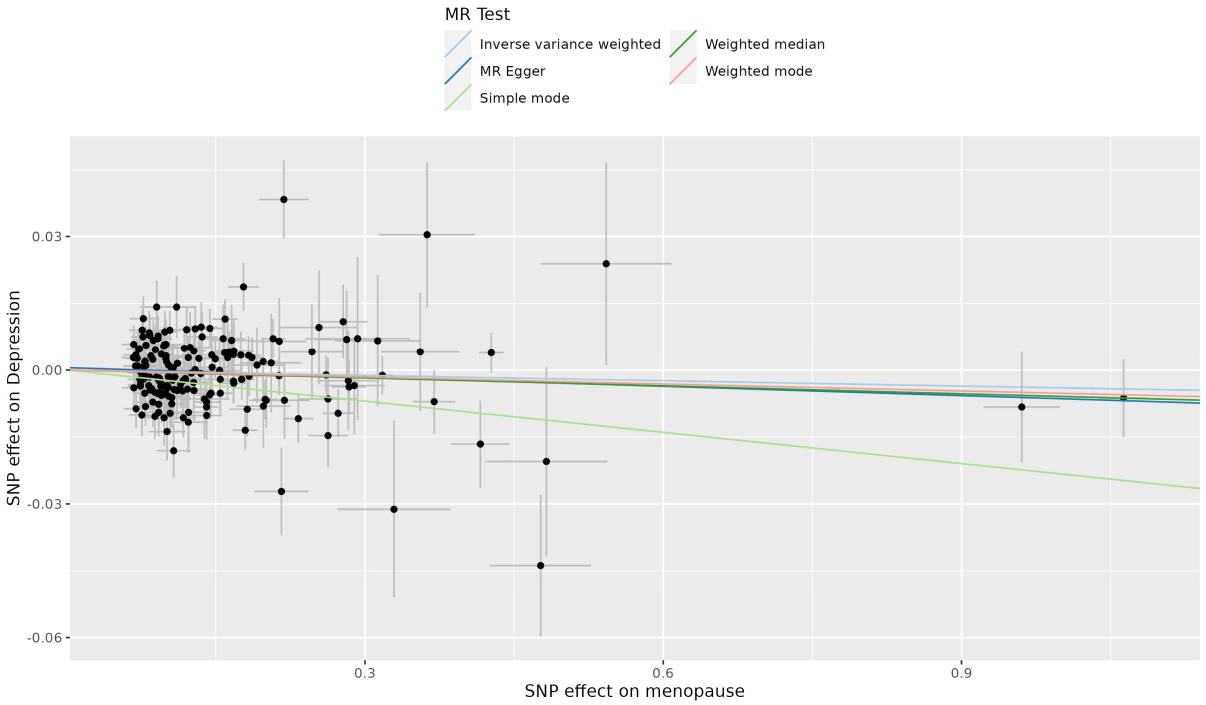


Scatter plot of menopause on Depression

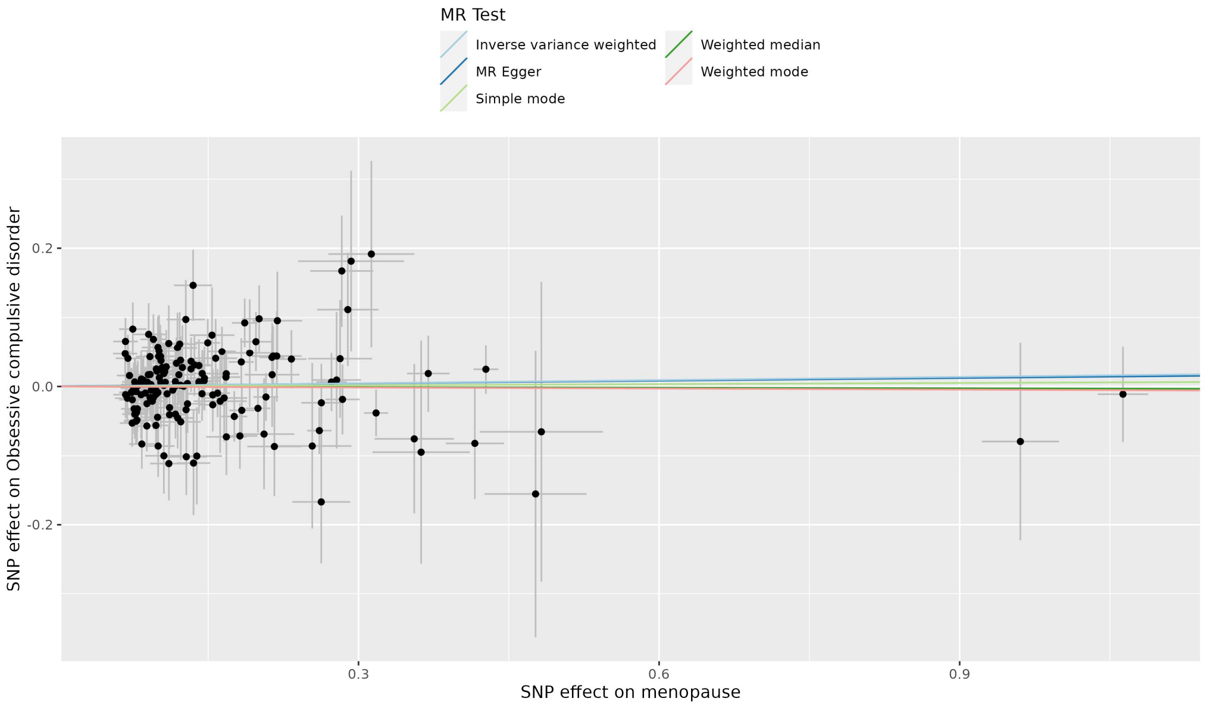


Scatter plot of menopause on Obsessive compulsive disorder

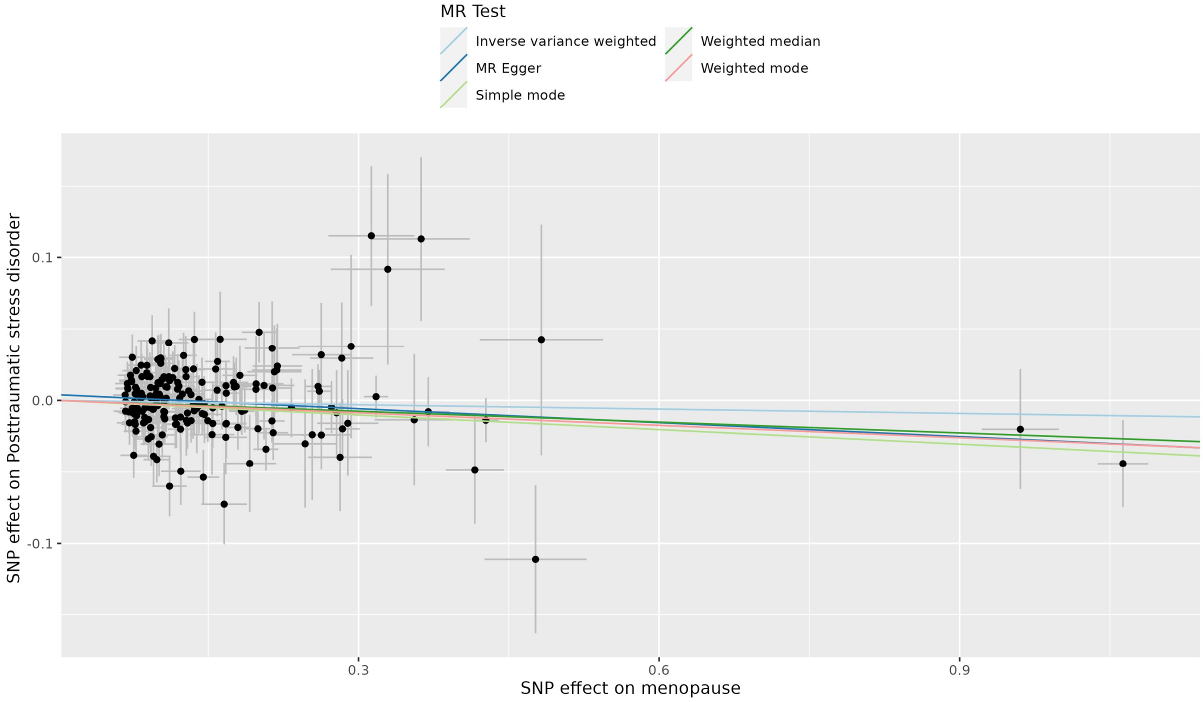


Scatter plot of menopause on Posttraumatic stress disorder

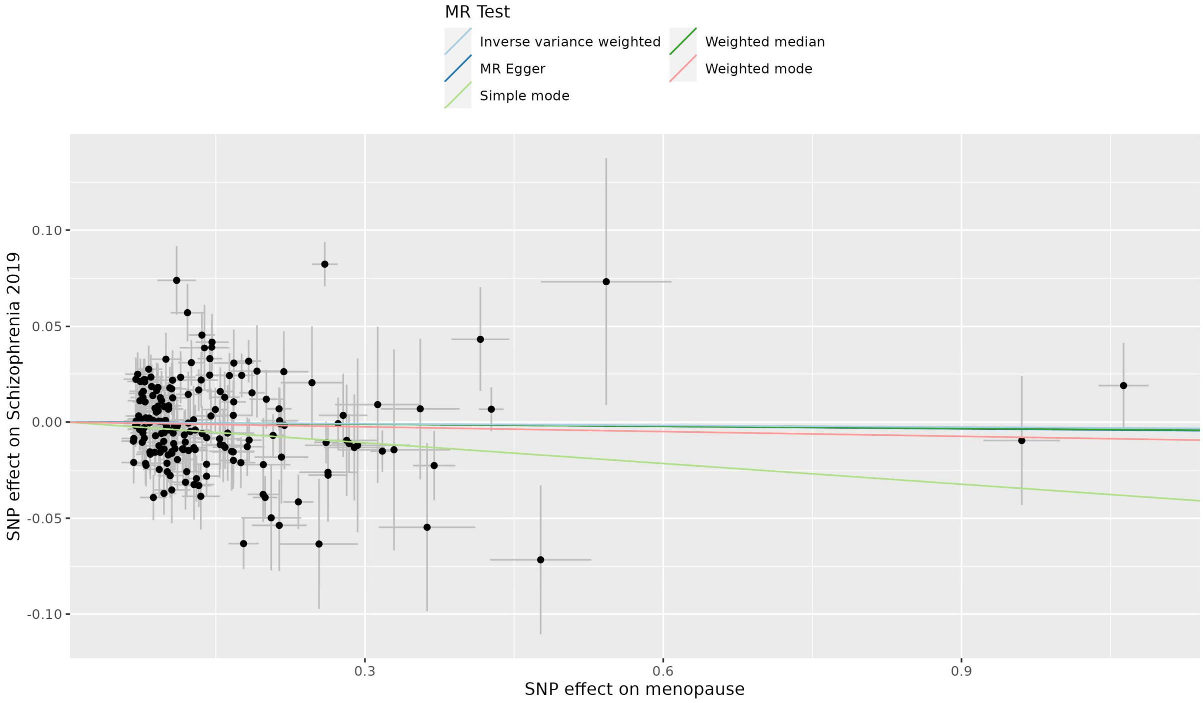


Scatter plot of menopause on Schizophrenia 2019

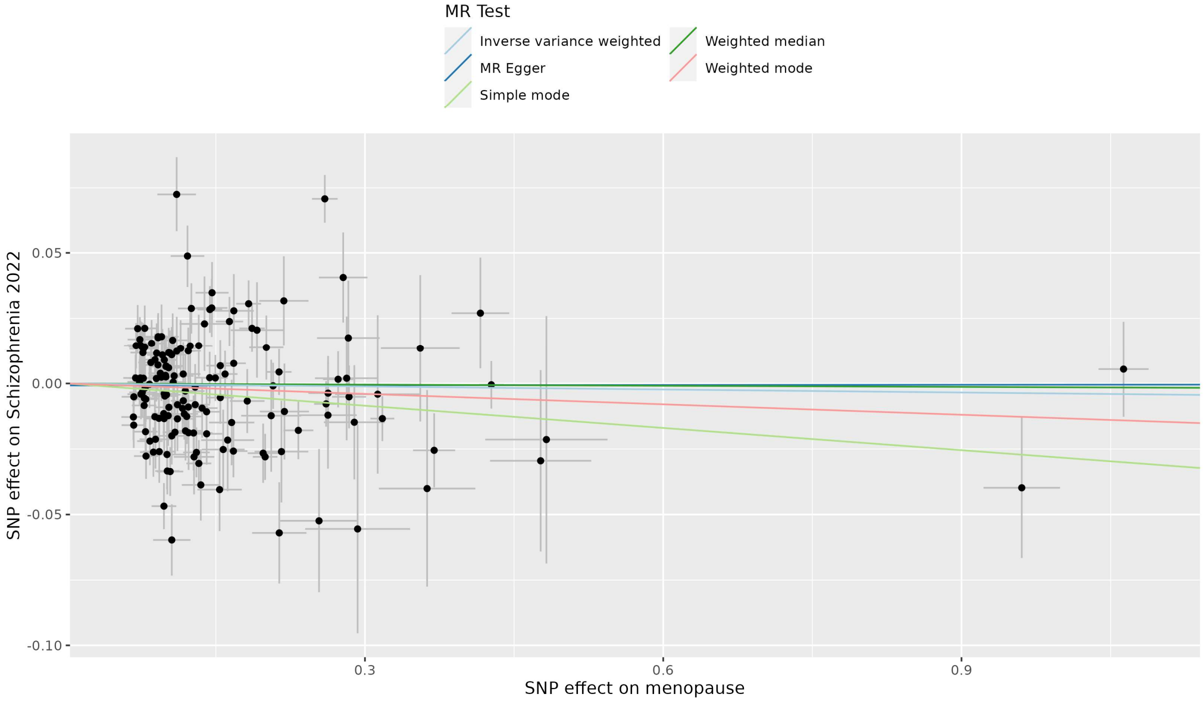


Scatter plot of menopause on Schizophrenia 2022

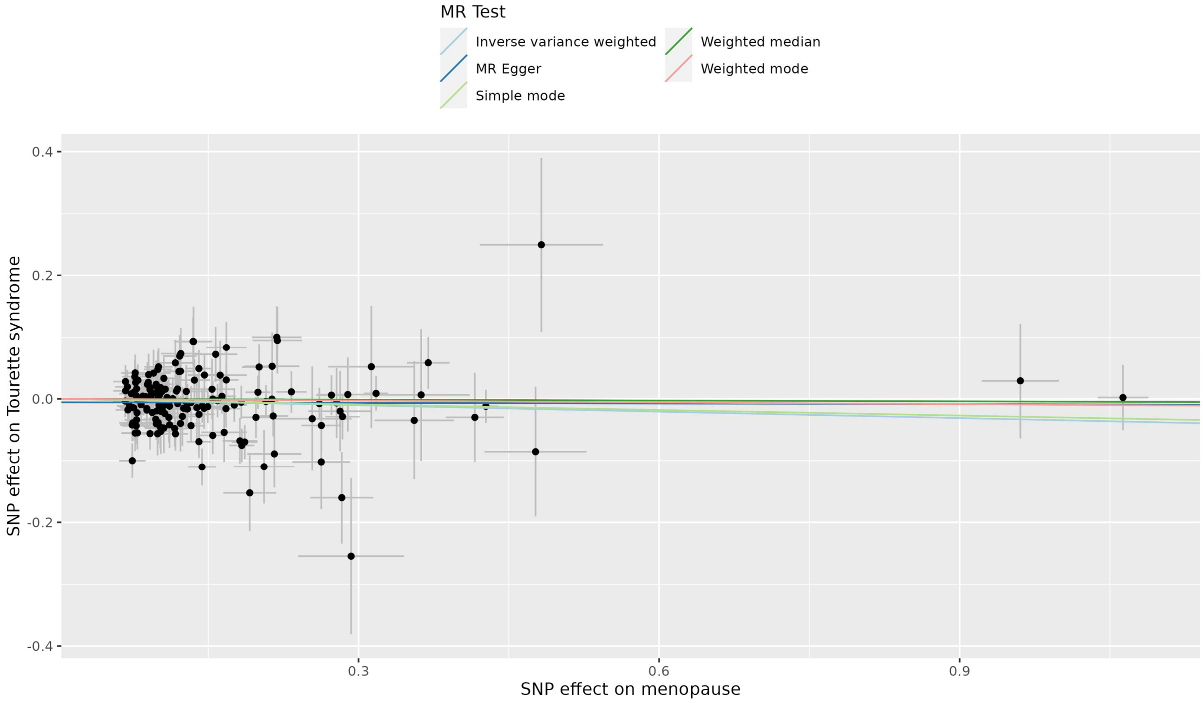


Scatter plot of menopause on Tourette syndrome

**Figure S13. Regression scatter plots for MR analysis**
